# Hopf Bifurcation in an Incommensurate Caputo Fractional-Order Computer Virus Epidemic Model with Multiple Time Delays

**DOI:** 10.3390/e28070787

**Published:** 2026-07-12

**Authors:** Ailing Zhong, Chengqiang Wang

**Affiliations:** 1 School of Computer Science and Technology, Tongji University, Shanghai 201804, China; 2353235@tongji.edu.cn; 2 School of Mathematics and Physics, Suqian University, Suqian 223800, China

**Keywords:** Hopf bifurcation, Caputo fractional-order derivative, computer virus epidemic spreading, Susceptible–Latent–Breaking-Out compartmental model, multiple time delays

## Abstract

Complex nonlinear dynamical systems, often associated with high-entropy time series, have been widely employed to describe and predict intricate dynamic phenomena in real-world systems. Motivated by the need to better understand such complex dynamics in network-based epidemic processes, this paper investigates bifurcation dynamics in a fractional-order extension of the classical Susceptible–Latent–Breaking–Out model for computer virus propagation. The proposed framework incorporates two distinct transmission-related time delays and employs Caputo fractional derivatives of incommensurate orders, with the delays associated with infection rate and latent period selected as the primary bifurcation parameters. Due to the combined influence of multiple delays and incommensurate fractional exponents, the resulting system exhibits a complexity that goes beyond most existing models in the literature. By linearizing the model around its endemic equilibrium and analyzing the associated characteristic roots, we characterize how the system’s qualitative behavior depends on the magnitudes of the time delays, and establish explicit sufficient conditions for bifurcation to occur. In particular, the endemic equilibrium remains asymptotically stable as long as each delay stays below a certain critical value; once any delay exceeds its threshold, the system undergoes a Hopf bifurcation, leading to sustained periodic oscillations in virus prevalence. Numerical simulations are provided to support the analytical results, and they show strong agreement between predicted and observed system responses. These findings enhance theoretical insight into bifurcation mechanisms in fractional-order delay models of epidemic dynamics on networks, and may offer useful guidance for designing containment strategies in large-scale interconnected systems.

## 1. Introduction

A broad range of computer viruses pose a significant threat to the safety of large-scale networks [1,2,3,4,5]. This category of malicious software includes conventional viruses and network-spreading worms, as well as other hazardous codes such as Trojan programs and spyware. Moreover, the pervasive and rapidly evolving nature of these threats imposes substantial socioeconomic costs, including massive financial losses, critical infrastructure disruption, the erosion of digital privacy, and systemic cybersecurity vulnerabilities [6,7,8].

In the past several decades, extensive efforts have been devoted to developing mathematical models that characterize the propagation of computer viruses, due to their rapid spread, significant economic impact, and potential threats to information security in modern networked systems; see [1,2,3,4,5,6,7,8,9,10,11,12,13,14,15,16,17], among the vast references. Early contributions primarily focused on compartmental modeling frameworks, which describe virus transmission among different system states; see, for example, [1,2,3,4]. These foundational models have provided important insights into the basic mechanisms governing virus spread. To better reflect realistic propagation processes, a variety of extensions have been proposed. For example, models incorporating time delays and more complex compartmental structures have been widely investigated; see [5,6,7]. Models accounting for spatial effects and network environments, such as reaction–diffusion systems and wireless network settings, have also been developed to capture more complex transmission patterns; see [8]. Recent studies have introduced additional modeling features, including stochastic effects, fractional-order dynamics, and saturation mechanisms, to enhance the descriptive capability of computer virus models; see [9,10,11,12,13]. Issues such as chaos, control, and synchronization have also been explored within the context of computer virus propagation models; see [14,15,16,17].

The further analysis of the models, proposed in References [1,2,3,4,5,6,7,8,9,10,11,12,13,14,15,16,17], not only facilitates further study of the underlying mechanisms of viral spread but also guides the design of further treatment and intervention measures. For example, via employing the compartmental framework commonly used in epidemic modeling, Yang et al. [1] proposed the following computer virus model with graded cure rates (SLB):

(1) $$\left\{\begin{array}{cccc} \frac{dS(t)}{dt} & =\mu -\beta S(t)(L(t)+B(t))+\gamma L(t)+\delta B(t)-\mu S(t) & \; & \mathrm{for}\;t\in R_{+},\\ \frac{dL(t)}{dt} & =\beta S(t)(L(t)+B(t))-\gamma L(t)-\varepsilon L(t)-\mu L(t) & \; & \mathrm{for}\;t\in R_{+},\\ \frac{dB(t)}{dt} & =\varepsilon L(t)-\delta B(t)-\mu B(t) & \; & \mathrm{for}\;t\in R_{+}, \end{array}\right.$$

where S(t), L(t), and B(t) denote, at time *t*, the percentages of computers in the internal network that are susceptible, latent, and breaking-out with respect to computer virus infection, respectively. In SLB (1), μ denotes the constant rate at which external (virus-free) computers connect to the internal network, which also equals the rate at which internal computers disconnect from the network, ensuring that the total number of computers in the internal network remains constant; β is the effective contact rate, quantifying the transmission probability per contact between a susceptible computer and an infectious computer (either latent or breaking-out); γ represents the cure rate for latent computers, meaning that latent computers return to the susceptible class at this constant rate; δ denotes the cure rate for breaking-out computers, i.e., actively spreading computers return to the susceptible class at this constant rate; and ε is the rate at which latent computers break out and transition to the actively infectious (breaking-out) class. Finally, following the hypothesis of graded cure rates, as with Reference [1], we require δ>γ>0, meaning that breaking-out computers are cured at a higher rate than latent computers. In SLB (1) and hereafter, we denote $R_{+}=\left(0,+\infty \right)$. Among these classes of computer virus models, an important subclass is that involving fractional-order derivatives; see [9,10,11,12,13,14,15].

Various types of fractional-order derivatives have been widely incorporated into the modeling and analysis of dynamical systems across diverse research areas, owing to their ability to capture memory and hereditary effects. In particular, related investigations have been carried out in the broader context of epidemic and virus-related systems, where fractional-order approaches have been used to analyze stability, bifurcation, and control problems; see [18,19,20,21,22]. Beyond virus propagation, fractional-order models have also been extensively applied to complex dynamical systems such as neural networks [23,24,25,26,27,28,29,30], ecological systems [31,32,33,34], and economic systems [35]. As mentioned just now, a substantial body of work has focused on computer virus propagation models, where fractional-order formulations have been employed to investigate dynamical behaviors such as stability, bifurcation, chaos, and control; see, for example, [9,11,13,14,15,36,37,38,39].

It is widely recognized that, in addition to fractional-order derivatives, the incorporation of time delays serves as an effective alternative for modeling after-effects or memory in dynamical systems. In recent years, extensive efforts have been devoted to the modeling and analysis of various types of dynamical systems, particularly those incorporating time-delay effects, which are essential for capturing realistic temporal processes; see [23,24,25,26,27,28,30,35] for representative results on neural network models, and [33,34,40,41,42,43,44,45] for related studies on ecological, financial, and other applied dynamical systems. In addition, epidemic and virus-related models have been extensively investigated, where time delays and nonlinear interactions play a crucial role in determining system behavior; see [18,20,21,36,46]. These studies provide important insights into stability, bifurcation, and control mechanisms in transmission dynamics. As with other transmission systems, by incorporating latency and delayed responses, computer virus models often exhibit complex dynamical behaviors, including stability switching and Hopf bifurcation; see [5,6,7,47,48,49,50,51,52].

Among the complex dynamics exhibited by computer virus propagation models, bifurcation is recognized as one of the most significant phenomena, particularly when it is induced by time delays. Bifurcation analysis not only deepens the theoretical understanding of the dynamic evolution of the system, but also plays an important role in revealing and characterizing the intrinsic dynamical behaviors of the system, including stability switches, oscillatory patterns, and complex nonlinear phenomena; see [53,54,55]. Delays, which naturally arise from latency, incubation, or response mechanisms in networks, can fundamentally alter system stability and lead to qualitative changes in system behavior. This delay-induced bifurcation phenomenon has been widely reported in computer virus and related epidemic-type models; see, for instance, [6,47,48,49,51,52,56]. In general, bifurcation refers to a critical transition in which a small variation in a system parameter (such as a time delay) causes a qualitative change in the structure of equilibria or periodic solutions. In particular, Hopf bifurcation plays a crucial role, as it marks the onset of sustained oscillations emerging from an equilibrium state. Such oscillatory behaviors are frequently observed in a broad class of dynamical systems, including neural networks, ecological systems, financial systems, and fractional-order systems; see, for example, [20,21,23,25,26,27,28,30,32,35,36,40,43,45,57,58,59]. These studies demonstrate that bifurcation phenomena are universal and play a fundamental role in shaping the dynamical behaviors of complex systems. For computer virus models, bifurcation analysis provides essential insights into the mechanisms underlying complex behaviors such as periodic outbreaks, persistent oscillations, and even chaotic dynamics. For example, delay-induced Hopf bifurcation has been shown to generate periodic oscillations in virus propagation, reflecting recurrent infection waves in networks [49,51,52,56]. Among delayed computer virus models, Zhang and Bi [50] investigated a delayed SLB model (DSLB) which provides important motivation for the present study. In particular, they considered a time-delayed variant of SLB (1), namely

(2) $$\left\{\begin{array}{cccc} \frac{dS(t)}{dt} & =\mu -\beta S(t)(L(t)+B(t))+\gamma L(t-\tau )+\delta B(t-\tau )-\mu S(t) & \; & \mathrm{for}\;t\in R_{+},\\ \frac{dL(t)}{dt} & =\beta S\left(t\right)\left(L(t)+B(t)\right)-\gamma L\left(t-\tau \right)-\varepsilon L\left(t\right)-\mu L\left(t\right) & \; & \mathrm{for}\;t\in R_{+},\\ \frac{dB(t)}{dt} & =\varepsilon L(t)-\delta B(t-\tau )-\mu B(t) & \; & \mathrm{for}\;t\in R_{+}, \end{array}\right.$$

where $\tau \in R_{+}$ is taken as the bifurcation parameter. Zhang and Bi [50] established that DSLB (2) undergoes a Hopf bifurcation as τ passes through a critical threshold.

**Figure 1:**
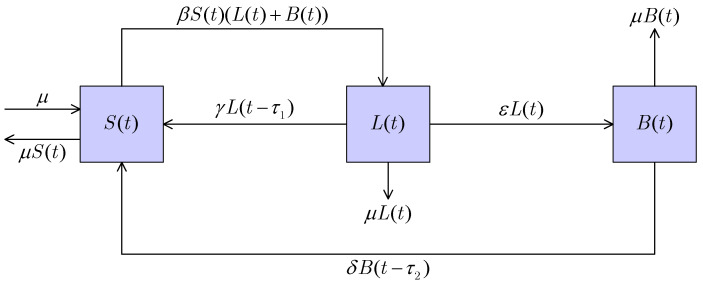
Transmission diagram of the computer virus model (3).

A review of the existing literature reveals that most available models incorporate at most a single time delay (see [50], for instance), which may be insufficient to adequately capture the realistic propagation mechanisms of computer viruses. In practice, different compartments of the computer virus models are often subject to distinct delay effects, and neglecting this heterogeneity may lead to an incomplete description of the underlying dynamics. The majority of fractional-order computer virus models in the literature are restricted to the commensurate case. Such formulations fail to account for the possibility that different state variables may exhibit heterogeneous memory characteristics. In addition, bifurcation analysis for incommensurate fractional-order computer virus models is still not widely covered in the current literature and may warrant further investigation. Motivated by the need to address these deficiencies, we focus on the bifurcation problem (with time delays acting as bifurcation parameters) of the following fractional-order SLB with two distinct time delays (DFSLB, for short; see Figure 1 for the transmission diagram):

(3) Dtα1CS(t)=μ−βS(t)(L(t)+B(t))+γL(t−τ1)+δB(t−τ2)−μS(t)fort∈R+,Dtα2CL(t)=βS(t)(L(t)+B(t))−γL(t−τ1)−εL(t)−μL(t)fort∈R+,Dtα3CB(t)=εL(t)−δB(t−τ2)−μB(t)fort∈R+,

where DtαC=Dtαt0C with $t_{0}=0$ denotes the time-fractional derivative in the Caputo sense, $\alpha_{i}\in \left(0,1\right]$(i=1,2,3) are the corresponding fractional orders, and $\tau_{1},\tau_{2}\in R_{+}$ represent the time delays. The state variable (S(t),L(t),B(t)) and the parameters β, γ, δ, μ, and ε have the same physical interpretations as those in SLB (1) and its delayed counterpart (2).

For completeness, we briefly recall its definition as given in Reference [60].

**Definition** **1** ([60])**.** *Given a real number α>0, let n denote the unique positive integer satisfying n−1<α⩽n. Suppose that the function φ(t) is defined on $\left[t_{0},\infty \right)$ and possesses n continuous derivatives. The Caputo-type fractional derivative of order α of φ(t) is defined as*

(4) Dtαt0Cφ(t)=1Γ(n−α)∫t0tφ(n)(s)(t−s)α+1−nds,whenevert∈[t0,+∞),

*where $t_{0}\in R$ is the initial time point, and Γ(·) stands for the standard Gamma function, defined by*

(5) $$\Gamma \left(z\right)=\int_{0}^{+\infty }t^{z-1}e^{-t}\;dt.$$

Our main contributions and innovations are summarized as follows:

- The computer virus model (3) considered in this paper incorporates two distinct time delays. In contrast to several existing models in the literature, the introduction of multiple delays better reflects the realistic situation in which different compartments of the system may involve different delay effects. Moreover, the presence of distinct delays enriches the dynamical behavior of the system, thereby allowing for more flexible and realistic applications. In particular, the interaction between these delays may give rise to more complex phenomena, such as stability switching and multiple bifurcation scenarios. This further enhances the applicability of the model in describing real-world virus propagation processes and provides a more comprehensive framework for understanding the influence of delay effects on system dynamics.
- The model (3) is formulated as a fractional-order system with incommensurate orders in the Caputo sense. As indicated previously, the utilization of incommensurate fractional derivatives enhances the modeling capability by capturing memory and hereditary effects with greater flexibility, allowing different state variables to exhibit distinct memory characteristics. Compared with commensurate fractional-order models, this formulation provides a more general and realistic framework for describing complex dynamical processes. However, it also introduces significant mathematical challenges, particularly in the bifurcation analysis, due to the increased complexity of the characteristic equations, the lack of a unified fractional order, and the intricate stability conditions. These features make the analytical treatment more involved, while simultaneously enriching the potential dynamical behaviors of the system.
- The model (3) under consideration describes the propagation of computer viruses. We establish two results concerning the impact of two distinct time delays on the system dynamics and demonstrate that these delays can induce Hopf bifurcation in DFSLB (3). The analysis is conducted via linearization and the study of the associated characteristic equations, which provide explicit conditions for stability switching. Several numerical simulations are also conducted to validate and support the theoretical findings. The results obtained in this paper provide further insight into the mechanisms governing bifurcation phenomena in delayed fractional-order (incommensurate or not) computer virus models, and may facilitate the development of effective control strategies for mitigating virus propagation in complex networked systems.

As mentioned above, this paper addresses the bifurcation problem of DFSLB (3). In bifurcation theory, equilibria and their bifurcations are the central objects of investigation, among other things. The equilibria and periodic orbits (trajectories), represent fundamental classes of solutions to DFSLB (3). To formalize the problem setup for the system dynamics, we define the initial state profiles on the time history interval for DFSLB (3). Specifically, the solution is complemented by the following initial data:

(6) $$\left.\begin{array}{cccc} \; & S\left(t\right)=S_{0}\left(t\right) & \; & \mathrm{for}\;t\in [-\tau_{\max},0],\\ \; & L\left(t\right)=L_{0}\left(t\right) & \; & \mathrm{for}\;t\in [-\tau_{1},0],\\ \; & B\left(t\right)=B_{0}\left(t\right) & \; & \mathrm{for}\;t\in [-\tau_{2},0], \end{array}\right\}$$

in which $\tau_{\max}=\max\left(\tau_{1},\tau_{2}\right)$, $S_{0}\left(t\right):\left[-\tau_{\max},0\right]\to R_{+}$, $L_{0}\left(t\right):\left[-\tau_{1},0\right]\to R_{+}$, and $B_{0}\left(t\right):\left[-\tau_{2},0\right]\to R_{+}$ are given continuous functions. We are now in a position to present the precise definition of solutions to DFSLB (3) adopted in this paper.

**Definition** **2.** *The triple (S,L,B), consisting of continuous functions $S\left(t\right):[-\max\left(\tau_{1},\tau_{2}\right),+\infty )$$\to R_{+}$, $L\left(t\right):\left[-\tau_{1},+\infty \right)\to R_{+}$, and $B\left(t\right):\left[-\tau_{2},+\infty \right)\to R_{+}$, is called a solution of DFSLB (3) subject to the initial condition (6) if it satisfies the following system of Volterra integral equations:*

(7) $$\left\{\begin{array}{ccc} S\left(t\right)=S_{0}\left(0\right)+\frac{1}{\Gamma (\alpha_{1})}\int_{0}^{t}{\left(t-s\right)}^{\alpha_{1}-1}\left(\mu -\beta S\left(s\right)\left(L\left(s\right)+B\left(s\right)\right)+\gamma L\left(s-\tau_{1}\right)+\delta B\left(s-\tau_{2}\right)-\mu S\left(s\right)\right)ds & \; & \mathrm{for}\;t\in R_{+},\\ L\left(t\right)=L_{0}\left(0\right)+\frac{1}{\Gamma (\alpha_{2})}\int_{0}^{t}{\left(t-s\right)}^{\alpha_{2}-1}\left(\beta S\left(s\right)\left(L\left(s\right)+B\left(s\right)\right)-\gamma L\left(s-\tau_{1}\right)-\varepsilon L\left(s\right)-\mu L\left(s\right)\right)ds & \; & \mathrm{for}\;t\in R_{+},\\ B\left(t\right)=B_{0}\left(0\right)+\frac{1}{\Gamma (\alpha_{3})}\int_{0}^{t}{\left(t-s\right)}^{\alpha_{3}-1}\left(\varepsilon L\left(s\right)-\delta B\left(s-\tau_{2}\right)-\mu B\left(s\right)\right)ds & \; & \mathrm{for}\;t\in R_{+}. \end{array}\right.$$

**Remark** **1.** *Following a contraction mapping argument, together with Duhamel’s principle for Caputo fractional differential equations (see Reference [28] for details and implementation of this approach), one can rigorously prove that, for any given continuous functions $S_{0}\left(t\right):\left[-\max\left(\tau_{1},\tau_{2}\right),0\right]$$\to R_{+}$, $L_{0}\left(t\right):\left[-\tau_{1},0\right]\to R_{+}$, and $B_{0}\left(t\right):\left[-\tau_{2},0\right]\to R_{+}$, DFSLB (3) admits a unique solution subject to the initial condition (6) in the sense of Definition 2.*

Apart from Section 1, which provides the background, motivation, and necessary preliminaries for the problem under consideration, the remainder of the paper is organized into four sections. In Section 2, we investigate the equilibria (in particular, the endemic equilibrium) and their stability properties, which constitute a fundamental component of the bifurcation analysis. In Section 3, we present the main bifurcation results together with their rigorous proofs. In Section 4, we illustrate the theoretical results obtained in Section 3 by means of several numerical simulations based on two specific examples. Finally, in Section 5, we conclude the paper with several remarks.

## 2. Equilibria of DFSLB (3) and the Basic Reproduction Number

It can be readily verified that SLB (1), DSLB (2), and DFSLB (3) share a common equilibrium $\left(S^{*},L^{*},B^{*}\right)$, which is the solution of the following system of algebraic equations:

(8) $$\left\{\begin{array}{c} \mu -\beta S(L+B)+\gamma L+\delta B-\mu S=0,\\ \beta S(L+B)-\gamma L-\varepsilon L-\mu L=0,\\ \varepsilon L-\delta B-\mu B=0. \end{array}\right.$$

It is straightforward to show that SLB (1), DSLB (2), and DFSLB (3) share a common virus-free equilibrium

(9) $$\left(S^{*},L^{*},B^{*}\right)=\left(1,0,0\right).$$

Yang et al. [1] computed the basic reproduction number for SLB (1), to get

(10) $$R_{0}=\frac{\beta (\delta +\mu +\varepsilon )}{\left(\mu +\varepsilon +\gamma )(\delta +\mu \right)},$$

Yang et al. [1] further demonstrated that SLB (1) possesses a unique endemic equilibrium $\left(S^{*},L^{*},B^{*}\right)$. By definition, an endemic equilibrium requires that at least one of $L^{*}$ or $B^{*}$ be strictly positive; for SLB (1), this condition is equivalent to both being strictly positive and occurs if and only if $R_{0}>1$, with $R_{0}$ given by Equation (10). Moreover, through routine calculations, one can show that when $R_{0}>1$ (with $R_{0}$ given by Equation (10) and computed in [1]), the SLB (1), DSLB (2), and DFSLB (3) share a common endemic equilibrium $\left(S^{*},L^{*},B^{*}\right)$, with the components $S^{*}$, $L^{*}$, and $B^{*}$ given respectively by

(11) $$S^{*}=\frac{1}{R_{0}}=\frac{\left(\mu +\varepsilon +\gamma )(\delta +\mu \right)}{\beta (\delta +\mu +\varepsilon )},$$

(12) $$L^{*}=\frac{\delta +\mu }{\delta +\mu +\varepsilon }\left(1-\frac{1}{R_{0}}\right)=\frac{\delta +\mu }{\delta +\mu +\varepsilon }\left(1-\frac{\left(\mu +\varepsilon +\gamma )(\delta +\mu \right)}{\beta (\delta +\mu +\varepsilon )}\right),$$

and

(13) $$B^{*}=\frac{\varepsilon }{\delta +\mu +\varepsilon }\left(1-\frac{1}{R_{0}}\right)=\frac{\varepsilon }{\delta +\mu +\varepsilon }\left(1-\frac{\left(\mu +\varepsilon +\gamma )(\delta +\mu \right)}{\beta (\delta +\mu +\varepsilon )}\right).$$

The basic reproduction number $R_{0}$ for DSLB (2) and DFSLB (3), given by Equation (10), can be derived using the next-generation matrix approach. These models extend SLB (1), but the derivation follows essentially the same procedure as that for SLB (1). Since the primary objective of this paper is to investigate the bifurcation dynamics of DFSLB (3), we adopt Equation (10) as the corresponding threshold quantity throughout the subsequent analysis.

**Remark** **2.** *The basic reproduction number, denoted above by $R_{0}$ following the common notation in the literature, is defined as the expected number of secondary infections (or compromised units) generated by a single infected individual (or infectious agent) introduced into a completely susceptible population over the course of its infectious period, serving as a universal threshold parameter that determines whether the infection will die out ($R_{0}<1$) or persist and spread ($R_{0}>1$); see Reference [61] for the details. This concept applies across diverse domains: in epidemiological models (SIR, SEIR, SVEIR) it quantifies disease transmission among humans or animals; in computer virus propagation models (SLBS, SEIR-KS, SVEIR-KS) it measures the average number of computers or network nodes that a single infected machine compromises; in population dynamics models (predator–prey, competitive Lotka–Volterra, cooperative systems) it can be reinterpreted as a threshold for species invasion or coexistence; and in tumor-immune models it indicates the average number of new tumor cells generated by a single cancer cell before immune clearance, thereby unifying the stability analysis of fractional-order and delay differential systems across all these fields.*

## 3. Hopf Bifurcation Results and Their Proofs

In this section, our main objective is to investigate the Hopf bifurcation behavior of DFSLB (3). To this end, we first linearize DFSLB (3), which leads to the following system

(14) Dtα1CS(t)=−(βL*+βB*+μ)S(t)−βS*L(t)+γL(t−τ1)−βS*B(t)+δB(t−τ2)fort∈R+,Dtα2CL(t)=β(L*+B*)S(t)+(βS*−ε−μ)L(t)−γL(t−τ1)+βS*B(t)fort∈R+,Dtα3CB(t)=εL(t)−μB(t)−δB(t−τ2)fort∈R+,

or equivalently, to obtain

(15) Dtα1CS(t)=−(β−ε−γ)(δ+μ)+ε(β+μ)δ+μ+εS(t)−(μ+ε+γ)(δ+μ)δ+μ+εL(t)+γL(t−τ1)−(μ+ε+γ)(δ+μ)δ+μ+εB(t)+δB(t−τ2)fort∈R+,Dtα2CL(t)=β(δ+μ+ε)−(μ+ε+γ)(δ+μ)δ+μ+εS(t)+γ(δ+μ)−ε(ε+μ)δ+μ+εL(t)−γL(t−τ1)+(μ+ε+γ)(δ+μ)δ+μ+εB(t)fort∈R+,Dtα3CB(t)=εL(t)−μB(t)−δB(t−τ2)fort∈R+.

**Lemma** **1.** *Consider the linearization of DFSLB (3) as in Equation (14). Its characteristic equation takes the form $▽(\mu ;\tau_{1},\tau_{2})=0$, with ▽(μ;ϑ,υ) expressed explicitly as*

(16) $$\begin{array}{cc} ▽(\xi ;\vartheta ,\upsilon )= & \xi^{\alpha_{1}+\alpha_{2}+\alpha_{3}}+\left(\mu +\delta e^{-\upsilon \xi }\right)\xi^{\alpha_{1}+\alpha_{2}}+\left(\mu +\beta B^{*}+\beta L^{*}\right)\xi^{\alpha_{2}+\alpha_{3}}\\ \; & +\left(\varepsilon +\mu -\beta S^{*}+\gamma e^{-\vartheta \xi }\right)\xi^{\alpha_{3}+\alpha_{1}}+(\mu^{2}+\varepsilon \mu -\beta S^{*}\varepsilon -\beta S^{*}\mu \\ \; & +\gamma \mu e^{-\vartheta \xi }+\delta \varepsilon e^{-\upsilon \xi }+\delta \mu e^{-\upsilon \xi }-\beta S^{*}\delta e^{-\upsilon \xi }+\delta \gamma e^{-\xi (\vartheta +\upsilon )})\xi^{\alpha_{1}}\\ \; & +\left(\mu^{2}+\beta B^{*}\mu +\beta L^{*}\mu +\delta \mu e^{-\upsilon \xi }+\beta B^{*}\delta e^{-\upsilon \xi }+\beta L^{*}\delta e^{-\upsilon \xi }\right)\xi^{\alpha_{2}}\\ \; & +\left(\mu^{2}+\varepsilon \mu +\beta B^{*}\varepsilon +\beta B^{*}\mu +\beta L^{*}\varepsilon +\beta L^{*}\mu -\beta S^{*}\mu +\gamma \mu e^{-\vartheta \xi }\right)\xi^{\alpha_{3}}\\ \; & +\mu^{3}+\varepsilon \mu^{2}+B^{*}\beta \mu^{2}+L^{*}\beta \mu^{2}-S^{*}\beta \mu^{2}+B^{*}\beta \varepsilon \mu +L^{*}\beta \varepsilon \mu -S^{*}\beta \varepsilon \mu \\ \; & +\gamma \mu^{2}e^{-\vartheta \xi }+\delta \mu^{2}e^{-\upsilon \xi }+\delta \varepsilon \mu e^{-\upsilon \xi }+L^{*}\beta \delta \mu e^{-\upsilon \xi }-S^{*}\beta \delta \mu e^{-\upsilon \xi }\\ \; & +B^{*}\beta \delta \mu e^{-\upsilon \xi }+\delta \gamma \mu e^{-\xi (\vartheta +\upsilon )}. \end{array}$$

**Proof.**  By straightforward computation, we have

$$\begin{array}{cc} ▽(\xi ;\vartheta ,\upsilon )= & \det\left(\begin{array}{ccc} \xi^{\alpha_{1}}+\beta L^{*}+\beta B^{*}+\mu & \beta S^{*}-\gamma e^{-\tau_{1}\xi } & \beta S^{*}-\delta e^{-\tau_{2}\xi }\\ -\beta (L^{*}+B^{*}) & \xi^{\alpha_{2}}-\left(\beta S^{*}-\varepsilon -\mu \right)+\gamma e^{-\tau_{1}\xi } & -\beta S^{*}\\ 0 & -\varepsilon & \xi^{\alpha_{3}}+\mu +\delta e^{-\tau_{2}\xi } \end{array}\right)\\ = & \left(\xi^{\alpha_{3}}+\mu +\delta e^{-\tau_{2}\xi }\right)\det\left(\begin{array}{cc} \xi^{\alpha_{1}}+\beta L^{*}+\beta B^{*}+\mu & \beta S^{*}-\gamma e^{-\tau_{1}\xi }\\ -\beta (L^{*}+B^{*}) & \xi^{\alpha_{2}}-\left(\beta S^{*}-\varepsilon -\mu \right)+\gamma e^{-\tau_{1}\xi } \end{array}\right)\\ \; & +\varepsilon \det\left(\begin{array}{cc} \xi^{\alpha_{1}}+\beta L^{*}+\beta B^{*}+\mu & \beta S^{*}-\delta e^{-\tau_{2}\xi }\\ -\beta (L^{*}+B^{*}) & -\beta S^{*} \end{array}\right), \end{array}$$

which, along with some further simple calculations, completes the proof of Lemma 1. □

We now establish the following identity

(17) $${\left(\varrho i\right)}^{k_{1}\alpha_{1}+k_{2}\alpha_{2}+k_{3}\alpha_{3}}=\varrho^{k_{1}\alpha_{1}+k_{2}\alpha_{2}+k_{3}\alpha_{3}}e^{\frac{\pi (k_{1}\alpha_{1}+k_{2}\alpha_{2}+k_{3}\alpha_{3})i}{2}},$$

where ϱ∈[0,+∞), $\alpha_{1},\alpha_{2},\alpha_{3}\in \left(0,1\right]$, and $k_{1},k_{2},k_{3}\in N_{0}$. The relation (17) is of great importance and will be used repeatedly in the subsequent analysis.

Using Equation (17) as a key tool, for ▽(ξ;ϑ,υ) defined in (16), we formally obtain

(18) $$\begin{array}{cc} ▽(\varrho i;\vartheta ,\upsilon )= & \varrho^{\alpha_{1}+\alpha_{2}+\alpha_{3}}e^{\frac{\pi (\alpha_{1}+\alpha_{2}+\alpha_{3})i}{2}}+\left(\mu +\delta e^{-\upsilon \varrho i}\right)\varrho^{\alpha_{1}+\alpha_{2}}e^{\frac{\pi (\alpha_{1}+\alpha_{2})i}{2}}\\ \; & +\left(\mu +\beta B^{*}+\beta L^{*}\right)\varrho^{\alpha_{2}+\alpha_{3}}e^{\frac{\pi (\alpha_{2}+\alpha_{3})i}{2}}\\ \; & +\left(\varepsilon +\mu -\beta S^{*}+\gamma e^{-\vartheta \varrho i}\right)\varrho^{\alpha_{3}+\alpha_{1}}e^{\frac{\pi (\alpha_{3}+\alpha_{1})i}{2}}+(\mu^{2}+\varepsilon \mu -\beta S^{*}\varepsilon -\beta S^{*}\mu \\ \; & +\gamma \mu e^{-\vartheta \varrho i}+\delta \varepsilon e^{-\upsilon \varrho i}+\delta \mu e^{-\upsilon \varrho i}-\beta S^{*}\delta e^{-\upsilon \varrho i}+\delta \gamma e^{-\varrho (\vartheta +\upsilon )i})\varrho^{\alpha_{1}}e^{\frac{\pi \alpha_{1}i}{2}}\\ \; & +\left(\mu^{2}+\beta B^{*}\mu +\beta L^{*}\mu +\delta \mu e^{-\upsilon \varrho i}+\beta B^{*}\delta e^{-\upsilon \varrho i}+\beta L^{*}\delta e^{-\upsilon \varrho i}\right)\varrho^{\alpha_{2}}e^{\frac{\pi \alpha_{2}i}{2}}\\ \; & +\left(\mu^{2}+\varepsilon \mu +\beta B^{*}\varepsilon +\beta B^{*}\mu +\beta L^{*}\varepsilon +\beta L^{*}\mu -\beta S^{*}\mu +\gamma \mu e^{-\vartheta \varrho i}\right)\varrho^{\alpha_{3}}e^{\frac{\pi \alpha_{3}i}{2}}\\ \; & +\mu^{3}+\varepsilon \mu^{2}+B^{*}\beta \mu^{2}+L^{*}\beta \mu^{2}-S^{*}\beta \mu^{2}+B^{*}\beta \varepsilon \mu +L^{*}\beta \varepsilon \mu -S^{*}\beta \varepsilon \mu \\ \; & +\gamma \mu^{2}e^{-\vartheta \varrho i}+\delta \mu^{2}e^{-\upsilon \varrho i}+\delta \varepsilon \mu e^{-\upsilon \varrho i}+L^{*}\beta \delta \mu e^{-\upsilon \varrho i}-S^{*}\beta \delta \mu e^{-\upsilon \varrho i}\\ \; & +B^{*}\beta \delta \mu e^{-\upsilon \varrho i}+\delta \gamma \mu e^{-\varrho (\vartheta +\upsilon )i},\;\forall \varrho ,\vartheta ,\upsilon \in \left[0,+\infty \right). \end{array}$$

For the convenience of subsequent analysis, by direct computation, we evaluate the partial derivatives of ▽(ξ;ϑ,υ) with respect to ξ, ϑ, and υ, respectively, yielding

(19) $$\begin{array}{cc} \frac{\partial }{\partial \xi }▽\left(\xi ;\vartheta ,\upsilon \right)= & \left(\alpha_{1}+\alpha_{2}+\alpha_{3}\right)\xi^{\alpha_{1}+\alpha_{2}+\alpha_{3}-1}-\delta \upsilon e^{-\upsilon \xi }\xi^{\alpha_{1}+\alpha_{2}}+\left(\alpha_{1}+\alpha_{2}\right)\left(\mu +\delta e^{-\upsilon \xi }\right)\xi^{\alpha_{1}+\alpha_{2}-1}\\ \; & +\left(\alpha_{2}+\alpha_{3}\right)\left(\mu +\beta B^{*}+\beta L^{*}\right)\xi^{\alpha_{2}+\alpha_{3}-1}-\gamma \vartheta e^{-\vartheta \xi }\xi^{\alpha_{3}+\alpha_{1}}+\left(\alpha_{3}+\alpha_{1}\right)\left(\varepsilon +\mu -\beta S^{*}+\gamma e^{-\vartheta \xi }\right)\xi^{\alpha_{3}+\alpha_{1}-1}\\ \; & -\left(\gamma \mu \vartheta e^{-\vartheta \xi }+\delta \varepsilon \upsilon e^{-\upsilon \xi }+\delta \mu \upsilon e^{-\upsilon \xi }-\beta S^{*}\delta \upsilon e^{-\upsilon \xi }+\delta \gamma \left(\vartheta +\upsilon \right)e^{-\xi (\vartheta +\upsilon )}\right)\xi^{\alpha_{1}}\\ \; & +\alpha_{1}\left(\mu^{2}+\varepsilon \mu -\beta S^{*}\varepsilon -\beta S^{*}\mu +\gamma \mu e^{-\vartheta \xi }+\delta \varepsilon e^{-\upsilon \xi }+\delta \mu e^{-\upsilon \xi }-\beta S^{*}\delta e^{-\upsilon \xi }+\delta \gamma e^{-\xi (\vartheta +\upsilon )}\right)\xi^{\alpha_{1}-1}\\ \; & -\left(\delta \mu \upsilon e^{-\upsilon \xi }+\beta B^{*}\delta \upsilon e^{-\upsilon \xi }+\beta L^{*}\delta \upsilon e^{-\upsilon \xi }\right)\xi^{\alpha_{2}}\\ \; & +\alpha_{2}\left(\mu^{2}+\beta B^{*}\mu +\beta L^{*}\mu +\delta \mu e^{-\upsilon \xi }+\beta B^{*}\delta e^{-\upsilon \xi }+\beta L^{*}\delta e^{-\upsilon \xi }\right)\xi^{\alpha_{2}-1}\\ \; & -\gamma \mu \vartheta e^{-\vartheta \xi }\xi^{\alpha_{3}}+\alpha_{3}\left(\mu^{2}+\varepsilon \mu +\beta B^{*}\varepsilon +\beta B^{*}\mu +\beta L^{*}\varepsilon +\beta L^{*}\mu -\beta S^{*}\mu +\gamma \mu e^{-\vartheta \xi }\right)\xi^{\alpha_{3}-1}\\ \; & -(\gamma \mu^{2}\vartheta e^{-\vartheta \xi }+\delta \mu^{2}\upsilon e^{-\upsilon \xi }+\delta \varepsilon \mu \upsilon e^{-\upsilon \xi }+L^{*}\beta \delta \mu \upsilon e^{-\upsilon \xi }-S^{*}\beta \delta \mu \upsilon e^{-\upsilon \xi }\\ \; & \;\;+B^{*}\beta \delta \mu \upsilon e^{-\upsilon \xi }+\delta \gamma \mu \left(\vartheta +\upsilon \right)e^{-\xi (\vartheta +\upsilon )}), \end{array}$$

(20) $$\begin{array}{cc} \frac{\partial }{\partial \vartheta }▽\left(\xi ;\vartheta ,\upsilon \right)= & -\gamma e^{-\vartheta \xi }\xi^{\alpha_{3}+\alpha_{1}+1}-\left(\gamma \mu e^{-\vartheta \xi }+\delta \gamma e^{-\xi (\vartheta +\upsilon )}\right)\xi^{\alpha_{1}+1}\\ \; & -\gamma \mu e^{-\vartheta \xi }\xi^{\alpha_{3}+1}-\gamma \mu^{2}e^{-\vartheta \xi }\xi -\delta \gamma \mu e^{-\xi (\vartheta +\upsilon )}\xi , \end{array}$$

and

(21) $$\begin{array}{cc} \frac{\partial }{\partial \upsilon }▽\left(\xi ;\vartheta ,\upsilon \right)= & -\delta e^{-\upsilon \xi }\xi^{\alpha_{1}+\alpha_{2}+1}-\left(\delta \varepsilon e^{-\upsilon \xi }+\delta \mu e^{-\upsilon \xi }-\beta S^{*}\delta e^{-\upsilon \xi }+\delta \gamma e^{-\xi (\vartheta +\upsilon )}\right)\xi^{\alpha_{1}+1}\\ \; & -\left(\delta \mu e^{-\upsilon \xi }+\beta B^{*}\delta e^{-\upsilon \xi }+\beta L^{*}\delta e^{-\upsilon \xi }\right)\xi^{\alpha_{2}+1}\\ \; & -(\delta \mu^{2}e^{-\upsilon \xi }+\delta \varepsilon \mu e^{-\upsilon \xi }+L^{*}\beta \delta \mu e^{-\upsilon \xi }-S^{*}\beta \delta \mu e^{-\upsilon \xi }+B^{*}\beta \delta \mu e^{-\upsilon \xi }+\delta \gamma \mu e^{-\xi (\vartheta +\upsilon )})\xi . \end{array}$$

In order to state one of our principal theorems, let us set $W_{1}$, where

(22) $$\begin{array}{cc} W_{1}= & |\mu^{3}+\varepsilon \mu^{2}+B^{*}\beta \mu^{2}+L^{*}\beta \mu^{2}-S^{*}\beta \mu^{2}+B^{*}\beta \varepsilon \mu +L^{*}\beta \varepsilon \mu -S^{*}\beta \varepsilon \mu \\ \; & +\delta \mu^{2}+\delta \varepsilon \mu +L^{*}\beta \delta \mu -S^{*}\beta \delta \mu +B^{*}{\beta \delta \mu |}^{2}-\gamma^{2}\mu^{2}{\left(\mu +\delta \right)}^{2}, \end{array}$$

and additionally define $W_{2}\left(\varrho ,\vartheta \right)$ with

(23) $$\begin{array}{cc} W_{2}\left(\varrho ,\vartheta \right)= & \left(\alpha_{1}+\alpha_{2}+\alpha_{3}\right)\varrho^{2\alpha_{1}+\alpha_{2}+2\alpha_{3}}\cos\left(\frac{\pi \alpha_{2}}{2}+\vartheta \varrho \right)+\left(\alpha_{1}+\alpha_{2}\right)\left(\mu +\delta \right)\varrho^{2\alpha_{1}+\alpha_{2}+\alpha_{3}}\cos\left(\frac{\pi (\alpha_{2}-\alpha_{3})}{2}+\vartheta \varrho \right)\\ \; & +\left(\alpha_{2}+\alpha_{3}\right)\left(\mu +\beta B^{*}+\beta L^{*}\right)\varrho^{\alpha_{1}+\alpha_{2}+2\alpha_{3}}\cos\left(\frac{\pi (\alpha_{2}-\alpha_{1})}{2}+\vartheta \varrho \right)\\ \; & +\left(\alpha_{3}+\alpha_{1}\right)\left(\varepsilon +\mu -\beta S^{*}\right)\varrho^{2\alpha_{3}+2\alpha_{1}}+\gamma \left(\alpha_{3}+\alpha_{1}\right)\varrho^{2\alpha_{3}+2\alpha_{1}}\cos\left(\vartheta \varrho \right)\\ \; & +\alpha_{1}\left(\mu^{2}+\varepsilon \mu -\beta S^{*}\varepsilon -\beta S^{*}\mu +\delta \varepsilon +\delta \mu -\beta S^{*}\delta \right)\varrho^{\alpha_{3}+2\alpha_{1}}\cos\left(\vartheta \varrho -\frac{\pi \alpha_{3}}{2}\right)+\alpha_{1}\gamma \left(\mu +\delta \right)\varrho^{\alpha_{3}+2\alpha_{1}}\cos\left(\frac{\pi \alpha_{3}}{2}\right)\\ \; & +\alpha_{2}\left(\mu^{2}+\beta B^{*}\mu +\beta L^{*}\mu +\delta \mu +\beta B^{*}\delta +\beta L^{*}\delta \right)\varrho^{\alpha_{1}+\alpha_{2}+\alpha_{3}}\cos\left(\frac{\pi (\alpha_{2}-\alpha_{1})}{2}+\vartheta \varrho \right)\\ \; & +\alpha_{3}\left(\mu^{2}+\varepsilon \mu +\beta B^{*}\varepsilon +\beta B^{*}\mu +\beta L^{*}\varepsilon +\beta L^{*}\mu -\beta S^{*}\mu \right)\varrho^{2\alpha_{3}+\alpha_{1}}\cos\left(\vartheta \varrho -\frac{\pi \alpha_{1}}{2}\right)+\alpha_{3}\gamma \mu \varrho^{2\alpha_{3}+\alpha_{1}}\cos\left(\frac{\pi \alpha_{1}}{2}\right)\\ \; & +\left(\mu +\delta \right)\left(\alpha_{1}+\alpha_{2}+\alpha_{3}\right)\varrho^{\alpha_{1}+\alpha_{2}+\alpha_{3}}\cos\left(\frac{\pi (\alpha_{2}+\alpha_{3})}{2}+\vartheta \varrho \right)+\left(\alpha_{1}+\alpha_{2}\right){\left(\mu +\delta \right)}^{2}\varrho^{\alpha_{1}+\alpha_{2}}\cos\left(\vartheta \varrho +\frac{\pi \alpha_{2}}{2}\right)\\ \; & +\left(\mu +\delta \right)\left(\alpha_{2}+\alpha_{3}\right)\left(\mu +\beta B^{*}+\beta L^{*}\right)\varrho^{\alpha_{2}+\alpha_{3}}\cos\left(\frac{\pi (\alpha_{2}+\alpha_{3}-\alpha_{1})}{2}+\vartheta \varrho \right)\\ \; & +\left(\mu +\delta \right)\left(\alpha_{3}+\alpha_{1}\right)\left(\varepsilon +\mu -\beta S^{*}\right)\varrho^{\alpha_{3}+\alpha_{1}}\cos\left(\vartheta \varrho +\frac{\pi \alpha_{3}}{2}\right)+\gamma \left(\mu +\delta \right)\left(\alpha_{3}+\alpha_{1}\right)\varrho^{\alpha_{3}+\alpha_{1}}\cos\left(\frac{\pi \alpha_{3}}{2}\right)\\ \; & +\alpha_{1}\left(\mu +\delta \right)\left(\mu^{2}+\varepsilon \mu -\beta S^{*}\varepsilon -\beta S^{*}\mu +\delta \varepsilon +\delta \mu -\beta S^{*}\delta \right)\varrho^{\alpha_{1}}\cos\left(\vartheta \varrho \right)+\alpha_{1}\gamma {\left(\mu +\delta \right)}^{2}\varrho^{\alpha_{1}}\\ \; & +\alpha_{2}\left(\mu +\delta \right)\left(\mu^{2}+\beta B^{*}\mu +\beta L^{*}\mu +\delta \mu +\beta B^{*}\delta +\beta L^{*}\delta \right)\varrho^{\alpha_{2}}\cos\left(\frac{\pi (\alpha_{2}-\alpha_{1})}{2}+\vartheta \varrho \right)\\ \; & +\alpha_{3}\left(\mu +\delta \right)\left(\mu^{2}+\varepsilon \mu +\beta B^{*}\varepsilon +\beta B^{*}\mu +\beta L^{*}\varepsilon +\beta L^{*}\mu -\beta S^{*}\mu \right)\varrho^{\alpha_{3}}\cos\left(\frac{\pi (\alpha_{3}-\alpha_{1})}{2}+\vartheta \varrho \right)\\ \; & +\alpha_{3}\gamma \mu \left(\mu +\delta \right)\varrho^{\alpha_{3}}\cos\left(\frac{\pi (\alpha_{3}-\alpha_{1})}{2}\right)+\mu \left(\alpha_{1}+\alpha_{2}+\alpha_{3}\right)\varrho^{\alpha_{1}+\alpha_{2}+\alpha_{3}}\cos\left(\frac{\pi (\alpha_{1}+\alpha_{2})}{2}+\vartheta \varrho \right)\\ \; & +\mu \left(\alpha_{1}+\alpha_{2}\right)\left(\mu +\delta \right)\varrho^{\alpha_{1}+\alpha_{2}}\cos\left(\frac{\pi (\alpha_{1}+\alpha_{2}-\alpha_{3})}{2}+\vartheta \varrho \right)\\ \; & +\mu \left(\alpha_{2}+\alpha_{3}\right)\left(\mu +\beta B^{*}+\beta L^{*}\right)\varrho^{\alpha_{2}+\alpha_{3}}\cos\left(\vartheta \varrho +\frac{\pi \alpha_{2}}{2}\right)\\ \; & +\mu \left(\alpha_{3}+\alpha_{1}\right)\left(\varepsilon +\mu -\beta S^{*}\right)\varrho^{\alpha_{3}+\alpha_{1}}\cos\left(\vartheta \varrho +\frac{\pi \alpha_{1}}{2}\right)+\mu \gamma \left(\alpha_{3}+\alpha_{1}\right)\varrho^{\alpha_{3}+\alpha_{1}}\cos\left(\frac{\pi \alpha_{1}}{2}\right)\\ \; & +\alpha_{1}\mu \left(\mu^{2}+\varepsilon \mu -\beta S^{*}\varepsilon -\beta S^{*}\mu +\delta \varepsilon +\delta \mu -\beta S^{*}\delta \right)\varrho^{\alpha_{1}}\cos\left(\frac{\pi (\alpha_{1}-\alpha_{3})}{2}+\vartheta \varrho \right)\\ \; & +\alpha_{1}\mu \gamma \left(\mu +\delta \right)\varrho^{\alpha_{1}}\cos\left(\frac{\pi (\alpha_{1}-\alpha_{3})}{2}\right)\\ \; & +\alpha_{2}\mu \left(\mu^{2}+\beta B^{*}\mu +\beta L^{*}\mu +\delta \mu +\beta B^{*}\delta +\beta L^{*}\delta \right)\varrho^{\alpha_{2}}\cos\left(\frac{\pi (\alpha_{2}-\alpha_{3})}{2}+\vartheta \varrho \right)\\ \; & +\alpha_{3}\mu \left(\mu^{2}+\varepsilon \mu +\beta B^{*}\varepsilon +\beta B^{*}\mu +\beta L^{*}\varepsilon +\beta L^{*}\mu -\beta S^{*}\mu \right)\varrho^{\alpha_{3}}\cos\left(\vartheta \varrho \right)+\alpha_{3}\gamma \mu^{2}\varrho^{\alpha_{3}}\\ \; & +\left(\mu^{2}+\delta \mu \right)\left(\alpha_{1}+\alpha_{2}+\alpha_{3}\right)\varrho^{\alpha_{1}+\alpha_{2}+\alpha_{3}}\cos\left(\frac{\pi (\alpha_{1}+\alpha_{2}+\alpha_{3})}{2}+\vartheta \varrho \right)\\ \; & +\left(\mu^{2}+\delta \mu \right)\left(\alpha_{1}+\alpha_{2}\right)\left(\mu +\delta \right)\varrho^{\alpha_{1}+\alpha_{2}}\cos\left(\frac{\pi (\alpha_{1}+\alpha_{2})}{2}+\vartheta \varrho \right)\\ \; & +\left(\mu^{2}+\delta \mu \right)\left(\alpha_{2}+\alpha_{3}\right)\left(\mu +\beta B^{*}+\beta L^{*}\right)\varrho^{\alpha_{2}+\alpha_{3}}\cos\left(\frac{\pi (\alpha_{2}+\alpha_{3})}{2}+\vartheta \varrho \right)\\ \; & +\left(\mu^{2}+\delta \mu \right)\left(\alpha_{3}+\alpha_{1}\right)\left(\varepsilon +\mu -\beta S^{*}\right)\varrho^{\alpha_{3}+\alpha_{1}}\cos\left(\frac{\pi (\alpha_{3}+\alpha_{1})}{2}+\vartheta \varrho \right)\\ \; & +\gamma \left(\mu^{2}+\delta \mu \right)\left(\alpha_{3}+\alpha_{1}\right)\varrho^{\alpha_{3}+\alpha_{1}}\cos\left(\frac{\pi (\alpha_{3}+\alpha_{1})}{2}\right)+\alpha_{1}\gamma \mu {\left(\mu +\delta \right)}^{2}\varrho^{\alpha_{1}}\cos\left(\frac{\pi \alpha_{1}}{2}\right)\\ \; & +\alpha_{1}\left(\mu^{2}+\delta \mu \right)\left(\mu^{2}+\varepsilon \mu -\beta S^{*}\varepsilon -\beta S^{*}\mu +\delta \varepsilon +\delta \mu -\beta S^{*}\delta \right)\varrho^{\alpha_{1}}\cos\left(\vartheta \varrho +\frac{\pi \alpha_{1}}{2}\right)\\ \; & +\alpha_{2}\left(\mu^{2}+\delta \mu \right)\left(\mu^{2}+\beta B^{*}\mu +\beta L^{*}\mu +\delta \mu +\beta B^{*}\delta +\beta L^{*}\delta \right)\varrho^{\alpha_{2}}\cos\left(\vartheta \varrho +\frac{\pi \alpha_{2}}{2}\right)\\ \; & +\alpha_{3}\left(\mu^{2}+\delta \mu \right)\left(\mu^{2}+\varepsilon \mu +\beta B^{*}\varepsilon +\beta B^{*}\mu +\beta L^{*}\varepsilon +\beta L^{*}\mu -\beta S^{*}\mu \right)\varrho^{\alpha_{3}}\cos\left(\vartheta \varrho +\frac{\pi \alpha_{2}}{2}\right)\\ \; & +\alpha_{3}\gamma \mu \left(\mu^{2}+\delta \mu \right)\varrho^{\alpha_{3}}\cos\left(\frac{\pi \alpha_{3}}{2}\right). \end{array}$$

**Theorem** **1.** *Let $W_{1}$ and $W_{2}\left(\varrho ,\vartheta \right)$ be defined as in Equations (22) and (23), respectively. And let the strictly positive constants $\overset{´}{\vartheta }$ and $\overset{´}{\varrho }$ be defined respectively by*

(24) $$\overset{´}{\vartheta }=\min\left\{\vartheta \in R_{+};\;▽\left(\varrho i;\vartheta ,0\right)=0\;\mathrm{holds}\;\mathrm{for}\;\mathrm{some}\;\varrho \in R_{+}\right\}$$

*and*

(25) $$\overset{´}{\varrho }=\min\left\{\varrho \in R_{+};\;▽\left(\varrho i;\overset{´}{\vartheta },0\right)=0\right\},$$

where ▽(ξ;ϑ,υ) is defined as in Equation (16). Suppose that the conditions $W_{1}<0$ and $W_{2}\left(\overset{´}{\varrho },\overset{´}{\vartheta }\right)>0$ are satisfied. For DFSLB (3) with $\tau_{2}=0$, the endemic equilibrium $\left(S^{*},L^{*},B^{*}\right)$ is asymptotically stable whenever $0⩽\tau_{1}<\overset{´}{\vartheta }$. Moreover, at $\tau_{1}=\overset{´}{\vartheta }$, DFSLB (3) undergoes a Hopf bifurcation, giving rise to periodic solutions that branch from the same endemic equilibrium $\left(S^{*},L^{*},B^{*}\right)$.

**Remark** **3.** *Before presenting the proof of Theorem 1, it is worth noting that the main idea used in the proof of Theorem 1 has been widely employed in the literature; see, for example, [23,25,26,28,33,46]. In this approach, it is crucial to verify the existence of a positive constant $\overset{´}{\vartheta }$ such that the equation $▽(i\varrho ;\overset{˘}{\vartheta },0)=0$ admits a positive solution, denoted by $\overset{´}{\varrho }$. Moreover, it is required that ${\left.\frac{d\mu }{d\vartheta }\right|}_{\vartheta =\overset{´}{\vartheta },\;\mu =\overset{´}{\varrho }}>0$, where μ is implicitly defined as a function of ϑ by the equation ▽(μ;ϑ,0)=0 in a neighborhood of the point $\left(\overset{´}{\vartheta },\overset{´}{\varrho }i\right)$. It is straightforward to observe that the same argument will also employed to establish the Hopf bifurcation results stated in Theorem 2 in this paper.*

**Proof.**  With the aid of Equation (18), we obtain

(26) $$\begin{array}{cc} ▽(\varrho i;\vartheta ,0)= & \varrho^{\alpha_{1}+\alpha_{2}+\alpha_{3}}e^{\frac{\pi (\alpha_{1}+\alpha_{2}+\alpha_{3})i}{2}}+\left(\mu +\delta \right)\varrho^{\alpha_{1}+\alpha_{2}}e^{\frac{\pi (\alpha_{1}+\alpha_{2})i}{2}}\\ \; & +\left(\mu +\beta B^{*}+\beta L^{*}\right)\varrho^{\alpha_{2}+\alpha_{3}}e^{\frac{\pi (\alpha_{2}+\alpha_{3})i}{2}}\\ \; & +\left(\varepsilon +\mu -\beta S^{*}+\gamma e^{-\vartheta \varrho i}\right)\varrho^{\alpha_{3}+\alpha_{1}}e^{\frac{\pi (\alpha_{3}+\alpha_{1})i}{2}}+(\mu^{2}+\varepsilon \mu -\beta S^{*}\varepsilon -\beta S^{*}\mu \\ \; & +\gamma \mu e^{-\vartheta \varrho i}+\delta \varepsilon +\delta \mu -\beta S^{*}\delta +\delta \gamma e^{-\varrho \vartheta i})\varrho^{\alpha_{1}}e^{\frac{\pi \alpha_{1}i}{2}}\\ \; & +\left(\mu^{2}+\beta B^{*}\mu +\beta L^{*}\mu +\delta \mu +\beta B^{*}\delta +\beta L^{*}\delta \right)\varrho^{\alpha_{2}}e^{\frac{\pi \alpha_{2}i}{2}}\\ \; & +\left(\mu^{2}+\varepsilon \mu +\beta B^{*}\varepsilon +\beta B^{*}\mu +\beta L^{*}\varepsilon +\beta L^{*}\mu -\beta S^{*}\mu +\gamma \mu e^{-\vartheta \varrho i}\right)\varrho^{\alpha_{3}}e^{\frac{\pi \alpha_{3}i}{2}}\\ \; & +\mu^{3}+\varepsilon \mu^{2}+B^{*}\beta \mu^{2}+L^{*}\beta \mu^{2}-S^{*}\beta \mu^{2}+B^{*}\beta \varepsilon \mu +L^{*}\beta \varepsilon \mu -S^{*}\beta \varepsilon \mu \\ \; & +\gamma \mu^{2}e^{-\vartheta \varrho i}+\delta \mu^{2}+\delta \varepsilon \mu +L^{*}\beta \delta \mu -S^{*}\beta \delta \mu \\ \; & +B^{*}\beta \delta \mu +\delta \gamma \mu e^{-\varrho \vartheta i},\;\forall \varrho ,\vartheta \in \left[0,+\infty \right). \end{array}$$

By routine calculations, we can rewrite ▽(ϱi;ϑ,0) defined in Equation (26) as

(27) $$▽\left(\varrho i;\vartheta ,0\right)=H_{01}\left(\varrho \right)+H_{02}\left(\varrho \right)e^{-\varrho \vartheta i},$$

where $H_{01}\left(\varrho \right)$ and $H_{02}\left(\varrho \right)$ are, respectively, defined as

(28) $$\begin{array}{cc} H_{01}\left(\varrho \right)= & \varrho^{\alpha_{1}+\alpha_{2}+\alpha_{3}}e^{\frac{\pi (\alpha_{1}+\alpha_{2}+\alpha_{3})i}{2}}+\left(\mu +\delta \right)\varrho^{\alpha_{1}+\alpha_{2}}e^{\frac{\pi (\alpha_{1}+\alpha_{2})i}{2}}\\ \; & +\left(\mu +\beta B^{*}+\beta L^{*}\right)\varrho^{\alpha_{2}+\alpha_{3}}e^{\frac{\pi (\alpha_{2}+\alpha_{3})i}{2}}+\left(\varepsilon +\mu -\beta S^{*}\right)\varrho^{\alpha_{3}+\alpha_{1}}e^{\frac{\pi (\alpha_{3}+\alpha_{1})i}{2}}\\ \; & +\left(\mu^{2}+\varepsilon \mu -\beta S^{*}\varepsilon -\beta S^{*}\mu +\delta \varepsilon +\delta \mu -\beta S^{*}\delta \right)\varrho^{\alpha_{1}}e^{\frac{\pi \alpha_{1}i}{2}}\\ \; & +\left(\mu^{2}+\beta B^{*}\mu +\beta L^{*}\mu +\delta \mu +\beta B^{*}\delta +\beta L^{*}\delta \right)\varrho^{\alpha_{2}}e^{\frac{\pi \alpha_{2}i}{2}}\\ \; & +\left(\mu^{2}+\varepsilon \mu +\beta B^{*}\varepsilon +\beta B^{*}\mu +\beta L^{*}\varepsilon +\beta L^{*}\mu -\beta S^{*}\mu \right)\varrho^{\alpha_{3}}e^{\frac{\pi \alpha_{3}i}{2}}\\ \; & +\mu^{3}+\varepsilon \mu^{2}+B^{*}\beta \mu^{2}+L^{*}\beta \mu^{2}-S^{*}\beta \mu^{2}+B^{*}\beta \varepsilon \mu +L^{*}\beta \varepsilon \mu -S^{*}\beta \varepsilon \mu \\ \; & +\delta \mu^{2}+\delta \varepsilon \mu +L^{*}\beta \delta \mu -S^{*}\beta \delta \mu +B^{*}\beta \delta \mu \end{array}$$

and

(29) $$H_{02}\left(\varrho \right)=\gamma \left(\varrho^{\alpha_{3}+\alpha_{1}}e^{\frac{\pi (\alpha_{3}+\alpha_{1})i}{2}}+\mu \varrho^{\alpha_{1}}e^{\frac{\pi \alpha_{1}i}{2}}+\delta \varrho^{\alpha_{1}}e^{\frac{\pi \alpha_{1}i}{2}}+\mu \varrho^{\alpha_{3}}e^{\frac{\pi \alpha_{3}i}{2}}+\mu^{2}+\delta \mu \right).$$

With the aid of Equation (27), we conclude that a necessary and sufficient condition for $▽(\overset{´}{\varrho }i;\overset{´}{\vartheta },0)=0$ is given by

(30) $$H_{01}\left(\overset{´}{\varrho }\right)+H_{02}\left(\overset{´}{\varrho }\right)e^{-\overset{´}{\varrho }\overset{´}{\vartheta }i}=0,$$

which necessitates the following condition

(31) $$\Omega_{1}\left(\overset{´}{\varrho }\right)=|H_{01}\left(\overset{´}{\varrho }\right){|}^{2}-{\left|H_{02}\left(\overset{´}{\varrho }\right)\right|}^{2}=0.$$

Motivated by this, and following straightforward calculations, we have

(32) $$\Omega_{1}\left(\varrho \right)=|H_{01}{\left(\varrho \right)|}^{2}-{\left|H_{02}\left(\varrho \right)\right|}^{2}=\varrho^{2(\alpha_{1}+\alpha_{2}+\alpha_{3})}+\sum_{j=1}^{5}H_{03j}\left(\varrho \right)+W_{1},$$

in which, $W_{1}$, $H_{031}\left(\varrho \right)$, $H_{032}\left(\varrho \right)$, $H_{033}\left(\varrho \right)$, $H_{034}\left(\varrho \right)$, and $H_{035}\left(\varrho \right)$ are, respectively, defined in Equation (22) and by

(33) $$\begin{array}{cc} H_{031}\left(\varrho \right)=\; & 2(\mu^{3}+\varepsilon \mu^{2}+B^{*}\beta \mu^{2}+L^{*}\beta \mu^{2}-S^{*}\beta \mu^{2}+B^{*}\beta \varepsilon \mu +L^{*}\beta \varepsilon \mu -S^{*}\beta \varepsilon \mu \\ \; & +\delta \mu^{2}+\delta \varepsilon \mu +L^{*}\beta \delta \mu -S^{*}\beta \delta \mu +B^{*}\beta \delta \mu )\left(\mu^{2}+\varepsilon \mu -\beta S^{*}\varepsilon -\beta S^{*}\mu +\delta \varepsilon +\delta \mu -\beta S^{*}\delta \right)\varrho^{\alpha_{1}}\cos\left(\frac{\pi \alpha_{1}}{2}\right)\\ \; & +2(\mu^{3}+\varepsilon \mu^{2}+B^{*}\beta \mu^{2}+L^{*}\beta \mu^{2}-S^{*}\beta \mu^{2}+B^{*}\beta \varepsilon \mu +L^{*}\beta \varepsilon \mu -S^{*}\beta \varepsilon \mu \\ \; & +\delta \mu^{2}+\delta \varepsilon \mu +L^{*}\beta \delta \mu -S^{*}\beta \delta \mu +B^{*}\beta \delta \mu )\left(\mu^{2}+\beta B^{*}\mu +\beta L^{*}\mu +\delta \mu +\beta B^{*}\delta +\beta L^{*}\delta \right)\varrho^{\alpha_{2}}\cos\left(\frac{\pi \alpha_{2}}{2}\right)\\ \; & +2(\mu^{3}+\varepsilon \mu^{2}+B^{*}\beta \mu^{2}+L^{*}\beta \mu^{2}-S^{*}\beta \mu^{2}+B^{*}\beta \varepsilon \mu +L^{*}\beta \varepsilon \mu -S^{*}\beta \varepsilon \mu +\delta \mu^{2}+\delta \varepsilon \mu \\ \; & +L^{*}\beta \delta \mu -S^{*}\beta \delta \mu +B^{*}\beta \delta \mu )\left(\mu^{2}+\varepsilon \mu +\beta B^{*}\varepsilon +\beta B^{*}\mu +\beta L^{*}\varepsilon +\beta L^{*}\mu -\beta S^{*}\mu \right)\varrho^{\alpha_{3}}\cos\left(\frac{\pi \alpha_{3}}{2}\right)\\ \; & -2\gamma^{2}\mu {\left(\mu +\delta \right)}^{2}\varrho^{\alpha_{1}}\cos\left(\frac{\pi \alpha_{1}}{2}\right)-2\gamma^{2}\mu \left(\mu^{2}+\delta \mu \right)\varrho^{\alpha_{3}}\cos\left(\frac{\pi \alpha_{3}}{2}\right), \end{array}$$

(34) $$\begin{array}{cc} H_{032}\left(\varrho \right)= & 2(\mu^{3}+\varepsilon \mu^{2}+B^{*}\beta \mu^{2}+L^{*}\beta \mu^{2}-S^{*}\beta \mu^{2}+B^{*}\beta \varepsilon \mu +L^{*}\beta \varepsilon \mu -S^{*}\beta \varepsilon \mu \\ \; & +\delta \mu^{2}+\delta \varepsilon \mu +L^{*}\beta \delta \mu -S^{*}\beta \delta \mu +B^{*}\beta \delta \mu )\left(\mu +\delta \right)\varrho^{\alpha_{1}+\alpha_{2}}\cos\left(\frac{\pi (\alpha_{1}+\alpha_{2})}{2}\right)\\ \; & +2(\mu^{3}+\varepsilon \mu^{2}+B^{*}\beta \mu^{2}+L^{*}\beta \mu^{2}-S^{*}\beta \mu^{2}+B^{*}\beta \varepsilon \mu +L^{*}\beta \varepsilon \mu -S^{*}\beta \varepsilon \mu \\ \; & +\delta \mu^{2}+\delta \varepsilon \mu +L^{*}\beta \delta \mu -S^{*}\beta \delta \mu +B^{*}\beta \delta \mu )\left(\mu +\beta B^{*}+\beta L^{*}\right)\varrho^{\alpha_{2}+\alpha_{3}}\cos\left(\frac{\pi (\alpha_{2}+\alpha_{3})}{2}\right)\\ \; & +2(\mu^{3}+\varepsilon \mu^{2}+B^{*}\beta \mu^{2}+L^{*}\beta \mu^{2}-S^{*}\beta \mu^{2}+B^{*}\beta \varepsilon \mu +L^{*}\beta \varepsilon \mu -S^{*}\beta \varepsilon \mu \\ \; & +\delta \mu^{2}+\delta \varepsilon \mu +L^{*}\beta \delta \mu -S^{*}\beta \delta \mu +B^{*}\beta \delta \mu )\left(\varepsilon +\mu -\beta S^{*}\right)\varrho^{\alpha_{3}+\alpha_{1}}\cos\left(\frac{\pi (\alpha_{3}+\alpha_{1})}{2}\right)\\ \; & +{\left(\mu^{2}+\varepsilon \mu -\beta S^{*}\varepsilon -\beta S^{*}\mu +\delta \varepsilon +\delta \mu -\beta S^{*}\delta \right)}^{2}\varrho^{2\alpha_{1}}+{\left(\mu^{2}+\beta B^{*}\mu +\beta L^{*}\mu +\delta \mu +\beta B^{*}\delta +\beta L^{*}\delta \right)}^{2}\varrho^{2\alpha_{2}}\\ \; & +{\left(\mu^{2}+\varepsilon \mu +\beta B^{*}\varepsilon +\beta B^{*}\mu +\beta L^{*}\varepsilon +\beta L^{*}\mu -\beta S^{*}\mu \right)}^{2}\varrho^{2\alpha_{3}}+2(\mu^{2}+\varepsilon \mu -\beta S^{*}\varepsilon -\beta S^{*}\mu +\delta \varepsilon \\ \; & +\delta \mu -\beta S^{*}\delta )\left(\mu^{2}+\beta B^{*}\mu +\beta L^{*}\mu +\delta \mu +\beta B^{*}\delta +\beta L^{*}\delta \right)\varrho^{\alpha_{1}+\alpha_{2}}\cos\left(\frac{\pi (\alpha_{2}-\alpha_{1})}{2}\right)+2(\mu^{2}+\varepsilon \mu -\beta S^{*}\varepsilon \\ \; & -\beta S^{*}\mu +\delta \varepsilon +\delta \mu -\beta S^{*}\delta )\left(\mu^{2}+\varepsilon \mu +\beta B^{*}\varepsilon +\beta B^{*}\mu +\beta L^{*}\varepsilon +\beta L^{*}\mu -\beta S^{*}\mu \right)\varrho^{\alpha_{3}+\alpha_{1}}\cos\left(\frac{\pi (\alpha_{1}-\alpha_{3})}{2}\right)\\ \; & +2\left(\mu^{2}+\beta B^{*}\mu +\beta L^{*}\mu +\delta \mu +\beta B^{*}\delta +\beta L^{*}\delta \right)(\mu^{2}+\varepsilon \mu +\beta B^{*}\varepsilon +\beta B^{*}\mu +\beta L^{*}\varepsilon \\ \; & +\beta L^{*}\mu -\beta S^{*}\mu )\varrho^{\alpha_{2}+\alpha_{3}}\cos\left(\frac{\pi (\alpha_{3}-\alpha_{2})}{2}\right)-2\gamma^{2}\left(\mu^{2}+\delta \mu \right)\varrho^{\alpha_{3}+\alpha_{1}}\cos\left(\frac{\pi (\alpha_{3}+\alpha_{1})}{2}\right)-\gamma^{2}{\left(\mu +\delta \right)}^{2}\varrho^{2\alpha_{1}}\\ \; & -\gamma^{2}\mu^{2}\varrho^{2\alpha_{3}}-2\gamma^{2}\mu \left(\mu +\delta \right)\varrho^{\alpha_{3}+\alpha_{1}}\cos\left(\frac{\pi (\alpha_{1}-\alpha_{3})}{2}\right), \end{array}$$

(35) $$\begin{array}{cc} H_{033}\left(\varrho \right)= & 2(\mu^{3}+\varepsilon \mu^{2}+B^{*}\beta \mu^{2}+L^{*}\beta \mu^{2}-S^{*}\beta \mu^{2}+B^{*}\beta \varepsilon \mu +L^{*}\beta \varepsilon \mu -S^{*}\beta \varepsilon \mu \\ \; & +\delta \mu^{2}+\delta \varepsilon \mu +L^{*}\beta \delta \mu -S^{*}\beta \delta \mu +B^{*}\beta \delta \mu )\varrho^{\alpha_{1}+\alpha_{2}+\alpha_{3}}\cos\left(\frac{\pi (\alpha_{1}+\alpha_{2}+\alpha_{3})}{2}\right)\\ \; & +2\left(\mu +\delta \right)\left(\mu^{2}+\varepsilon \mu -\beta S^{*}\varepsilon -\beta S^{*}\mu +\delta \varepsilon +\delta \mu -\beta S^{*}\delta \right)\varrho^{2\alpha_{1}+\alpha_{2}}\cos\left(\frac{\pi \alpha_{2}}{2}\right)\\ \; & +2\left(\mu +\delta \right)\left(\mu^{2}+\beta B^{*}\mu +\beta L^{*}\mu +\delta \mu +\beta B^{*}\delta +\beta L^{*}\delta \right)\varrho^{\alpha_{1}+2\alpha_{2}}\cos\left(\frac{\pi \alpha_{1}}{2}\right)\\ \; & +2\left(\mu +\delta \right)\left(\mu^{2}+\varepsilon \mu +\beta B^{*}\varepsilon +\beta B^{*}\mu +\beta L^{*}\varepsilon +\beta L^{*}\mu -\beta S^{*}\mu \right)\varrho^{\alpha_{1}+\alpha_{2}+\alpha_{3}}\cos\left(\frac{\pi (\alpha_{1}+\alpha_{2}-\alpha_{3})}{2}\right)\\ \; & +2\left(\mu +\beta B^{*}+\beta L^{*}\right)\left(\mu^{2}+\varepsilon \mu -\beta S^{*}\varepsilon -\beta S^{*}\mu +\delta \varepsilon +\delta \mu -\beta S^{*}\delta \right)\varrho^{\alpha_{1}+\alpha_{2}+\alpha_{3}}\cos\left(\frac{\pi (\alpha_{2}+\alpha_{3}-\alpha_{1})}{2}\right)\\ \; & +2\left(\mu +\beta B^{*}+\beta L^{*}\right)\left(\mu^{2}+\beta B^{*}\mu +\beta L^{*}\mu +\delta \mu +\beta B^{*}\delta +\beta L^{*}\delta \right)\varrho^{2\alpha_{2}+\alpha_{3}}\cos\left(\frac{\pi \alpha_{3}}{2}\right)\\ \; & +2\left(\mu +\beta B^{*}+\beta L^{*}\right)\left(\mu^{2}+\varepsilon \mu +\beta B^{*}\varepsilon +\beta B^{*}\mu +\beta L^{*}\varepsilon +\beta L^{*}\mu -\beta S^{*}\mu \right)\varrho^{\alpha_{2}+2\alpha_{3}}\cos\left(\frac{\pi \alpha_{2}}{2}\right)\\ \; & +2\left(\varepsilon +\mu -\beta S^{*}\right)\left(\mu^{2}+\varepsilon \mu -\beta S^{*}\varepsilon -\beta S^{*}\mu +\delta \varepsilon +\delta \mu -\beta S^{*}\delta \right)\varrho^{2\alpha_{1}+\alpha_{3}}\cos\left(\frac{\pi \alpha_{3}}{2}\right)\\ \; & +2\left(\varepsilon +\mu -\beta S^{*}\right)\left(\mu^{2}+\beta B^{*}\mu +\beta L^{*}\mu +\delta \mu +\beta B^{*}\delta +\beta L^{*}\delta \right)\varrho^{\alpha_{1}+\alpha_{2}+\alpha_{3}}\cos\left(\frac{\pi (\alpha_{1}-\alpha_{2}+\alpha_{3})}{2}\right)\\ \; & +2\left(\varepsilon +\mu -\beta S^{*}\right)\left(\mu^{2}+\varepsilon \mu +\beta B^{*}\varepsilon +\beta B^{*}\mu +\beta L^{*}\varepsilon +\beta L^{*}\mu -\beta S^{*}\mu \right)\varrho^{\alpha_{1}+2\alpha_{3}}\cos\left(\frac{\pi \alpha_{1}}{2}\right)\\ \; & -2\gamma^{2}\left(\mu \varrho^{\alpha_{3}+2\alpha_{1}}\cos\left(\frac{\pi \alpha_{3}}{2}\right)+\delta \varrho^{\alpha_{3}+2\alpha_{1}}\cos\left(\frac{\pi \alpha_{3}}{2}\right)+\mu \varrho^{2\alpha_{3}+\alpha_{1}}\cos\left(\frac{\pi \alpha_{1}}{2}\right)\right), \end{array}$$

(36) $$\begin{array}{cc} H_{034}\left(\varrho \right)= & {\left(\mu +\delta \right)}^{2}\varrho^{2\alpha_{1}+2\alpha_{2}}+{\left(\mu +\beta B^{*}+\beta L^{*}\right)}^{2}\varrho^{2\alpha_{2}+2\alpha_{3}}+{\left(\varepsilon +\mu -\beta S^{*}\right)}^{2}\varrho^{2\alpha_{3}+2\alpha_{1}}\\ \; & +2\left(\mu^{2}+\varepsilon \mu -\beta S^{*}\varepsilon -\beta S^{*}\mu +\delta \varepsilon +\delta \mu -\beta S^{*}\delta \right)\varrho^{2\alpha_{1}+\alpha_{2}+\alpha_{3}}\cos\left(\frac{\pi (\alpha_{2}+\alpha_{3})}{2}\right)\\ \; & +2\left(\mu^{2}+\beta B^{*}\mu +\beta L^{*}\mu +\delta \mu +\beta B^{*}\delta +\beta L^{*}\delta \right)\varrho^{\alpha_{1}+2\alpha_{2}+\alpha_{3}}\cos\left(\frac{\pi (\alpha_{3}+\alpha_{1})}{2}\right)\\ \; & +2\left(\mu^{2}+\varepsilon \mu +\beta B^{*}\varepsilon +\beta B^{*}\mu +\beta L^{*}\varepsilon +\beta L^{*}\mu -\beta S^{*}\mu \right)\varrho^{\alpha_{1}+\alpha_{2}+2\alpha_{3}}\cos\left(\frac{\pi (\alpha_{1}+\alpha_{2})}{2}\right)\\ \; & +2\left(\mu +\delta \right)(\left(\mu +\beta B^{*}+\beta L^{*}\right)\varrho^{\alpha_{1}+2\alpha_{2}+\alpha_{3}}\cos\left(\frac{\pi (\alpha_{1}-\alpha_{3})}{2}\right)+\left(\varepsilon +\mu -\beta S^{*}\right)\varrho^{2\alpha_{1}+\alpha_{2}+\alpha_{3}}\cos\left(\frac{\pi (\alpha_{3}-\alpha_{2})}{2}\right))\\ \; & +2\left(\mu +\beta B^{*}+\beta L^{*}\right)\left(\varepsilon +\mu -\beta S^{*}\right)\varrho^{\alpha_{1}+\alpha_{2}+2\alpha_{3}}\cos\left(\frac{\pi (\alpha_{2}-\alpha_{1})}{2}\right)-\gamma^{2}\varrho^{2\alpha_{3}+2\alpha_{1}}, \end{array}$$

and by

(37) $$\begin{array}{cc} H_{035}\left(\varrho \right)= & 2\left(\mu +\delta \right)\varrho^{2\alpha_{1}+2\alpha_{2}+\alpha_{3}}\cos\left(\frac{\pi \alpha_{3}}{2}\right)+2\left(\mu +\beta B^{*}+\beta L^{*}\right)\varrho^{\alpha_{1}+2\alpha_{2}+2\alpha_{3}}\cos\left(\frac{\pi \alpha_{1}}{2}\right)\\ \; & +2\left(\varepsilon +\mu -\beta S^{*}\right)\varrho^{2\alpha_{1}+\alpha_{2}+2\alpha_{3}}\cos\left(\frac{\pi \alpha_{2}}{2}\right). \end{array}$$

By virtue of Equations (33)–(37), it follows that

(38) $$H_{03j}\left(0\right)=0,\;j=1,2,3,4,5.$$

Together with Equation (32), this immediately implies $\Omega_{1}\left(0\right)=W_{1}<0$, where the constant $W_{1}$ is defined as in Equation (22). Moreover, one readily verifies that

(39) $$\lim_{\varrho \to +\infty }\Omega_{1}\left(\varrho \right)=+\infty .$$

By the limit properties of functions, there exists ${\hat{\varrho }}_{1}\in \left(0,+\infty \right)$ such that $\Omega_{1}\left({\hat{\varrho }}_{1}\right)>0$. Applying the intermediate value theorem to the continuous function $\Omega_{1}\left(\varrho \right)$ then yields a constant $\varrho_{1}\in \left(0,{\hat{\varrho }}_{1}\right)$ satisfying $\Omega_{1}\left(\varrho_{1}\right)=0$. Consider now the following algebraic equation in the unknown $\vartheta_{1}\in \left(0,+\infty \right)$:

(40) $$▽(\varrho_{1}i;\vartheta_{1},0)=0,$$

where ▽(ϱi;ϑ,0) is given as in Equation (26). With the aid of Equation (27), Equation (40) can be recast into the following equivalent form

(41) $$\begin{array}{cc} \cos\left(\vartheta_{1}\varrho_{1}\right)+i\sin\left(\vartheta_{1}\varrho_{1}\right)= & e^{\vartheta_{1}\varrho_{1}i}=-\frac{H_{02}\left(\varrho_{1}\right)}{H_{01}\left(\varrho_{1}\right)}=-\frac{\bar{H_{01}\left(\varrho_{1}\right)}H_{02}\left(\varrho_{1}\right)}{|H_{01}\left(\varrho_{1}\right){|}^{2}}\\ = & -\frac{\bar{H_{01}\left(\varrho_{1}\right)}H_{02}\left(\varrho_{1}\right)}{|H_{02}\left(\varrho_{1}\right){|}^{2}}=-\frac{H_{04R}\left(\varrho_{1}\right)}{|H_{02}\left(\varrho_{1}\right){|}^{2}}-i\frac{H_{04I}\left(\varrho_{1}\right)}{|H_{02}\left(\varrho_{1}\right){|}^{2}}, \end{array}$$

in which $|H_{02}{\left(\varrho \right)|}^{2}$ can be simplified to

(42) $$\begin{array}{cc} |H_{02}{\left(\varrho \right)|}^{2}= & \gamma^{2}(\varrho^{2\alpha_{3}+2\alpha_{1}}+2\left(\mu +\delta \right)\varrho^{\alpha_{3}+2\alpha_{1}}\cos\left(\frac{\pi \alpha_{3}}{2}\right)+2\mu \varrho^{2\alpha_{3}+\alpha_{1}}\cos\left(\frac{\pi \alpha_{1}}{2}\right)+2\mu \left(\mu +\delta \right)\varrho^{\alpha_{3}+\alpha_{1}}\cos\left(\frac{\pi (\alpha_{3}+\alpha_{1})}{2}\right)\\ \; & \;+{\left(\mu +\delta \right)}^{2}\varrho^{2\alpha_{1}}+\mu^{2}\varrho^{2\alpha_{3}}+2\mu \left(\mu +\delta \right)\varrho^{\alpha_{3}+\alpha_{1}}\cos\left(\frac{\pi (\alpha_{1}-\alpha_{3})}{2}\right)\\ \; & \;+2\mu {\left(\mu +\delta \right)}^{2}\varrho^{\alpha_{1}}\cos\left(\frac{\pi \alpha_{1}}{2}\right)+2\mu^{2}\left(\mu +\delta \right)\varrho^{\alpha_{3}}\cos\left(\frac{\pi \alpha_{3}}{2}\right)+\mu^{2}{\left(\mu +\delta \right)}^{2}). \end{array}$$

In Equation (41), $H_{04R}\left(\varrho \right)$ and $H_{04I}\left(\varrho \right)$ are given respectively by

(43) $$\begin{array}{cc} H_{04R}\left(\varrho \right)= & \gamma \varrho^{2\alpha_{1}+\alpha_{2}+2\alpha_{3}}\cos\left(\frac{\pi \alpha_{2}}{2}\right)+\gamma \sum_{j=1}^{4}H_{04Rj}\left(\varrho \right)+\gamma (\mu^{3}+\varepsilon \mu^{2}+B^{*}\beta \mu^{2}+L^{*}\beta \mu^{2}-S^{*}\beta \mu^{2}\\ \; & +B^{*}\beta \varepsilon \mu +L^{*}\beta \varepsilon \mu -S^{*}\beta \varepsilon \mu +\delta \mu^{2}+\delta \varepsilon \mu +L^{*}\beta \delta \mu -S^{*}\beta \delta \mu +B^{*}\beta \delta \mu )\left(\mu^{2}+\delta \mu \right) \end{array}$$

and

(44) $$H_{04I}\left(\varrho \right)=-\gamma \varrho^{2\alpha_{1}+\alpha_{2}+2\alpha_{3}}\sin\left(\frac{\pi \alpha_{2}}{2}\right)+\gamma \sum_{j=1}^{4}H_{04Ij}\left(\varrho \right).$$

In Equations (43) and (44), the terms $H_{04R1}\left(\varrho \right)$, $H_{04I1}\left(\varrho \right)$, $H_{04R2}\left(\varrho \right)$, $H_{04I2}\left(\varrho \right)$, $H_{04R3}\left(\varrho \right)$, $H_{04I3}\left(\varrho \right)$, $H_{04R4}\left(\varrho \right)$, and $H_{04I4}\left(\varrho \right)$ are given respectively by

(45) $$\begin{array}{cc} H_{04R1}\left(\varrho \right)=\; & \left(\mu^{2}+\delta \mu \right)\left(\mu^{2}+\varepsilon \mu -\beta S^{*}\varepsilon -\beta S^{*}\mu +\delta \varepsilon +\delta \mu -\beta S^{*}\delta \right)\varrho^{\alpha_{1}}\cos\left(\frac{\pi \alpha_{1}}{2}\right)\\ \; & +\left(\mu^{2}+\delta \mu \right)\left(\mu^{2}+\beta B^{*}\mu +\beta L^{*}\mu +\delta \mu +\beta B^{*}\delta +\beta L^{*}\delta \right)\varrho^{\alpha_{2}}\cos\left(\frac{\pi \alpha_{2}}{2}\right)\\ \; & +\left(\mu^{2}+\delta \mu \right)\left(\mu^{2}+\varepsilon \mu +\beta B^{*}\varepsilon +\beta B^{*}\mu +\beta L^{*}\varepsilon +\beta L^{*}\mu -\beta S^{*}\mu \right)\varrho^{\alpha_{3}}\cos\left(\frac{\pi \alpha_{3}}{2}\right)\\ \; & +(\mu^{3}+\varepsilon \mu^{2}+B^{*}\beta \mu^{2}+L^{*}\beta \mu^{2}-S^{*}\beta \mu^{2}+B^{*}\beta \varepsilon \mu +L^{*}\beta \varepsilon \mu -S^{*}\beta \varepsilon \mu +\delta \mu^{2}+\delta \varepsilon \mu +L^{*}\beta \delta \mu -S^{*}\beta \delta \mu \\ \; & +B^{*}\beta \delta \mu )\left(\mu +\delta \right)\varrho^{\alpha_{1}}\cos\left(\frac{\pi \alpha_{1}}{2}\right)+\mu (\mu^{3}+\varepsilon \mu^{2}+B^{*}\beta \mu^{2}+L^{*}\beta \mu^{2}-S^{*}\beta \mu^{2}+B^{*}\beta \varepsilon \mu +L^{*}\beta \varepsilon \mu -S^{*}\beta \varepsilon \mu \\ \; & +\delta \mu^{2}+\delta \varepsilon \mu +L^{*}\beta \delta \mu -S^{*}\beta \delta \mu +B^{*}\beta \delta \mu )\varrho^{\alpha_{3}}\cos\left(\frac{\pi \alpha_{3}}{2}\right), \end{array}$$

(46) $$\begin{array}{cc} H_{04I1}\left(\varrho \right)=\; & -\left(\mu^{2}+\delta \mu \right)\left(\mu^{2}+\varepsilon \mu -\beta S^{*}\varepsilon -\beta S^{*}\mu +\delta \varepsilon +\delta \mu -\beta S^{*}\delta \right)\varrho^{\alpha_{1}}\sin\left(\frac{\pi \alpha_{1}}{2}\right)\\ \; & -\left(\mu^{2}+\delta \mu \right)\left(\mu^{2}+\beta B^{*}\mu +\beta L^{*}\mu +\delta \mu +\beta B^{*}\delta +\beta L^{*}\delta \right)\varrho^{\alpha_{2}}\sin\left(\frac{\pi \alpha_{2}}{2}\right)\\ \; & -\left(\mu^{2}+\delta \mu \right)\left(\mu^{2}+\varepsilon \mu +\beta B^{*}\varepsilon +\beta B^{*}\mu +\beta L^{*}\varepsilon +\beta L^{*}\mu -\beta S^{*}\mu \right)\varrho^{\alpha_{3}}\sin\left(\frac{\pi \alpha_{3}}{2}\right)\\ \; & +(\mu^{3}+\varepsilon \mu^{2}+B^{*}\beta \mu^{2}+L^{*}\beta \mu^{2}-S^{*}\beta \mu^{2}+B^{*}\beta \varepsilon \mu +L^{*}\beta \varepsilon \mu -S^{*}\beta \varepsilon \mu +\delta \mu^{2}+\delta \varepsilon \mu +L^{*}\beta \delta \mu -S^{*}\beta \delta \mu \\ \; & +B^{*}\beta \delta \mu )\left(\mu +\delta \right)\varrho^{\alpha_{1}}\sin\left(\frac{\pi \alpha_{1}}{2}\right)+\mu (\mu^{3}+\varepsilon \mu^{2}+B^{*}\beta \mu^{2}+L^{*}\beta \mu^{2}-S^{*}\beta \mu^{2}+B^{*}\beta \varepsilon \mu +L^{*}\beta \varepsilon \mu -S^{*}\beta \varepsilon \mu \\ \; & +\delta \mu^{2}+\delta \varepsilon \mu +L^{*}\beta \delta \mu -S^{*}\beta \delta \mu +B^{*}\beta \delta \mu )\varrho^{\alpha_{3}}\sin\left(\frac{\pi \alpha_{3}}{2}\right), \end{array}$$

(47) $$\begin{array}{cc} H_{04R2}\left(\varrho \right)= & \left(\mu +\delta \right)\left(\mu^{2}+\delta \mu \right)\varrho^{\alpha_{1}+\alpha_{2}}\cos\left(\frac{\pi (\alpha_{1}+\alpha_{2})}{2}\right)\\ \; & +\left(\mu +\beta B^{*}+\beta L^{*}\right)\left(\mu^{2}+\delta \mu \right)\varrho^{\alpha_{2}+\alpha_{3}}\cos\left(\frac{\pi (\alpha_{2}+\alpha_{3})}{2}\right)\\ \; & +\left(\varepsilon +\mu -\beta S^{*}\right)\left(\mu^{2}+\delta \mu \right)\varrho^{\alpha_{3}+\alpha_{1}}\cos\left(\frac{\pi (\alpha_{3}+\alpha_{1})}{2}\right)\\ \; & +\left(\mu^{2}+\varepsilon \mu -\beta S^{*}\varepsilon -\beta S^{*}\mu +\delta \varepsilon +\delta \mu -\beta S^{*}\delta \right)\left(\mu +\delta \right)\varrho^{2\alpha_{1}}\\ \; & +\left(\mu^{2}+\beta B^{*}\mu +\beta L^{*}\mu +\delta \mu +\beta B^{*}\delta +\beta L^{*}\delta \right)\left(\mu +\delta \right)\varrho^{\alpha_{1}+\alpha_{2}}\cos\left(\frac{\pi (\alpha_{2}-\alpha_{1})}{2}\right)\\ \; & +\left(\mu^{2}+\varepsilon \mu +\beta B^{*}\varepsilon +\beta B^{*}\mu +\beta L^{*}\varepsilon +\beta L^{*}\mu -\beta S^{*}\mu \right)\left(\mu +\delta \right)\varrho^{\alpha_{3}+\alpha_{1}}\cos\left(\frac{\pi (\alpha_{1}-\alpha_{3})}{2}\right)\\ \; & +\mu \left(\mu^{2}+\varepsilon \mu -\beta S^{*}\varepsilon -\beta S^{*}\mu +\delta \varepsilon +\delta \mu -\beta S^{*}\delta \right)\varrho^{\alpha_{3}+\alpha_{1}}\cos\left(\frac{\pi (\alpha_{1}-\alpha_{3})}{2}\right)\\ \; & +\mu \left(\mu^{2}+\beta B^{*}\mu +\beta L^{*}\mu +\delta \mu +\beta B^{*}\delta +\beta L^{*}\delta \right)\varrho^{\alpha_{2}+\alpha_{3}}\cos\left(\frac{\pi (\alpha_{3}-\alpha_{2})}{2}\right)\\ \; & +\mu \left(\mu^{2}+\varepsilon \mu +\beta B^{*}\varepsilon +\beta B^{*}\mu +\beta L^{*}\varepsilon +\beta L^{*}\mu -\beta S^{*}\mu \right)\varrho^{2\alpha_{3}}\\ \; & +(\mu^{3}+\varepsilon \mu^{2}+B^{*}\beta \mu^{2}+L^{*}\beta \mu^{2}-S^{*}\beta \mu^{2}+B^{*}\beta \varepsilon \mu +L^{*}\beta \varepsilon \mu -S^{*}\beta \varepsilon \mu \\ \; & \;\;+\delta \mu^{2}+\delta \varepsilon \mu +L^{*}\beta \delta \mu -S^{*}\beta \delta \mu +B^{*}\beta \delta \mu )\varrho^{\alpha_{3}+\alpha_{1}}\cos\left(\frac{\pi (\alpha_{3}+\alpha_{1})}{2}\right), \end{array}$$

(48) $$\begin{array}{cc} H_{04I2}\left(\varrho \right)= & -\left(\mu +\delta \right)\left(\mu^{2}+\delta \mu \right)\varrho^{\alpha_{1}+\alpha_{2}}\sin\left(\frac{\pi (\alpha_{1}+\alpha_{2})}{2}\right)\\ \; & -\left(\mu +\beta B^{*}+\beta L^{*}\right)\left(\mu^{2}+\delta \mu \right)\varrho^{\alpha_{2}+\alpha_{3}}\sin\left(\frac{\pi (\alpha_{2}+\alpha_{3})}{2}\right)\\ \; & -\left(\varepsilon +\mu -\beta S^{*}\right)\left(\mu^{2}+\delta \mu \right)\varrho^{\alpha_{3}+\alpha_{1}}\sin\left(\frac{\pi (\alpha_{3}+\alpha_{1})}{2}\right)\\ \; & -\left(\mu^{2}+\beta B^{*}\mu +\beta L^{*}\mu +\delta \mu +\beta B^{*}\delta +\beta L^{*}\delta \right)\left(\mu +\delta \right)\varrho^{\alpha_{1}+\alpha_{2}}\sin\left(\frac{\pi (\alpha_{2}-\alpha_{1})}{2}\right)\\ \; & +\left(\mu^{2}+\varepsilon \mu +\beta B^{*}\varepsilon +\beta B^{*}\mu +\beta L^{*}\varepsilon +\beta L^{*}\mu -\beta S^{*}\mu \right)\left(\mu +\delta \right)\varrho^{\alpha_{3}+\alpha_{1}}\sin\left(\frac{\pi (\alpha_{1}-\alpha_{3})}{2}\right)\\ \; & -\mu \left(\mu^{2}+\varepsilon \mu -\beta S^{*}\varepsilon -\beta S^{*}\mu +\delta \varepsilon +\delta \mu -\beta S^{*}\delta \right)\varrho^{\alpha_{3}+\alpha_{1}}\sin\left(\frac{\pi (\alpha_{1}-\alpha_{3})}{2}\right)\\ \; & +\mu \left(\mu^{2}+\beta B^{*}\mu +\beta L^{*}\mu +\delta \mu +\beta B^{*}\delta +\beta L^{*}\delta \right)\varrho^{\alpha_{2}+\alpha_{3}}\sin\left(\frac{\pi (\alpha_{3}-\alpha_{2})}{2}\right)\\ \; & +(\mu^{3}+\varepsilon \mu^{2}+B^{*}\beta \mu^{2}+L^{*}\beta \mu^{2}-S^{*}\beta \mu^{2}+B^{*}\beta \varepsilon \mu +L^{*}\beta \varepsilon \mu -S^{*}\beta \varepsilon \mu \\ \; & \;\;+\delta \mu^{2}+\delta \varepsilon \mu +L^{*}\beta \delta \mu -S^{*}\beta \delta \mu +B^{*}\beta \delta \mu )\varrho^{\alpha_{3}+\alpha_{1}}\sin\left(\frac{\pi (\alpha_{3}+\alpha_{1})}{2}\right), \end{array}$$

(49) $$\begin{array}{cc} H_{04R3}\left(\varrho \right)= & \varrho^{\alpha_{1}+\alpha_{2}+\alpha_{3}}\left(\mu^{2}+\delta \mu \right)\cos\left(\frac{\pi (\alpha_{1}+\alpha_{2}+\alpha_{3})}{2}\right)+{\left(\mu +\delta \right)}^{2}\varrho^{2\alpha_{1}+\alpha_{2}}\cos\left(\frac{\pi \alpha_{2}}{2}\right)\\ \; & +\left(\mu +\beta B^{*}+\beta L^{*}\right)\left(\mu +\delta \right)\varrho^{\alpha_{1}+\alpha_{2}+\alpha_{3}}\cos\left(\frac{\pi (\alpha_{2}+\alpha_{3}-\alpha_{1})}{2}\right)\\ \; & +\left(\varepsilon +\mu -\beta S^{*}\right)\left(\mu +\delta \right)\varrho^{\alpha_{3}+2\alpha_{1}}\cos\left(\frac{\pi \alpha_{3}}{2}\right)+\mu \left(\mu +\delta \right)\varrho^{\alpha_{1}+\alpha_{2}+\alpha_{3}}\cos\left(\frac{\pi (\alpha_{1}+\alpha_{2}-\alpha_{3})}{2}\right)\\ \; & +\mu \left(\mu +\beta B^{*}+\beta L^{*}\right)\varrho^{\alpha_{2}+2\alpha_{3}}\cos\left(\frac{\pi \alpha_{2}}{2}\right)+\mu \left(\varepsilon +\mu -\beta S^{*}\right)\varrho^{2\alpha_{3}+\alpha_{1}}\cos\left(\frac{\pi \alpha_{1}}{2}\right)\\ \; & +\left(\mu^{2}+\varepsilon \mu -\beta S^{*}\varepsilon -\beta S^{*}\mu +\delta \varepsilon +\delta \mu -\beta S^{*}\delta \right)\varrho^{\alpha_{3}+2\alpha_{1}}\cos\left(\frac{\pi \alpha_{3}}{2}\right)\\ \; & +\left(\mu^{2}+\beta B^{*}\mu +\beta L^{*}\mu +\delta \mu +\beta B^{*}\delta +\beta L^{*}\delta \right)\varrho^{\alpha_{1}+\alpha_{2}+\alpha_{3}}\cos\left(\frac{\pi (\alpha_{1}-\alpha_{2}+\alpha_{3})}{2}\right)\\ \; & +\left(\mu^{2}+\varepsilon \mu +\beta B^{*}\varepsilon +\beta B^{*}\mu +\beta L^{*}\varepsilon +\beta L^{*}\mu -\beta S^{*}\mu \right)\varrho^{2\alpha_{3}+\alpha_{1}}\cos\left(\frac{\pi \alpha_{1}}{2}\right), \end{array}$$

(50) $$\begin{array}{cc} H_{04I3}\left(\varrho \right)= & -\varrho^{\alpha_{1}+\alpha_{2}+\alpha_{3}}\left(\mu^{2}+\delta \mu \right)\sin\left(\frac{\pi (\alpha_{1}+\alpha_{2}+\alpha_{3})}{2}\right)-{\left(\mu +\delta \right)}^{2}\varrho^{2\alpha_{1}+\alpha_{2}}\sin\left(\frac{\pi \alpha_{2}}{2}\right)\\ \; & -\left(\mu +\beta B^{*}+\beta L^{*}\right)\left(\mu +\delta \right)\varrho^{\alpha_{1}+\alpha_{2}+\alpha_{3}}\sin\left(\frac{\pi (\alpha_{2}+\alpha_{3}-\alpha_{1})}{2}\right)-\left(\varepsilon +\mu -\beta S^{*}\right)\left(\mu +\delta \right)\varrho^{\alpha_{3}+2\alpha_{1}}\sin\left(\frac{\pi \alpha_{3}}{2}\right)\\ \; & -\mu \left(\mu +\delta \right)\varrho^{\alpha_{1}+\alpha_{2}+\alpha_{3}}\sin\left(\frac{\pi (\alpha_{1}+\alpha_{2}-\alpha_{3})}{2}\right)-\mu \left(\mu +\beta B^{*}+\beta L^{*}\right)\varrho^{\alpha_{2}+2\alpha_{3}}\sin\left(\frac{\pi \alpha_{2}}{2}\right)\\ \; & -\mu \left(\varepsilon +\mu -\beta S^{*}\right)\varrho^{2\alpha_{3}+\alpha_{1}}\sin\left(\frac{\pi \alpha_{1}}{2}\right)+\left(\mu^{2}+\varepsilon \mu -\beta S^{*}\varepsilon -\beta S^{*}\mu +\delta \varepsilon +\delta \mu -\beta S^{*}\delta \right)\varrho^{\alpha_{3}+2\alpha_{1}}\sin\left(\frac{\pi \alpha_{3}}{2}\right)\\ \; & +\left(\mu^{2}+\beta B^{*}\mu +\beta L^{*}\mu +\delta \mu +\beta B^{*}\delta +\beta L^{*}\delta \right)\varrho^{\alpha_{1}+\alpha_{2}+\alpha_{3}}\sin\left(\frac{\pi (\alpha_{1}-\alpha_{2}+\alpha_{3})}{2}\right)\\ \; & +\left(\mu^{2}+\varepsilon \mu +\beta B^{*}\varepsilon +\beta B^{*}\mu +\beta L^{*}\varepsilon +\beta L^{*}\mu -\beta S^{*}\mu \right)\varrho^{2\alpha_{3}+\alpha_{1}}\sin\left(\frac{\pi \alpha_{1}}{2}\right), \end{array}$$

(51) $$\begin{array}{cc} H_{04R4}\left(\varrho \right)= & \left(\mu +\delta \right)\varrho^{2\alpha_{1}+\alpha_{2}+\alpha_{3}}\cos\left(\frac{\pi (\alpha_{2}+\alpha_{3})}{2}\right)+\mu \varrho^{\alpha_{1}+\alpha_{2}+2\alpha_{3}}\cos\left(\frac{\pi (\alpha_{1}+\alpha_{2})}{2}\right)\\ \; & +\left(\mu +\delta \right)\varrho^{2\alpha_{1}+\alpha_{2}+\alpha_{3}}\cos\left(\frac{\pi (\alpha_{3}-\alpha_{2})}{2}\right)+\left(\mu +\beta B^{*}+\beta L^{*}\right)\varrho^{\alpha_{1}+\alpha_{2}+2\alpha_{3}}\cos\left(\frac{\pi (\alpha_{2}-\alpha_{1})}{2}\right)\\ \; & +\left(\varepsilon +\mu -\beta S^{*}\right)\varrho^{2\alpha_{3}+2\alpha_{1}}, \end{array}$$

and

(52) $$\begin{array}{cc} H_{04I4}\left(\varrho \right)= & -\left(\mu +\delta \right)\varrho^{2\alpha_{1}+\alpha_{2}+\alpha_{3}}\sin\left(\frac{\pi (\alpha_{2}+\alpha_{3})}{2}\right)-\mu \varrho^{\alpha_{1}+\alpha_{2}+2\alpha_{3}}\sin\left(\frac{\pi (\alpha_{1}+\alpha_{2})}{2}\right)\\ \; & +\left(\mu +\delta \right)\varrho^{2\alpha_{1}+\alpha_{2}+\alpha_{3}}\sin\left(\frac{\pi (\alpha_{3}-\alpha_{2})}{2}\right)-\left(\mu +\beta B^{*}+\beta L^{*}\right)\varrho^{\alpha_{1}+\alpha_{2}+2\alpha_{3}}\sin\left(\frac{\pi (\alpha_{2}-\alpha_{1})}{2}\right). \end{array}$$

Let us solve Equation (41) for $\vartheta_{1}$, to get

(53) $$\vartheta_{1}=\frac{1}{\varrho_{1}}\left(2\kappa \pi +\arccos\left(-\frac{H_{04R}\left(\varrho_{1}\right)}{|H_{02}\left(\varrho_{1}\right){|}^{2}}\right)\right),$$

with the integer κ taken such that the right-hand side of Equation (53) is positive.

Combining Equations (43)–(53) guarantees that the positive constant $\overset{´}{\vartheta }$ (resp., $\overset{´}{\varrho }$), given by Equation (24) (resp., Equation (25)), is well defined.

With the aid of Equations (19) and (20), we obtain

(54) $$\begin{array}{cc} \frac{\partial }{\partial \xi }▽\left(\xi ;\vartheta ,0\right)= & \left(\alpha_{1}+\alpha_{2}+\alpha_{3}\right)\xi^{\alpha_{1}+\alpha_{2}+\alpha_{3}-1}+\left(\alpha_{1}+\alpha_{2}\right)\left(\mu +\delta \right)\xi^{\alpha_{1}+\alpha_{2}-1}\\ \; & +\left(\alpha_{2}+\alpha_{3}\right)\left(\mu +\beta B^{*}+\beta L^{*}\right)\xi^{\alpha_{2}+\alpha_{3}-1}-\gamma \vartheta e^{-\vartheta \xi }\xi^{\alpha_{3}+\alpha_{1}}\\ \; & +\left(\alpha_{3}+\alpha_{1}\right)\left(\varepsilon +\mu -\beta S^{*}+\gamma e^{-\vartheta \xi }\right)\xi^{\alpha_{3}+\alpha_{1}-1}-\left(\gamma \mu \vartheta e^{-\vartheta \xi }+\delta \gamma \vartheta e^{-\xi \vartheta }\right)\xi^{\alpha_{1}}\\ \; & +\alpha_{1}\left(\mu^{2}+\varepsilon \mu -\beta S^{*}\varepsilon -\beta S^{*}\mu +\gamma \mu e^{-\vartheta \xi }+\delta \varepsilon +\delta \mu -\beta S^{*}\delta +\delta \gamma e^{-\xi \vartheta }\right)\xi^{\alpha_{1}-1}\\ \; & +\alpha_{2}\left(\mu^{2}+\beta B^{*}\mu +\beta L^{*}\mu +\delta \mu +\beta B^{*}\delta +\beta L^{*}\delta \right)\xi^{\alpha_{2}-1}\\ \; & -\gamma \mu \vartheta e^{-\vartheta \xi }\xi^{\alpha_{3}}+\alpha_{3}\left(\mu^{2}+\varepsilon \mu +\beta B^{*}\varepsilon +\beta B^{*}\mu +\beta L^{*}\varepsilon +\beta L^{*}\mu -\beta S^{*}\mu +\gamma \mu e^{-\vartheta \xi }\right)\xi^{\alpha_{3}-1}\\ \; & -(\gamma \mu^{2}\vartheta e^{-\vartheta \xi }+\delta \gamma \mu \vartheta e^{-\xi \vartheta }), \end{array}$$

and

(55) $$\begin{array}{cc} \frac{\partial }{\partial \vartheta }▽\left(\xi ;\vartheta ,0\right)= & -\gamma e^{-\vartheta \xi }\xi^{\alpha_{3}+\alpha_{1}+1}-\left(\gamma \mu e^{-\vartheta \xi }+\delta \gamma e^{-\xi \vartheta }\right)\xi^{\alpha_{1}+1}\\ \; & -\gamma \mu e^{-\vartheta \xi }\xi^{\alpha_{3}+1}-\gamma \mu^{2}e^{-\vartheta \xi }\xi -\delta \gamma \mu e^{-\xi \vartheta }\xi . \end{array}$$

This, together with Equation (54), implies

(56) $${\left(\frac{d\xi }{d\vartheta }\right)}^{-1}=\frac{\frac{\partial }{\partial \xi }▽\left(\xi ;\vartheta ,0\right)}{\frac{\partial }{\partial \vartheta }▽\left(\xi ;\vartheta ,0\right)}=\frac{\vartheta }{\xi }-\frac{H_{05}\left(\varrho i,\vartheta \right)}{\gamma \xi^{2}H_{06}\left(\varrho i,\vartheta \right)},$$

where $H_{05}\left(\varrho i,\vartheta \right)$ and $H_{06}\left(\varrho i,\vartheta \right)$ are given respectively by

(57) $$\begin{array}{cc} H_{05}= & \left(\alpha_{1}+\alpha_{2}+\alpha_{3}\right)\xi^{\alpha_{1}+\alpha_{2}+\alpha_{3}}\\ \; & +\left(\alpha_{1}+\alpha_{2}\right)\left(\mu +\delta \right)\xi^{\alpha_{1}+\alpha_{2}}\\ \; & +\left(\alpha_{2}+\alpha_{3}\right)\left(\mu +\beta B^{*}+\beta L^{*}\right)\xi^{\alpha_{2}+\alpha_{3}}\\ \; & +\left(\alpha_{3}+\alpha_{1}\right)\left(\varepsilon +\mu -\beta S^{*}+\gamma e^{-\vartheta \xi }\right)\xi^{\alpha_{3}+\alpha_{1}}\\ \; & +\alpha_{1}\left(\mu^{2}+\varepsilon \mu -\beta S^{*}\varepsilon -\beta S^{*}\mu +\gamma \mu e^{-\vartheta \xi }+\delta \varepsilon +\delta \mu -\beta S^{*}\delta +\delta \gamma e^{-\xi \vartheta }\right)\xi^{\alpha_{1}}\\ \; & +\alpha_{2}\left(\mu^{2}+\beta B^{*}\mu +\beta L^{*}\mu +\delta \mu +\beta B^{*}\delta +\beta L^{*}\delta \right)\xi^{\alpha_{2}}\\ \; & +\alpha_{3}\left(\mu^{2}+\varepsilon \mu +\beta B^{*}\varepsilon +\beta B^{*}\mu +\beta L^{*}\varepsilon +\beta L^{*}\mu -\beta S^{*}\mu +\gamma \mu e^{-\vartheta \xi }\right)\xi^{\alpha_{3}} \end{array}$$

and

(58) $$\begin{array}{cc} H_{06}= & e^{-\vartheta \xi }\xi^{\alpha_{3}+\alpha_{1}}+\left(\mu e^{-\vartheta \xi }+\delta e^{-\xi \vartheta }\right)\xi^{\alpha_{1}}\\ \; & +\mu e^{-\vartheta \xi }\xi^{\alpha_{3}}+\mu^{2}e^{-\vartheta \xi }+\delta \mu e^{-\xi \vartheta }. \end{array}$$

By straightforward calculations, we have further

(59) $$\begin{array}{cc} H_{05}\left(\varrho i\right)= & \left(\alpha_{1}+\alpha_{2}+\alpha_{3}\right){\left(\varrho i\right)}^{\alpha_{1}+\alpha_{2}+\alpha_{3}}\\ \; & +\left(\alpha_{1}+\alpha_{2}\right)\left(\mu +\delta \right){\left(\varrho i\right)}^{\alpha_{1}+\alpha_{2}}\\ \; & +\left(\alpha_{2}+\alpha_{3}\right)\left(\mu +\beta B^{*}+\beta L^{*}\right){\left(\varrho i\right)}^{\alpha_{2}+\alpha_{3}}\\ \; & +\left(\alpha_{3}+\alpha_{1}\right)\left(\varepsilon +\mu -\beta S^{*}+\gamma e^{-\vartheta \varrho i}\right){\left(\varrho i\right)}^{\alpha_{3}+\alpha_{1}}\\ \; & +\alpha_{1}\left(\mu^{2}+\varepsilon \mu -\beta S^{*}\varepsilon -\beta S^{*}\mu +\gamma \mu e^{-\vartheta \varrho i}+\delta \varepsilon +\delta \mu -\beta S^{*}\delta +\delta \gamma e^{-\vartheta \varrho i}\right){\left(\varrho i\right)}^{\alpha_{1}}\\ \; & +\alpha_{2}\left(\mu^{2}+\beta B^{*}\mu +\beta L^{*}\mu +\delta \mu +\beta B^{*}\delta +\beta L^{*}\delta \right){\left(\varrho i\right)}^{\alpha_{2}}\\ \; & +\alpha_{3}\left(\mu^{2}+\varepsilon \mu +\beta B^{*}\varepsilon +\beta B^{*}\mu +\beta L^{*}\varepsilon +\beta L^{*}\mu -\beta S^{*}\mu +\gamma \mu e^{-\vartheta \varrho i}\right){\left(\varrho i\right)}^{\alpha_{3}}\\ = & \left(\alpha_{1}+\alpha_{2}+\alpha_{3}\right)\varrho^{\alpha_{1}+\alpha_{2}+\alpha_{3}}e^{\frac{\pi i(\alpha_{1}+\alpha_{2}+\alpha_{3})}{2}}\\ \; & +\left(\alpha_{1}+\alpha_{2}\right)\left(\mu +\delta \right)\varrho^{\alpha_{1}+\alpha_{2}}e^{\frac{\pi i(\alpha_{1}+\alpha_{2})}{2}}\\ \; & +\left(\alpha_{2}+\alpha_{3}\right)\left(\mu +\beta B^{*}+\beta L^{*}\right)\varrho^{\alpha_{2}+\alpha_{3}}e^{\frac{\pi i(\alpha_{2}+\alpha_{3})}{2}}\\ \; & +\left(\alpha_{3}+\alpha_{1}\right)\left(\varepsilon +\mu -\beta S^{*}+\gamma e^{-\vartheta \varrho i}\right)\varrho^{\alpha_{3}+\alpha_{1}}e^{\frac{\pi i(\alpha_{3}+\alpha_{1})}{2}}\\ \; & +\alpha_{1}\left(\mu^{2}+\varepsilon \mu -\beta S^{*}\varepsilon -\beta S^{*}\mu +\gamma \mu e^{-\vartheta \varrho i}+\delta \varepsilon +\delta \mu -\beta S^{*}\delta +\delta \gamma e^{-\vartheta \varrho i}\right)\varrho^{\alpha_{1}}e^{\frac{\pi i\alpha_{1}}{2}}\\ \; & +\alpha_{2}\left(\mu^{2}+\beta B^{*}\mu +\beta L^{*}\mu +\delta \mu +\beta B^{*}\delta +\beta L^{*}\delta \right)\varrho^{\alpha_{2}}e^{\frac{\pi i\alpha_{2}}{2}}\\ \; & +\alpha_{3}\left(\mu^{2}+\varepsilon \mu +\beta B^{*}\varepsilon +\beta B^{*}\mu +\beta L^{*}\varepsilon +\beta L^{*}\mu -\beta S^{*}\mu +\gamma \mu e^{-\vartheta \varrho i}\right)\varrho^{\alpha_{3}}e^{\frac{\pi i\alpha_{3}}{2}} \end{array}$$

and

(60) $$\begin{array}{cc} H_{06}\left(\varrho i\right)= & e^{-\vartheta \varrho i}{\left(\varrho i\right)}^{\alpha_{3}+\alpha_{1}}+\left(\mu e^{-\vartheta \varrho i}+\delta e^{-\vartheta \varrho i}\right){\left(\varrho i\right)}^{\alpha_{1}}\\ \; & +\mu e^{-\vartheta \varrho i}{\left(\varrho i\right)}^{\alpha_{3}}+\mu^{2}e^{-\vartheta \varrho i}+\delta \mu e^{-\vartheta \varrho i}\\ = & \varrho^{\alpha_{3}+\alpha_{1}}e^{i(\frac{\pi (\alpha_{3}+\alpha_{1})}{2}-\vartheta \varrho )}+\left(\mu +\delta \right)e^{i(\frac{\pi \alpha_{1}}{2}-\vartheta \varrho )}\\ \; & +\mu e^{i(\frac{\pi \alpha_{3}}{2}-\vartheta \varrho )}+\left(\mu^{2}+\delta \mu \right)e^{-\vartheta \varrho i}. \end{array}$$

Employing Equation (56) as a central tool, a series of routine calculations leads to

(61) $$\begin{array}{cc} {\left({\left.\frac{d\xi }{d\vartheta }\right|}_{\xi =\overset{´}{\varrho }i,\vartheta =\overset{´}{\vartheta }}\right)}^{-1}= & -\frac{\overset{´}{\vartheta }i}{\overset{´}{\varrho }}+\frac{\bar{H_{06}\left(\overset{´}{\varrho }i,\overset{´}{\vartheta }\right)}H_{05}\left(\overset{´}{\varrho }i,\overset{´}{\vartheta }\right)}{\gamma {\left(\overset{´}{\varrho }\right)}^{2}{\left|H_{06}\left(\overset{´}{\varrho }i,\overset{´}{\vartheta }\right)\right|}^{2}}\\ = & \frac{W_{2}\left(\overset{´}{\varrho },\overset{´}{\vartheta }\right)}{\gamma {\left(\overset{´}{\varrho }\right)}^{2}{\left|H_{06}\left(\overset{´}{\varrho }i,\overset{´}{\vartheta }\right)\right|}^{2}}+i\left(\frac{H_{07}\left(\overset{´}{\varrho },\overset{´}{\vartheta }\right)}{\gamma {\left(\overset{´}{\varrho }\right)}^{2}{\left|H_{06}\left(\overset{´}{\varrho }i,\overset{´}{\vartheta }\right)\right|}^{2}}-\frac{\overset{´}{\vartheta }}{\overset{´}{\varrho }}\right), \end{array}$$

in which $W_{2}\left(\varrho ,\vartheta \right)$ and $H_{07}\left(\varrho ,\vartheta \right)$ are given respectively by Equation (23) and

(62) $$\begin{array}{cc} H_{07}\left(\varrho ,\vartheta \right)= & \left(\alpha_{1}+\alpha_{2}+\alpha_{3}\right)\varrho^{2\alpha_{1}+\alpha_{2}+2\alpha_{3}}\sin\left(\frac{\pi \alpha_{2}}{2}+\vartheta \varrho \right)+\left(\alpha_{1}+\alpha_{2}\right)\left(\mu +\delta \right)\varrho^{2\alpha_{1}+\alpha_{2}+\alpha_{3}}\sin\left(\frac{\pi (\alpha_{2}-\alpha_{3})}{2}+\vartheta \varrho \right)\\ \; & +\left(\alpha_{2}+\alpha_{3}\right)\left(\mu +\beta B^{*}+\beta L^{*}\right)\varrho^{\alpha_{1}+\alpha_{2}+2\alpha_{3}}\sin\left(\frac{\pi (\alpha_{2}-\alpha_{1})}{2}+\vartheta \varrho \right)-\gamma \left(\alpha_{3}+\alpha_{1}\right)\varrho^{2\alpha_{3}+2\alpha_{1}}\sin\left(\vartheta \varrho \right)\\ \; & +\alpha_{1}\left(\mu^{2}+\varepsilon \mu -\beta S^{*}\varepsilon -\beta S^{*}\mu +\delta \varepsilon +\delta \mu -\beta S^{*}\delta \right)\varrho^{\alpha_{3}+2\alpha_{1}}\sin\left(\vartheta \varrho -\frac{\pi \alpha_{3}}{2}\right)-\alpha_{1}\gamma \left(\mu +\delta \right)\varrho^{\alpha_{3}+2\alpha_{1}}\sin\left(\frac{\pi \alpha_{3}}{2}\right)\\ \; & +\alpha_{2}\left(\mu^{2}+\beta B^{*}\mu +\beta L^{*}\mu +\delta \mu +\beta B^{*}\delta +\beta L^{*}\delta \right)\varrho^{\alpha_{1}+\alpha_{2}+\alpha_{3}}\sin\left(\frac{\pi (\alpha_{2}-\alpha_{1})}{2}+\vartheta \varrho \right)\\ \; & +\alpha_{3}\left(\mu^{2}+\varepsilon \mu +\beta B^{*}\varepsilon +\beta B^{*}\mu +\beta L^{*}\varepsilon +\beta L^{*}\mu -\beta S^{*}\mu \right)\varrho^{2\alpha_{3}+\alpha_{1}}\sin\left(\vartheta \varrho -\frac{\pi \alpha_{1}}{2}\right)-\alpha_{3}\gamma \mu \varrho^{2\alpha_{3}+\alpha_{1}}\sin\left(\frac{\pi \alpha_{1}}{2}\right)\\ \; & +\left(\mu +\delta \right)\left(\alpha_{1}+\alpha_{2}+\alpha_{3}\right)\varrho^{\alpha_{1}+\alpha_{2}+\alpha_{3}}\sin\left(\frac{\pi (\alpha_{2}+\alpha_{3})}{2}+\vartheta \varrho \right)+\left(\alpha_{1}+\alpha_{2}\right){\left(\mu +\delta \right)}^{2}\varrho^{\alpha_{1}+\alpha_{2}}\sin\left(\vartheta \varrho +\frac{\pi \alpha_{2}}{2}\right)\\ \; & +\left(\mu +\delta \right)\left(\alpha_{2}+\alpha_{3}\right)\left(\mu +\beta B^{*}+\beta L^{*}\right)\varrho^{\alpha_{2}+\alpha_{3}}\sin\left(\frac{\pi (\alpha_{2}+\alpha_{3}-\alpha_{1})}{2}+\vartheta \varrho \right)\\ \; & +\left(\mu +\delta \right)\left(\alpha_{3}+\alpha_{1}\right)\left(\varepsilon +\mu -\beta S^{*}\right)\varrho^{\alpha_{3}+\alpha_{1}}\sin\left(\vartheta \varrho +\frac{\pi \alpha_{3}}{2}\right)+\gamma \left(\mu +\delta \right)\left(\alpha_{3}+\alpha_{1}\right)\varrho^{\alpha_{3}+\alpha_{1}}\sin\left(\frac{\pi \alpha_{3}}{2}\right)\\ \; & +\alpha_{1}\left(\mu +\delta \right)\left(\mu^{2}+\varepsilon \mu -\beta S^{*}\varepsilon -\beta S^{*}\mu +\delta \varepsilon +\delta \mu -\beta S^{*}\delta \right)\varrho^{\alpha_{1}}\sin\left(\vartheta \varrho \right)\\ \; & +\alpha_{2}\left(\mu +\delta \right)\left(\mu^{2}+\beta B^{*}\mu +\beta L^{*}\mu +\delta \mu +\beta B^{*}\delta +\beta L^{*}\delta \right)\varrho^{\alpha_{2}}\sin\left(\frac{\pi (\alpha_{2}-\alpha_{1})}{2}+\vartheta \varrho \right)\\ \; & +\alpha_{3}\left(\mu +\delta \right)\left(\mu^{2}+\varepsilon \mu +\beta B^{*}\varepsilon +\beta B^{*}\mu +\beta L^{*}\varepsilon +\beta L^{*}\mu -\beta S^{*}\mu \right)\varrho^{\alpha_{3}}\sin\left(\frac{\pi (\alpha_{3}-\alpha_{1})}{2}+\vartheta \varrho \right)\\ \; & +\alpha_{3}\gamma \mu \left(\mu +\delta \right)\varrho^{\alpha_{3}}\sin\left(\frac{\pi (\alpha_{3}-\alpha_{1})}{2}\right)+\mu \left(\alpha_{1}+\alpha_{2}+\alpha_{3}\right)\varrho^{\alpha_{1}+\alpha_{2}+\alpha_{3}}\sin\left(\frac{\pi (\alpha_{1}+\alpha_{2})}{2}+\vartheta \varrho \right)\\ \; & +\mu \left(\alpha_{1}+\alpha_{2}\right)\left(\mu +\delta \right)\varrho^{\alpha_{1}+\alpha_{2}}\sin\left(\frac{\pi (\alpha_{1}+\alpha_{2}-\alpha_{3})}{2}+\vartheta \varrho \right)\\ \; & +\mu \left(\alpha_{2}+\alpha_{3}\right)\left(\mu +\beta B^{*}+\beta L^{*}\right)\varrho^{\alpha_{2}+\alpha_{3}}\sin\left(\vartheta \varrho +\frac{\pi \alpha_{2}}{2}\right)\\ \; & +\mu \left(\alpha_{3}+\alpha_{1}\right)\left(\varepsilon +\mu -\beta S^{*}\right)\varrho^{\alpha_{3}+\alpha_{1}}\sin\left(\vartheta \varrho +\frac{\pi \alpha_{1}}{2}\right)+\mu \gamma \left(\alpha_{3}+\alpha_{1}\right)\varrho^{\alpha_{3}+\alpha_{1}}\sin\left(\frac{\pi \alpha_{1}}{2}\right)\\ \; & +\alpha_{1}\mu \left(\mu^{2}+\varepsilon \mu -\beta S^{*}\varepsilon -\beta S^{*}\mu +\delta \varepsilon +\delta \mu -\beta S^{*}\delta \right)\varrho^{\alpha_{1}}\sin\left(\frac{\pi (\alpha_{1}-\alpha_{3})}{2}+\vartheta \varrho \right)\\ \; & +\alpha_{1}\mu \gamma \left(\mu +\delta \right)\varrho^{\alpha_{1}}\sin\left(\frac{\pi (\alpha_{1}-\alpha_{3})}{2}\right)\\ \; & +\alpha_{2}\mu \left(\mu^{2}+\beta B^{*}\mu +\beta L^{*}\mu +\delta \mu +\beta B^{*}\delta +\beta L^{*}\delta \right)\varrho^{\alpha_{2}}\sin\left(\frac{\pi (\alpha_{2}-\alpha_{3})}{2}+\vartheta \varrho \right)\\ \; & +\alpha_{3}\mu \left(\mu^{2}+\varepsilon \mu +\beta B^{*}\varepsilon +\beta B^{*}\mu +\beta L^{*}\varepsilon +\beta L^{*}\mu -\beta S^{*}\mu \right)\varrho^{\alpha_{3}}\sin\left(\vartheta \varrho \right)\\ \; & +\left(\mu^{2}+\delta \mu \right)\left(\alpha_{1}+\alpha_{2}+\alpha_{3}\right)\varrho^{\alpha_{1}+\alpha_{2}+\alpha_{3}}\sin\left(\frac{\pi (\alpha_{1}+\alpha_{2}+\alpha_{3})}{2}+\vartheta \varrho \right)\\ \; & +\left(\mu^{2}+\delta \mu \right)\left(\alpha_{1}+\alpha_{2}\right)\left(\mu +\delta \right)\varrho^{\alpha_{1}+\alpha_{2}}\sin\left(\frac{\pi (\alpha_{1}+\alpha_{2})}{2}+\vartheta \varrho \right)\\ \; & +\left(\mu^{2}+\delta \mu \right)\left(\alpha_{2}+\alpha_{3}\right)\left(\mu +\beta B^{*}+\beta L^{*}\right)\varrho^{\alpha_{2}+\alpha_{3}}\sin\left(\frac{\pi (\alpha_{2}+\alpha_{3})}{2}+\vartheta \varrho \right)\\ \; & +\left(\mu^{2}+\delta \mu \right)\left(\alpha_{3}+\alpha_{1}\right)\left(\varepsilon +\mu -\beta S^{*}\right)\varrho^{\alpha_{3}+\alpha_{1}}\sin\left(\frac{\pi (\alpha_{3}+\alpha_{1})}{2}+\vartheta \varrho \right)\\ \; & +\gamma \left(\mu^{2}+\delta \mu \right)\left(\alpha_{3}+\alpha_{1}\right)\varrho^{\alpha_{3}+\alpha_{1}}\sin\left(\frac{\pi (\alpha_{3}+\alpha_{1})}{2}\right)+\alpha_{1}\gamma \left(\mu^{2}+\delta \mu \right)\left(\mu +\delta \right)\varrho^{\alpha_{1}}\sin\left(\frac{\pi \alpha_{1}}{2}\right)\\ \; & +\alpha_{1}\left(\mu^{2}+\delta \mu \right)\left(\mu^{2}+\varepsilon \mu -\beta S^{*}\varepsilon -\beta S^{*}\mu +\delta \varepsilon +\delta \mu -\beta S^{*}\delta \right)\varrho^{\alpha_{1}}\sin\left(\vartheta \varrho +\frac{\pi \alpha_{1}}{2}\right)\\ \; & +\alpha_{2}\left(\mu^{2}+\delta \mu \right)\left(\mu^{2}+\beta B^{*}\mu +\beta L^{*}\mu +\delta \mu +\beta B^{*}\delta +\beta L^{*}\delta \right)\varrho^{\alpha_{2}}\sin\left(\vartheta \varrho +\frac{\pi \alpha_{2}}{2}\right)\\ \; & +\alpha_{3}\left(\mu^{2}+\delta \mu \right)\left(\mu^{2}+\varepsilon \mu +\beta B^{*}\varepsilon +\beta B^{*}\mu +\beta L^{*}\varepsilon +\beta L^{*}\mu -\beta S^{*}\mu \right)\varrho^{\alpha_{3}}\sin\left(\vartheta \varrho +\frac{\pi \alpha_{2}}{2}\right)\\ \; & +\alpha_{3}\gamma \mu \left(\mu^{2}+\delta \mu \right)\varrho^{\alpha_{3}}\sin\left(\frac{\pi \alpha_{3}}{2}\right). \end{array}$$

In Equation (61), the term $\bar{H_{06}\left(\varrho i\right)}H_{05}\left(\varrho i\right)$ can be expressed explicitly as follows

(63) $$\begin{array}{cc} \bar{H_{06}\left(\varrho i\right)}H_{05}\left(\varrho i\right)= & \left(\alpha_{1}+\alpha_{2}+\alpha_{3}\right)\varrho^{2\alpha_{1}+\alpha_{2}+2\alpha_{3}}e^{i(\frac{\pi \alpha_{2}}{2}+\vartheta \varrho )}+\left(\alpha_{1}+\alpha_{2}\right)\left(\mu +\delta \right)\varrho^{2\alpha_{1}+\alpha_{2}+\alpha_{3}}e^{i(\frac{\pi (\alpha_{2}-\alpha_{3})}{2}+\vartheta \varrho )}\\ \; & +\left(\alpha_{2}+\alpha_{3}\right)\left(\mu +\beta B^{*}+\beta L^{*}\right)\varrho^{\alpha_{1}+\alpha_{2}+2\alpha_{3}}e^{i(\frac{\pi (\alpha_{2}-\alpha_{1})}{2}+\vartheta \varrho )}+\left(\alpha_{3}+\alpha_{1}\right)\left(\varepsilon +\mu -\beta S^{*}+\gamma e^{-\vartheta \varrho i}\right)\varrho^{2\alpha_{3}+2\alpha_{1}}\\ \; & +\alpha_{1}\left(\mu^{2}+\varepsilon \mu -\beta S^{*}\varepsilon -\beta S^{*}\mu +\gamma \mu e^{-\vartheta \varrho i}+\delta \varepsilon +\delta \mu -\beta S^{*}\delta +\delta \gamma e^{-\vartheta \varrho i}\right)\varrho^{\alpha_{3}+2\alpha_{1}}e^{i(\vartheta \varrho -\frac{\pi \alpha_{3}}{2})}\\ \; & +\alpha_{2}\left(\mu^{2}+\beta B^{*}\mu +\beta L^{*}\mu +\delta \mu +\beta B^{*}\delta +\beta L^{*}\delta \right)\varrho^{\alpha_{1}+\alpha_{2}+\alpha_{3}}e^{i(\frac{\pi (\alpha_{2}-\alpha_{1})}{2}+\vartheta \varrho )}\\ \; & +\alpha_{3}\left(\mu^{2}+\varepsilon \mu +\beta B^{*}\varepsilon +\beta B^{*}\mu +\beta L^{*}\varepsilon +\beta L^{*}\mu -\beta S^{*}\mu +\gamma \mu e^{-\vartheta \varrho i}\right)\varrho^{2\alpha_{3}+\alpha_{1}}e^{i(\vartheta \varrho -\frac{\pi \alpha_{1}}{2})}\\ \; & +\left(\mu +\delta \right)\left(\alpha_{1}+\alpha_{2}+\alpha_{3}\right)\varrho^{\alpha_{1}+\alpha_{2}+\alpha_{3}}e^{i(\frac{\pi (\alpha_{2}+\alpha_{3})}{2}+\vartheta \varrho )}+\left(\mu +\delta \right)\left(\alpha_{1}+\alpha_{2}\right)\left(\mu +\delta \right)\varrho^{\alpha_{1}+\alpha_{2}}e^{i(\vartheta \varrho +\frac{\pi \alpha_{2}}{2})}\\ \; & +\left(\mu +\delta \right)\left(\alpha_{2}+\alpha_{3}\right)\left(\mu +\beta B^{*}+\beta L^{*}\right)\varrho^{\alpha_{2}+\alpha_{3}}e^{i(\frac{\pi (\alpha_{2}+\alpha_{3}-\alpha_{1})}{2}+\vartheta \varrho )}\\ \; & +\left(\mu +\delta \right)\left(\alpha_{3}+\alpha_{1}\right)\left(\varepsilon +\mu -\beta S^{*}+\gamma e^{-\vartheta \varrho i}\right)\varrho^{\alpha_{3}+\alpha_{1}}e^{i(\vartheta \varrho +\frac{\pi \alpha_{3}}{2})}\\ \; & +\alpha_{1}\left(\mu +\delta \right)\left(\mu^{2}+\varepsilon \mu -\beta S^{*}\varepsilon -\beta S^{*}\mu +\gamma \mu e^{-\vartheta \varrho i}+\delta \varepsilon +\delta \mu -\beta S^{*}\delta +\delta \gamma e^{-\vartheta \varrho i}\right)\varrho^{\alpha_{1}}e^{i\vartheta \varrho }\\ \; & +\alpha_{2}\left(\mu +\delta \right)\left(\mu^{2}+\beta B^{*}\mu +\beta L^{*}\mu +\delta \mu +\beta B^{*}\delta +\beta L^{*}\delta \right)\varrho^{\alpha_{2}}e^{i(\frac{\pi (\alpha_{2}-\alpha_{1})}{2}+\vartheta \varrho )}\\ \; & +\alpha_{3}\left(\mu +\delta \right)\left(\mu^{2}+\varepsilon \mu +\beta B^{*}\varepsilon +\beta B^{*}\mu +\beta L^{*}\varepsilon +\beta L^{*}\mu -\beta S^{*}\mu +\gamma \mu e^{-\vartheta \varrho i}\right)\varrho^{\alpha_{3}}e^{i(\frac{\pi (\alpha_{3}-\alpha_{1})}{2}+\vartheta \varrho )}\\ \; & +\mu \left(\alpha_{1}+\alpha_{2}+\alpha_{3}\right)\varrho^{\alpha_{1}+\alpha_{2}+\alpha_{3}}e^{i(\frac{\pi (\alpha_{1}+\alpha_{2})}{2}+\vartheta \varrho )}+\mu \left(\alpha_{1}+\alpha_{2}\right)\left(\mu +\delta \right)\varrho^{\alpha_{1}+\alpha_{2}}e^{i(\frac{\pi (\alpha_{1}+\alpha_{2}-\alpha_{3})}{2}+\vartheta \varrho )}\\ \; & +\mu \left(\alpha_{2}+\alpha_{3}\right)(\left(\mu +\beta B^{*}+\beta L^{*}\right)\varrho^{\alpha_{2}+\alpha_{3}}e^{i(\vartheta \varrho +\frac{\pi \alpha_{2}}{2})}+\left(\varepsilon +\mu -\beta S^{*}+\gamma e^{-\vartheta \varrho i}\right)\varrho^{\alpha_{3}+\alpha_{1}}e^{i(\vartheta \varrho +\frac{\pi \alpha_{1}}{2})})\\ \; & +\alpha_{1}\mu \left(\mu^{2}+\varepsilon \mu -\beta S^{*}\varepsilon -\beta S^{*}\mu +\gamma \mu e^{-\vartheta \varrho i}+\delta \varepsilon +\delta \mu -\beta S^{*}\delta +\delta \gamma e^{-\vartheta \varrho i}\right)\varrho^{\alpha_{1}}e^{i(\frac{\pi (\alpha_{1}-\alpha_{3})}{2}+\vartheta \varrho )}\\ \; & +\alpha_{2}\mu \left(\mu^{2}+\beta B^{*}\mu +\beta L^{*}\mu +\delta \mu +\beta B^{*}\delta +\beta L^{*}\delta \right)\varrho^{\alpha_{2}}e^{i(\frac{\pi (\alpha_{2}-\alpha_{3})}{2}+\vartheta \varrho )}\\ \; & +\alpha_{3}\mu \left(\mu^{2}+\varepsilon \mu +\beta B^{*}\varepsilon +\beta B^{*}\mu +\beta L^{*}\varepsilon +\beta L^{*}\mu -\beta S^{*}\mu +\gamma \mu e^{-\vartheta \varrho i}\right)\varrho^{\alpha_{3}}e^{i\vartheta \varrho }\\ \; & +\left(\mu^{2}+\delta \mu \right)(\left(\alpha_{1}+\alpha_{2}+\alpha_{3}\right)\varrho^{\alpha_{1}+\alpha_{2}+\alpha_{3}}e^{i(\frac{\pi (\alpha_{1}+\alpha_{2}+\alpha_{3})}{2}+\vartheta \varrho )}+\left(\alpha_{1}+\alpha_{2}\right)\left(\mu +\delta \right)\varrho^{\alpha_{1}+\alpha_{2}}e^{i(\frac{\pi (\alpha_{1}+\alpha_{2})}{2}+\vartheta \varrho )})\\ \; & +\left(\mu^{2}+\delta \mu \right)\left(\alpha_{2}+\alpha_{3}\right)\left(\mu +\beta B^{*}+\beta L^{*}\right)\varrho^{\alpha_{2}+\alpha_{3}}e^{i(\frac{\pi (\alpha_{2}+\alpha_{3})}{2}+\vartheta \varrho )}\\ \; & +\left(\mu^{2}+\delta \mu \right)\left(\alpha_{3}+\alpha_{1}\right)\left(\varepsilon +\mu -\beta S^{*}+\gamma e^{-\vartheta \varrho i}\right)\varrho^{\alpha_{3}+\alpha_{1}}e^{i(\frac{\pi (\alpha_{3}+\alpha_{1})}{2}+\vartheta \varrho )}\\ \; & +\alpha_{1}\left(\mu^{2}+\delta \mu \right)\left(\mu^{2}+\varepsilon \mu -\beta S^{*}\varepsilon -\beta S^{*}\mu +\gamma \mu e^{-\vartheta \varrho i}+\delta \varepsilon +\delta \mu -\beta S^{*}\delta +\delta \gamma e^{-\vartheta \varrho i}\right)\varrho^{\alpha_{1}}e^{\frac{\pi i\alpha_{1}}{2}}e^{i\vartheta \varrho }\\ \; & +\alpha_{2}\left(\mu^{2}+\delta \mu \right)\left(\mu^{2}+\beta B^{*}\mu +\beta L^{*}\mu +\delta \mu +\beta B^{*}\delta +\beta L^{*}\delta \right)\varrho^{\alpha_{2}}e^{i(\frac{\pi \alpha_{2}}{2}+\vartheta \varrho )}\\ \; & +\alpha_{3}\left(\mu^{2}+\delta \mu \right)\left(\mu^{2}+\varepsilon \mu +\beta B^{*}\varepsilon +\beta B^{*}\mu +\beta L^{*}\varepsilon +\beta L^{*}\mu -\beta S^{*}\mu +\gamma \mu e^{-\vartheta \varrho i}\right)\varrho^{\alpha_{3}}e^{\frac{\pi i\alpha_{3}}{2}}e^{i\vartheta \varrho }. \end{array}$$

The positivity of $W_{2}\left(\overset{´}{\varrho },\overset{´}{\vartheta }\right)$, together with Equation (61), gives

(64) Re(dξdϑξ=ϱ´i,ϑ=ϑ´)−1=W2(ϱ´,ϑ´)γ(ϱ´)2|H06(ϱ´i,ϑ´)|2>0,

which implies further

(65) Re(dξdϑξ=ϱ´i,ϑ=ϑ´)=Re(dξdϑξ=ϱ´i,ϑ=ϑ´¯)=dξdϑξ=ϱ´i,ϑ=ϑ´2Re(dξdϑξ=ϱ´i,ϑ=ϑ´)−1>0.

This, together with the definition (24) of $\overset{´}{\vartheta }$, brings the proof of Theorem 1 to a close. □

To facilitate our later presentation, as with Equation (22), we define the notation $W_{3}$ as

(66) $$\begin{array}{cc} W_{3}= & |\mu^{3}+\varepsilon \mu^{2}+B^{*}\beta \mu^{2}+L^{*}\beta \mu^{2}-S^{*}\beta \mu^{2}+B^{*}\beta \varepsilon \mu +L^{*}\beta \varepsilon \mu -S^{*}\beta \varepsilon \mu +\gamma \mu^{2}{|}^{2}\\ \; & -|\delta \mu^{2}+\delta \varepsilon \mu +L^{*}\beta \delta \mu -S^{*}\beta \delta \mu +B^{*}{\beta \delta \mu +\delta \gamma \mu |}^{2}, \end{array}$$

and as with Equation (23), we define $W_{4}\left(\varrho ,\vartheta \right)$ as

(67) $$\begin{array}{cc} W_{4}= & \left(\alpha_{1}+\alpha_{2}+\alpha_{3}\right)\varrho^{2\alpha_{1}+2\alpha_{2}+\alpha_{3}}\cos\left(\frac{\pi \alpha_{3}}{2}+\upsilon \varrho \right)+\mu \left(\alpha_{1}+\alpha_{2}\right)\varrho^{2\alpha_{1}+2\alpha_{2}}\cos\left(\upsilon \varrho \right)+\delta \left(\alpha_{1}+\alpha_{2}\right)\varrho^{2\alpha_{1}+2\alpha_{2}}\\ \; & +\left(\alpha_{2}+\alpha_{3}\right)\left(\mu +\beta B^{*}+\beta L^{*}\right)\varrho^{\alpha_{1}+2\alpha_{2}+\alpha_{3}}\cos\left(\frac{\pi (\alpha_{3}-\alpha_{1})}{2}+\upsilon \varrho \right)\\ \; & +\left(\alpha_{3}+\alpha_{1}\right)\left(\varepsilon +\mu -\beta S^{*}+\gamma \right)\varrho^{2\alpha_{1}+\alpha_{2}+\alpha_{3}}\cos\left(\frac{\pi (\alpha_{3}-\alpha_{2})}{2}+\upsilon \varrho \right)\\ \; & +\alpha_{1}\left(\mu^{2}+\varepsilon \mu -\beta S^{*}\varepsilon -\beta S^{*}\mu +\gamma \mu \right)\varrho^{2\alpha_{1}+\alpha_{2}}\cos\left(\upsilon \varrho -\frac{\pi \alpha_{2}}{2}\right)+\alpha_{1}\left(\delta \varepsilon +\delta \mu -\beta S^{*}\delta +\delta \gamma \right)\varrho^{2\alpha_{1}+\alpha_{2}}\cos\left(\frac{\pi \alpha_{2}}{2}\right)\\ \; & +\alpha_{2}\left(\mu +\beta B^{*}+\beta L^{*}\right)\varrho^{\alpha_{1}+2\alpha_{2}}\left(\mu \cos\left(\upsilon \varrho -\frac{\pi \alpha_{1}}{2}\right)+\delta \cos\left(\frac{\pi \alpha_{1}}{2}\right)\right)\\ \; & +\alpha_{3}\left(\mu^{2}+\varepsilon \mu +\beta B^{*}\varepsilon +\beta B^{*}\mu +\beta L^{*}\varepsilon +\beta L^{*}\mu -\beta S^{*}\mu +\gamma \mu \right)\varrho^{\alpha_{1}+\alpha_{2}+\alpha_{3}}\cos\left(\frac{\pi (\alpha_{3}-\alpha_{1}-\alpha_{2})}{2}+\upsilon \varrho \right)\\ \; & +\left(\varepsilon +\mu -\beta S^{*}+\gamma \right)\left(\alpha_{1}+\alpha_{2}+\alpha_{3}\right)\varrho^{2\alpha_{1}+\alpha_{2}+\alpha_{3}}\cos\left(\frac{\pi (\alpha_{2}+\alpha_{3})}{2}+\upsilon \varrho \right)\\ \; & +\left(\varepsilon +\mu -\beta S^{*}+\gamma \right)\left(\alpha_{1}+\alpha_{2}\right)\left(\mu \varrho^{2\alpha_{1}+\alpha_{2}}\cos\left(\frac{\pi \alpha_{2}}{2}+\upsilon \varrho \right)+\delta \varrho^{2\alpha_{1}+\alpha_{2}}\cos\left(\frac{\pi \alpha_{2}}{2}\right)\right)\\ \; & +\left(\varepsilon +\mu -\beta S^{*}+\gamma \right)\left(\alpha_{2}+\alpha_{3}\right)\left(\mu +\beta B^{*}+\beta L^{*}\right)\varrho^{\alpha_{1}+\alpha_{2}+\alpha_{3}}\cos\left(\frac{\pi (\alpha_{2}+\alpha_{3}-\alpha_{1})}{2}+\upsilon \varrho \right)\\ \; & +\left(\alpha_{3}+\alpha_{1}\right){\left(\varepsilon +\mu -\beta S^{*}+\gamma \right)}^{2}\varrho^{\alpha_{3}+2\alpha_{1}}\cos\left(\frac{\pi \alpha_{3}}{2}+\upsilon \varrho \right)\\ \; & +\alpha_{1}\left(\varepsilon +\mu -\beta S^{*}+\gamma \right)\varrho^{2\alpha_{1}}\left(\left(\mu^{2}+\varepsilon \mu -\beta S^{*}\varepsilon -\beta S^{*}\mu +\gamma \mu \right)\cos\left(\upsilon \varrho \right)+\delta \varepsilon +\delta \mu -\beta S^{*}\delta +\delta \gamma \right)\\ \; & +\alpha_{2}\left(\varepsilon +\mu -\beta S^{*}+\gamma \right)\left(\mu +\beta B^{*}+\beta L^{*}\right)\varrho^{\alpha_{1}+\alpha_{2}}\left(\mu \cos\left(\frac{\pi (\alpha_{2}-\alpha_{1})}{2}+\upsilon \varrho \right)+\delta \cos\left(\frac{\pi (\alpha_{2}-\alpha_{1})}{2}\right)\right)\\ \; & +\alpha_{3}\left(\varepsilon +\mu -\beta S^{*}+\gamma \right)\left(\mu^{2}+\varepsilon \mu +\beta B^{*}\varepsilon +\beta B^{*}\mu +\beta L^{*}\varepsilon +\beta L^{*}\mu -\beta S^{*}\mu +\gamma \mu \right)\varrho^{\alpha_{3}+\alpha_{1}}\cos\left(\frac{\pi (\alpha_{3}-\alpha_{1})}{2}+\upsilon \varrho \right)\\ \; & +\left(\mu +\beta B^{*}+\beta L^{*}\right)\left(\alpha_{1}+\alpha_{2}+\alpha_{3}\right)\varrho^{\alpha_{1}+2\alpha_{2}+\alpha_{3}}\cos\left(\frac{\pi (\alpha_{3}+\alpha_{1})}{2}+\upsilon \varrho \right)\\ \; & +\left(\mu +\beta B^{*}+\beta L^{*}\right)\left(\alpha_{1}+\alpha_{2}\right)\varrho^{\alpha_{1}+2\alpha_{2}}\left(\mu \cos\left(\frac{\pi \alpha_{1}}{2}+\upsilon \varrho \right)+\delta \cos\left(\frac{\pi \alpha_{1}}{2}\right)\right)\\ \; & +\left(\alpha_{2}+\alpha_{3}\right){\left(\mu +\beta B^{*}+\beta L^{*}\right)}^{2}\varrho^{2\alpha_{2}+\alpha_{3}}\cos\left(\frac{\pi \alpha_{3}}{2}+\upsilon \varrho \right)+\left(\mu +\beta B^{*}+\beta L^{*}\right)\left(\alpha_{3}+\alpha_{1}\right)(\varepsilon +\mu -\beta S^{*}\\ \; & +\gamma )\varrho^{\alpha_{1}+\alpha_{2}+\alpha_{3}}\cos\left(\frac{\pi (\alpha_{3}+\alpha_{1}-\alpha_{2})}{2}+\upsilon \varrho \right)+\alpha_{1}\left(\mu +\beta B^{*}+\beta L^{*}\right)(\mu^{2}+\varepsilon \mu -\beta S^{*}\varepsilon -\beta S^{*}\mu \\ \; & +\gamma \mu )\varrho^{\alpha_{1}+\alpha_{2}}\cos\left(\frac{\pi (\alpha_{1}-\alpha_{2})}{2}+\upsilon \varrho \right)+\alpha_{1}\left(\mu +\beta B^{*}+\beta L^{*}\right)\left(\delta \varepsilon +\delta \mu -\beta S^{*}\delta +\delta \gamma \right)\varrho^{\alpha_{1}+\alpha_{2}}\cos\left(\frac{\pi (\alpha_{1}-\alpha_{2})}{2}\right)\\ \; & +\alpha_{2}{\left(\mu +\beta B^{*}+\beta L^{*}\right)}^{2}\varrho^{2\alpha_{2}}\left(\mu \cos\left(\upsilon \varrho \right)+\delta \right)\\ \; & +\alpha_{3}\left(\mu +\beta B^{*}+\beta L^{*}\right)\left(\mu^{2}+\varepsilon \mu +\beta B^{*}\varepsilon +\beta B^{*}\mu +\beta L^{*}\varepsilon +\beta L^{*}\mu -\beta S^{*}\mu +\gamma \mu \right)\varrho^{\alpha_{2}+\alpha_{3}}\cos\left(\frac{\pi (\alpha_{3}-\alpha_{2})}{2}+\upsilon \varrho \right)\\ \; & +\left(\mu^{2}+\varepsilon \mu +L^{*}\beta \mu -S^{*}\beta \mu +B^{*}\beta \mu +\gamma \mu \right)\left(\alpha_{1}+\alpha_{2}+\alpha_{3}\right)\varrho^{\alpha_{1}+\alpha_{2}+2\alpha_{3}}\cos\left(\frac{\pi (\alpha_{1}+\alpha_{2}+\alpha_{3})}{2}+\upsilon \varrho \right)\\ \; & +\left(\mu^{2}+\varepsilon \mu +L^{*}\beta \mu -S^{*}\beta \mu +B^{*}\beta \mu +\gamma \mu \right)\left(\alpha_{1}+\alpha_{2}\right)\varrho^{\alpha_{1}+\alpha_{2}+\alpha_{3}}\left(\mu \cos\left(\frac{\pi (\alpha_{1}+\alpha_{2})}{2}+\upsilon \varrho \right)+\delta \cos\left(\frac{\pi (\alpha_{1}+\alpha_{2})}{2}\right)\right)\\ \; & +\left(\mu^{2}+\varepsilon \mu +L^{*}\beta \mu -S^{*}\beta \mu +B^{*}\beta \mu +\gamma \mu \right)\left(\alpha_{2}+\alpha_{3}\right)\left(\mu +\beta B^{*}+\beta L^{*}\right)\varrho^{\alpha_{2}+2\alpha_{3}}\cos\left(\frac{\pi (\alpha_{2}+\alpha_{3})}{2}+\upsilon \varrho \right)\\ \; & +\left(\mu^{2}+\varepsilon \mu +L^{*}\beta \mu -S^{*}\beta \mu +B^{*}\beta \mu +\gamma \mu \right)\left(\alpha_{3}+\alpha_{1}\right)\left(\varepsilon +\mu -\beta S^{*}+\gamma \right)\varrho^{2\alpha_{3}+\alpha_{1}}\cos\left(\frac{\pi (\alpha_{3}+\alpha_{1})}{2}+\upsilon \varrho \right)\\ \; & +\alpha_{1}\left(\mu^{2}+\varepsilon \mu +L^{*}\beta \mu -S^{*}\beta \mu +B^{*}\beta \mu +\gamma \mu \right)\left(\mu^{2}+\varepsilon \mu -\beta S^{*}\varepsilon -\beta S^{*}\mu +\gamma \mu \right)\varrho^{\alpha_{3}+\alpha_{1}}\cos\left(\frac{\pi \alpha_{1}}{2}+\upsilon \varrho \right)\\ \; & +\alpha_{1}\left(\mu^{2}+\varepsilon \mu +L^{*}\beta \mu -S^{*}\beta \mu +B^{*}\beta \mu +\gamma \mu \right)\left(\delta \varepsilon +\delta \mu -\beta S^{*}\delta +\delta \gamma \right)\varrho^{\alpha_{3}+\alpha_{1}}\cos\left(\frac{\pi \alpha_{1}}{2}\right)\\ \; & +\left(\mu^{2}+\varepsilon \mu +L^{*}\beta \mu -S^{*}\beta \mu +B^{*}\beta \mu +\gamma \mu \right)(\alpha_{2}\left(\mu +\beta B^{*}+\beta L^{*}\right)\varrho^{\alpha_{2}+\alpha_{3}}\left(\mu \cos\left(\frac{\pi \alpha_{2}}{2}+\upsilon \varrho \right)+\delta \cos\left(\frac{\pi \alpha_{2}}{2}\right)\right)\\ \; & +\alpha_{3}\left(\mu^{2}+\varepsilon \mu +\beta B^{*}\varepsilon +\beta B^{*}\mu +\beta L^{*}\varepsilon +\beta L^{*}\mu -\beta S^{*}\mu +\gamma \mu \right)\varrho^{2\alpha_{3}}\cos\left(\frac{\pi \alpha_{3}}{2}+\upsilon \varrho \right)). \end{array}$$

**Theorem** **2.** *Let $W_{3}$ and $W_{4}\left(\varrho ,\upsilon \right)$ be defined as in Equations (66) and (67), respectively. And let the strictly positive constants $\overset{`}{\upsilon }$ and $\overset{`}{\varrho }$ be defined respectively by*

(68) $$\overset{`}{\upsilon }=\min\left\{\upsilon \in R_{+};\;▽\left(\varrho i;0,\upsilon \right)=0\;\mathrm{holds}\;\mathrm{for}\;\mathrm{some}\;\varrho \in R_{+}\right\}$$

*and*

(69) $$\overset{`}{\varrho }=\min\left\{\varrho \in R_{+};\;▽\left(\varrho i;0,\overset{`}{\upsilon }\right)=0\right\},$$

where ▽(ξ;ϑ,υ) is defined as in Equation (16). Suppose that the conditions $W_{3}<0$ and $W_{4}\left(\overset{`}{\varrho },\overset{`}{\upsilon }\right)>0$ are satisfied. For DFSLB (3) with $\tau_{1}=0$, the endemic equilibrium $\left(S^{*},L^{*},B^{*}\right)$ is asymptotically stable whenever $0⩽\tau_{2}<\overset{`}{\upsilon }$. Moreover, at $\tau_{2}=\overset{`}{\upsilon }$, DFSLB (3) undergoes a Hopf bifurcation, giving rise to periodic solutions that branch from the same endemic equilibrium $\left(S^{*},L^{*},B^{*}\right)$.

**Proof.**  With the aid of Equation (18), we obtain

(70) $$\begin{array}{cc} ▽(\varrho i;0,\upsilon )= & \varrho^{\alpha_{1}+\alpha_{2}+\alpha_{3}}e^{\frac{\pi (\alpha_{1}+\alpha_{2}+\alpha_{3})i}{2}}+\left(\mu +\delta e^{-\upsilon \varrho i}\right)\varrho^{\alpha_{1}+\alpha_{2}}e^{\frac{\pi (\alpha_{1}+\alpha_{2})i}{2}}\\ \; & +\left(\mu +\beta B^{*}+\beta L^{*}\right)\varrho^{\alpha_{2}+\alpha_{3}}e^{\frac{\pi (\alpha_{2}+\alpha_{3})i}{2}}\\ \; & +\left(\varepsilon +\mu -\beta S^{*}+\gamma \right)\varrho^{\alpha_{3}+\alpha_{1}}e^{\frac{\pi (\alpha_{3}+\alpha_{1})i}{2}}+(\mu^{2}+\varepsilon \mu -\beta S^{*}\varepsilon -\beta S^{*}\mu \\ \; & +\gamma \mu +\delta \varepsilon e^{-\upsilon \varrho i}+\delta \mu e^{-\upsilon \varrho i}-\beta S^{*}\delta e^{-\upsilon \varrho i}+\delta \gamma e^{-\varrho \upsilon i})\varrho^{\alpha_{1}}e^{\frac{\pi \alpha_{1}i}{2}}\\ \; & +\left(\mu^{2}+\beta B^{*}\mu +\beta L^{*}\mu +\delta \mu e^{-\upsilon \varrho i}+\beta B^{*}\delta e^{-\upsilon \varrho i}+\beta L^{*}\delta e^{-\upsilon \varrho i}\right)\varrho^{\alpha_{2}}e^{\frac{\pi \alpha_{2}i}{2}}\\ \; & +\left(\mu^{2}+\varepsilon \mu +\beta B^{*}\varepsilon +\beta B^{*}\mu +\beta L^{*}\varepsilon +\beta L^{*}\mu -\beta S^{*}\mu +\gamma \mu \right)\varrho^{\alpha_{3}}e^{\frac{\pi \alpha_{3}i}{2}}\\ \; & +\mu^{3}+\varepsilon \mu^{2}+B^{*}\beta \mu^{2}+L^{*}\beta \mu^{2}-S^{*}\beta \mu^{2}+B^{*}\beta \varepsilon \mu +L^{*}\beta \varepsilon \mu -S^{*}\beta \varepsilon \mu \\ \; & +\gamma \mu^{2}+\delta \mu^{2}e^{-\upsilon \varrho i}+\delta \varepsilon \mu e^{-\upsilon \varrho i}+L^{*}\beta \delta \mu e^{-\upsilon \varrho i}-S^{*}\beta \delta \mu e^{-\upsilon \varrho i}\\ \; & +B^{*}\beta \delta \mu e^{-\upsilon \varrho i}+\delta \gamma \mu e^{-\varrho \upsilon i},\;\forall \varrho ,\upsilon \in \left[0,+\infty \right). \end{array}$$

By routine calculations, we can rewrite ▽(ϱi;0,υ) defined in Equation (70) as

(71) $$▽\left(\varrho i;0,\upsilon \right)=H_{08}\left(\varrho \right)+H_{09}\left(\varrho \right)e^{-\varrho \upsilon i},$$

where $H_{08}\left(\varrho \right)$ and $H_{09}\left(\varrho \right)$ are, respectively, defined as

(72) $$\begin{array}{cc} H_{08}\left(\varrho \right)= & \varrho^{\alpha_{1}+\alpha_{2}+\alpha_{3}}e^{\frac{\pi (\alpha_{1}+\alpha_{2}+\alpha_{3})i}{2}}+\mu \varrho^{\alpha_{1}+\alpha_{2}}e^{\frac{\pi (\alpha_{1}+\alpha_{2})i}{2}}\\ \; & +\left(\mu +\beta B^{*}+\beta L^{*}\right)\varrho^{\alpha_{2}+\alpha_{3}}e^{\frac{\pi (\alpha_{2}+\alpha_{3})i}{2}}+\left(\varepsilon +\mu -\beta S^{*}+\gamma \right)\varrho^{\alpha_{3}+\alpha_{1}}e^{\frac{\pi (\alpha_{3}+\alpha_{1})i}{2}}\\ \; & +\left(\mu^{2}+\varepsilon \mu -\beta S^{*}\varepsilon -\beta S^{*}\mu +\gamma \mu \right)\varrho^{\alpha_{1}}e^{\frac{\pi \alpha_{1}i}{2}}+\left(\mu^{2}+\beta B^{*}\mu +\beta L^{*}\mu \right)\varrho^{\alpha_{2}}e^{\frac{\pi \alpha_{2}i}{2}}\\ \; & +\left(\mu^{2}+\varepsilon \mu +\beta B^{*}\varepsilon +\beta B^{*}\mu +\beta L^{*}\varepsilon +\beta L^{*}\mu -\beta S^{*}\mu +\gamma \mu \right)\varrho^{\alpha_{3}}e^{\frac{\pi \alpha_{3}i}{2}}\\ \; & +\mu^{3}+\varepsilon \mu^{2}+B^{*}\beta \mu^{2}+L^{*}\beta \mu^{2}-S^{*}\beta \mu^{2}+B^{*}\beta \varepsilon \mu +L^{*}\beta \varepsilon \mu -S^{*}\beta \varepsilon \mu +\gamma \mu^{2} \end{array}$$

and

(73) $$\begin{array}{cc} H_{09}\left(\varrho \right)= & \delta \varrho^{\alpha_{1}+\alpha_{2}}e^{\frac{\pi (\alpha_{1}+\alpha_{2})i}{2}}+\left(\delta \varepsilon +\delta \mu -\beta S^{*}\delta +\delta \gamma \right)\varrho^{\alpha_{1}}e^{\frac{\pi \alpha_{1}i}{2}}\\ \; & +\left(\delta \mu +\beta B^{*}\delta +\beta L^{*}\delta \right)\varrho^{\alpha_{2}}e^{\frac{\pi \alpha_{2}i}{2}}+\delta \mu^{2}+\delta \varepsilon \mu +L^{*}\beta \delta \mu \\ \; & -S^{*}\beta \delta \mu +B^{*}\beta \delta \mu +\delta \gamma \mu . \end{array}$$

With the aid of Equation (71), we conclude that a necessary and sufficient condition for $▽(\overset{`}{\varrho }i;0,\overset{`}{\upsilon })=0$ is given by

(74) $$H_{08}\left(\overset{`}{\varrho }\right)+H_{09}\left(\overset{`}{\varrho }\right)e^{-\overset{`}{\varrho }\overset{`}{\upsilon }i}=0,$$

which necessitates the following condition

(75) $$\Omega_{2}\left(\overset{`}{\varrho }\right)=|H_{08}\left(\overset{`}{\varrho }\right){|}^{2}-{\left|H_{09}\left(\overset{`}{\varrho }\right)\right|}^{2}=0.$$

Motivated by this, and following straightforward calculations, we have

(76) $$\begin{array}{cc} \Omega_{2}\left(\varrho \right)= & |H_{08}{\left(\varrho \right)|}^{2}-{\left|H_{09}\left(\varrho \right)\right|}^{2}\\ = & \varrho^{2(\alpha_{1}+\alpha_{2}+\alpha_{3})}+\sum_{j=1}^{5}H_{10j}\left(\varrho \right)+W_{3}, \end{array}$$

in which, $W_{3}$, $H_{101}\left(\varrho \right)$, $H_{102}\left(\varrho \right)$, $H_{103}\left(\varrho \right)$, $H_{104}\left(\varrho \right)$, and $H_{105}\left(\varrho \right)$ are, respectively, defined in Equation (66) and by

(77) $$\begin{array}{cc} H_{101}\left(\varrho \right)=\; & 2(\mu^{3}+\varepsilon \mu^{2}+B^{*}\beta \mu^{2}+L^{*}\beta \mu^{2}-S^{*}\beta \mu^{2}+B^{*}\beta \varepsilon \mu +L^{*}\beta \varepsilon \mu -S^{*}\beta \varepsilon \mu \\ \; & \;+\gamma \mu^{2})\left(\mu^{2}+\varepsilon \mu -\beta S^{*}\varepsilon -\beta S^{*}\mu +\gamma \mu \right)\varrho^{\alpha_{1}}\cos\left(\frac{\pi \alpha_{1}}{2}\right)\\ \; & +2(\mu^{3}+\varepsilon \mu^{2}+B^{*}\beta \mu^{2}+L^{*}\beta \mu^{2}-S^{*}\beta \mu^{2}+B^{*}\beta \varepsilon \mu +L^{*}\beta \varepsilon \mu -S^{*}\beta \varepsilon \mu \\ \; & \;+\gamma \mu^{2})\left(\mu^{2}+\beta B^{*}\mu +\beta L^{*}\mu \right)\varrho^{\alpha_{2}}\cos\left(\frac{\pi \alpha_{2}}{2}\right)\\ \; & +2(\mu^{3}+\varepsilon \mu^{2}+B^{*}\beta \mu^{2}+L^{*}\beta \mu^{2}-S^{*}\beta \mu^{2}+B^{*}\beta \varepsilon \mu +L^{*}\beta \varepsilon \mu -S^{*}\beta \varepsilon \mu \\ \; & \;+\gamma \mu^{2})(\mu^{2}+\varepsilon \mu +\beta B^{*}\varepsilon +\beta B^{*}\mu +\beta L^{*}\varepsilon +\beta L^{*}\mu -\beta S^{*}\mu \\ \; & +\gamma \mu )\varrho^{\alpha_{3}}\cos\left(\frac{\pi \alpha_{3}}{2}\right)-2(\delta \mu^{2}+\delta \varepsilon \mu +L^{*}\beta \delta \mu -S^{*}\beta \delta \mu +B^{*}\beta \delta \mu \\ \; & \;\;\;\;\;\;+\delta \gamma \mu )\left(\delta \varepsilon +\delta \mu -\beta S^{*}\delta +\delta \gamma \right)\varrho^{\alpha_{1}}\cos\left(\frac{\pi \alpha_{1}}{2}\right)\\ \; & -2(\delta \mu^{2}+\delta \varepsilon \mu +L^{*}\beta \delta \mu -S^{*}\beta \delta \mu +B^{*}\beta \delta \mu \\ \; & \;+\delta \gamma \mu )\left(\delta \mu +\beta B^{*}\delta +\beta L^{*}\delta \right)\varrho^{\alpha_{2}}\cos\left(\frac{\pi \alpha_{2}}{2}\right), \end{array}$$

(78) $$\begin{array}{cc} H_{102}\left(\varrho \right)= & 2\mu (\mu^{3}+\varepsilon \mu^{2}+B^{*}\beta \mu^{2}+L^{*}\beta \mu^{2}-S^{*}\beta \mu^{2}+B^{*}\beta \varepsilon \mu \\ \; & +L^{*}\beta \varepsilon \mu -S^{*}\beta \varepsilon \mu +\gamma \mu^{2})\varrho^{\alpha_{1}+\alpha_{2}}\cos\left(\frac{\pi (\alpha_{1}+\alpha_{2})}{2}\right)\\ \; & +2(\mu^{3}+\varepsilon \mu^{2}+B^{*}\beta \mu^{2}+L^{*}\beta \mu^{2}-S^{*}\beta \mu^{2}+B^{*}\beta \varepsilon \mu +L^{*}\beta \varepsilon \mu -S^{*}\beta \varepsilon \mu \\ \; & \;+\gamma \mu^{2})\left(\mu +\beta B^{*}+\beta L^{*}\right)\varrho^{\alpha_{2}+\alpha_{3}}\cos\left(\frac{\pi (\alpha_{2}+\alpha_{3})}{2}\right)\\ \; & +2(\mu^{3}+\varepsilon \mu^{2}+B^{*}\beta \mu^{2}+L^{*}\beta \mu^{2}-S^{*}\beta \mu^{2}+B^{*}\beta \varepsilon \mu +L^{*}\beta \varepsilon \mu -S^{*}\beta \varepsilon \mu \\ \; & \;+\gamma \mu^{2})\left(\varepsilon +\mu -\beta S^{*}+\gamma \right)\varrho^{\alpha_{3}+\alpha_{1}}\cos\left(\frac{\pi (\alpha_{3}+\alpha_{1})}{2}\right)\\ \; & +{\left(\mu^{2}+\varepsilon \mu -\beta S^{*}\varepsilon -\beta S^{*}\mu +\gamma \mu \right)}^{2}\varrho^{2\alpha_{1}}+{\left(\mu^{2}+\beta B^{*}\mu +\beta L^{*}\mu \right)}^{2}\varrho^{2\alpha_{2}}\\ \; & +{\left(\mu^{2}+\varepsilon \mu +\beta B^{*}\varepsilon +\beta B^{*}\mu +\beta L^{*}\varepsilon +\beta L^{*}\mu -\beta S^{*}\mu +\gamma \mu \right)}^{2}\varrho^{2\alpha_{3}}\\ \; & +2\left(\mu^{2}+\varepsilon \mu -\beta S^{*}\varepsilon -\beta S^{*}\mu +\gamma \mu \right)\left(\mu^{2}+\beta B^{*}\mu +\beta L^{*}\mu \right)\varrho^{\alpha_{1}+\alpha_{2}}\cos\left(\frac{\pi (\alpha_{2}-\alpha_{1})}{2}\right)\\ \; & +2\left(\mu^{2}+\varepsilon \mu +\beta B^{*}\varepsilon +\beta B^{*}\mu +\beta L^{*}\varepsilon +\beta L^{*}\mu -\beta S^{*}\mu +\gamma \mu \right)(\mu^{2}+\varepsilon \mu -\beta S^{*}\varepsilon -\beta S^{*}\mu \\ \; & \;+\gamma \mu )\varrho^{\alpha_{3}+\alpha_{1}}\cos\left(\frac{\pi (\alpha_{1}-\alpha_{3})}{2}\right)+2\left(\mu^{2}+\beta B^{*}\mu +\beta L^{*}\mu \right)(\mu^{2}+\varepsilon \mu +\beta B^{*}\varepsilon \\ \; & +\beta B^{*}\mu +\beta L^{*}\varepsilon +\beta L^{*}\mu -\beta S^{*}\mu +\gamma \mu )\varrho^{\alpha_{2}+\alpha_{3}}\cos\left(\frac{\pi (\alpha_{3}-\alpha_{2})}{2}\right)\\ \; & -2\delta \left(\delta \mu^{2}+\delta \varepsilon \mu +L^{*}\beta \delta \mu -S^{*}\beta \delta \mu +B^{*}\beta \delta \mu +\delta \gamma \mu \right)\varrho^{\alpha_{1}+\alpha_{2}}\cos\left(\frac{\pi (\alpha_{1}+\alpha_{2})}{2}\right)\\ \; & -{\left(\delta \varepsilon +\delta \mu -\beta S^{*}\delta +\delta \gamma \right)}^{2}\varrho^{2\alpha_{1}}-{\left(\delta \mu +\beta B^{*}\delta +\beta L^{*}\delta \right)}^{2}\varrho^{2\alpha_{2}}\\ \; & -2\left(\delta \varepsilon +\delta \mu -\beta S^{*}\delta +\delta \gamma \right)\left(\delta \mu +\beta B^{*}\delta +\beta L^{*}\delta \right)\varrho^{\alpha_{1}+\alpha_{2}}\cos\left(\frac{\pi (\alpha_{2}-\alpha_{1})}{2}\right), \end{array}$$

(79) $$\begin{array}{cc} H_{103}\left(\varrho \right)= & 2\left(\delta \mu^{2}+\delta \varepsilon \mu +L^{*}\beta \delta \mu -S^{*}\beta \delta \mu +B^{*}\beta \delta \mu +\delta \gamma \mu \right)\varrho^{\alpha_{1}+\alpha_{2}+\alpha_{3}}\cos\left(\frac{\pi (\alpha_{1}+\alpha_{2}+\alpha_{3})}{2}\right)\\ \; & +2\mu \left(\mu^{2}+\varepsilon \mu -\beta S^{*}\varepsilon -\beta S^{*}\mu +\gamma \mu \right)\varrho^{2\alpha_{1}+\alpha_{2}}\cos\left(\frac{\pi \alpha_{2}}{2}\right)\\ \; & +2\mu \left(\mu^{2}+\beta B^{*}\mu +\beta L^{*}\mu \right)\varrho^{\alpha_{1}+2\alpha_{2}}\cos\left(\frac{\pi \alpha_{1}}{2}\right)\\ \; & +2\mu \left(\mu^{2}+\varepsilon \mu +\beta B^{*}\varepsilon +\beta B^{*}\mu +\beta L^{*}\varepsilon +\beta L^{*}\mu -\beta S^{*}\mu +\gamma \mu \right)\varrho^{\alpha_{1}+\alpha_{2}+\alpha_{3}}\cos\left(\frac{\pi (\alpha_{1}+\alpha_{2}-\alpha_{3})}{2}\right)\\ \; & +2\left(\mu^{2}+\varepsilon \mu -\beta S^{*}\varepsilon -\beta S^{*}\mu +\gamma \mu \right)\left(\mu +\beta B^{*}+\beta L^{*}\right)\varrho^{\alpha_{1}+\alpha_{2}+\alpha_{3}}\cos\left(\frac{\pi (\alpha_{2}+\alpha_{3}-\alpha_{1})}{2}\right)\\ \; & +2\left(\mu^{2}+\beta B^{*}\mu +\beta L^{*}\mu \right)\left(\mu +\beta B^{*}+\beta L^{*}\right)\varrho^{2\alpha_{2}+\alpha_{3}}\cos\left(\frac{\pi \alpha_{3}}{2}\right)\\ \; & +2\left(\mu^{2}+\varepsilon \mu +\beta B^{*}\varepsilon +\beta B^{*}\mu +\beta L^{*}\varepsilon +\beta L^{*}\mu -\beta S^{*}\mu +\gamma \mu \right)\left(\mu +\beta B^{*}+\beta L^{*}\right)\varrho^{\alpha_{2}+2\alpha_{3}}\cos\left(\frac{\pi \alpha_{2}}{2}\right)\\ \; & +2\left(\mu^{2}+\varepsilon \mu -\beta S^{*}\varepsilon -\beta S^{*}\mu +\gamma \mu \right)\left(\varepsilon +\mu -\beta S^{*}+\gamma \right)\varrho^{2\alpha_{1}+\alpha_{3}}\cos\left(\frac{\pi \alpha_{3}}{2}\right)\\ \; & +2\left(\mu^{2}+\beta B^{*}\mu +\beta L^{*}\mu \right)\left(\varepsilon +\mu -\beta S^{*}+\gamma \right)\varrho^{\alpha_{1}+\alpha_{2}+\alpha_{3}}\cos\left(\frac{\pi (\alpha_{1}-\alpha_{2}+\alpha_{3})}{2}\right)\\ \; & +2\left(\mu^{2}+\varepsilon \mu +\beta B^{*}\varepsilon +\beta B^{*}\mu +\beta L^{*}\varepsilon +\beta L^{*}\mu -\beta S^{*}\mu +\gamma \mu \right)\left(\varepsilon +\mu -\beta S^{*}+\gamma \right)\varrho^{\alpha_{1}+2\alpha_{3}}\cos\left(\frac{\pi \alpha_{1}}{2}\right)\\ \; & -2\delta \left(\delta \varepsilon +\delta \mu -\beta S^{*}\delta +\delta \gamma \right)\varrho^{2\alpha_{1}+\alpha_{2}}\cos\left(\frac{\pi \alpha_{2}}{2}\right)-2\delta \left(\delta \mu +\beta B^{*}\delta +\beta L^{*}\delta \right)\varrho^{\alpha_{1}+2\alpha_{2}}\cos\left(\frac{\pi \alpha_{1}}{2}\right), \end{array}$$

(80) $$\begin{array}{cc} H_{104}\left(\varrho \right)= & \mu^{2}\varrho^{2\alpha_{1}+2\alpha_{2}}+{\left(\mu +\beta B^{*}+\beta L^{*}\right)}^{2}\varrho^{2\alpha_{2}+2\alpha_{3}}+{\left(\varepsilon +\mu -\beta S^{*}+\gamma \right)}^{2}\varrho^{2\alpha_{3}+2\alpha_{1}}\\ \; & +2\left(\mu^{2}+\varepsilon \mu -\beta S^{*}\varepsilon -\beta S^{*}\mu +\gamma \mu \right)\varrho^{2\alpha_{1}+\alpha_{2}+\alpha_{3}}\cos\left(\frac{\pi (\alpha_{2}+\alpha_{3})}{2}\right)\\ \; & +2\left(\mu^{2}+\beta B^{*}\mu +\beta L^{*}\mu \right)\varrho^{\alpha_{1}+2\alpha_{2}+\alpha_{3}}\cos\left(\frac{\pi (\alpha_{3}+\alpha_{1})}{2}\right)\\ \; & +2\left(\mu^{2}+\varepsilon \mu +\beta B^{*}\varepsilon +\beta B^{*}\mu +\beta L^{*}\varepsilon +\beta L^{*}\mu -\beta S^{*}\mu +\gamma \mu \right)\varrho^{\alpha_{1}+\alpha_{2}+2\alpha_{3}}\cos\left(\frac{\pi (\alpha_{1}+\alpha_{2})}{2}\right)\\ \; & +2\mu \left(\mu +\beta B^{*}+\beta L^{*}\right)\varrho^{\alpha_{1}+2\alpha_{2}+\alpha_{3}}\cos\left(\frac{\pi (\alpha_{1}-\alpha_{3})}{2}\right)\\ \; & +2\mu \left(\varepsilon +\mu -\beta S^{*}+\gamma \right)\varrho^{2\alpha_{1}+\alpha_{2}+\alpha_{3}}\cos\left(\frac{\pi (\alpha_{3}-\alpha_{2})}{2}\right)\\ \; & +2\left(\mu +\beta B^{*}+\beta L^{*}\right)\left(\varepsilon +\mu -\beta S^{*}+\gamma \right)\varrho^{\alpha_{1}+\alpha_{2}+2\alpha_{3}}\cos\left(\frac{\pi (\alpha_{2}-\alpha_{1})}{2}\right)-\delta^{2}\varrho^{2\alpha_{1}+2\alpha_{2}}, \end{array}$$

and by

(81) $$\begin{array}{cc} H_{105}\left(\varrho \right)= & 2\mu \varrho^{2\alpha_{1}+2\alpha_{2}+\alpha_{3}}\cos\left(\frac{\pi \alpha_{3}}{2}\right)+2\left(\mu +\beta B^{*}+\beta L^{*}\right)\varrho^{\alpha_{1}+2\alpha_{2}+2\alpha_{3}}\cos\left(\frac{\pi \alpha_{1}}{2}\right)\\ \; & +2\left(\varepsilon +\mu -\beta S^{*}+\gamma \right)\varrho^{2\alpha_{1}+\alpha_{2}+2\alpha_{3}}\cos\left(\frac{\pi \alpha_{2}}{2}\right). \end{array}$$

By virtue of Equations (77)–(81), it follows that

(82) $$H_{10j}\left(0\right)=0,\;j=1,2,3,4,5.$$

Together with (76), this immediately implies $\Omega_{2}\left(0\right)=W_{3}<0$, where the constant $W_{3}$ is defined in Equation (66). Moreover, one readily verifies that

(83) $$\lim_{\varrho \to +\infty }\Omega_{2}\left(\varrho \right)=+\infty .$$

By the limit properties of functions, there exists ${\hat{\varrho }}_{2}\in \left(0,+\infty \right)$ such that $\Omega_{2}\left({\hat{\varrho }}_{2}\right)>0$. Applying the intermediate value theorem to the continuous function $\Omega_{2}\left(\varrho \right)$ then yields a constant $\varrho_{2}\in \left(0,{\hat{\varrho }}_{2}\right)$ satisfying $\Omega_{2}\left(\varrho_{2}\right)=0$. Consider now the following algebraic equation in the unknown $\upsilon_{2}\in \left(0,+\infty \right)$:

(84) $$▽(\varrho_{2}i;0,\upsilon_{2})=0,$$

where ▽(ϱi;0,υ) is given as in Equation (70). With the aid of Equation (71), Equation (84) can be recast into the following equivalent form

(85) $$\begin{array}{cc} \cos\left(\varrho_{2}\upsilon_{2}\right)+i\sin\left(\varrho_{2}\upsilon_{2}\right)= & e^{\varrho_{2}\upsilon_{2}i}=-\frac{H_{09}\left(\varrho_{2}\right)}{H_{08}\left(\varrho_{2}\right)}\\ = & -\frac{\bar{H_{08}\left(\varrho_{2}\right)}H_{09}\left(\varrho_{2}\right)}{|H_{08}\left(\varrho_{2}\right){|}^{2}}=-\frac{\bar{H_{08}\left(\varrho_{2}\right)}H_{09}\left(\varrho_{2}\right)}{|H_{09}\left(\varrho_{2}\right){|}^{2}}\\ = & -\frac{H_{11R}\left(\varrho_{2}\right)}{|H_{09}\left(\varrho_{2}\right){|}^{2}}-i\frac{H_{11I}\left(\varrho_{2}\right)}{|H_{09}\left(\varrho_{2}\right){|}^{2}}, \end{array}$$

in which $|H_{09}{\left(\varrho \right)|}^{2}$ can be simplified to

(86) $$\begin{array}{cc} |H_{09}{\left(\varrho \right)|}^{2}= & \delta^{2}\varrho^{2\alpha_{1}+2\alpha_{2}}+2\delta \left(\delta \varepsilon +\delta \mu -\beta S^{*}\delta +\delta \gamma \right)\varrho^{2\alpha_{1}+\alpha_{2}}\cos\left(\frac{\pi \alpha_{2}}{2}\right)+2\delta \left(\delta \mu +\beta B^{*}\delta +\beta L^{*}\delta \right)\varrho^{\alpha_{1}+2\alpha_{2}}\cos\left(\frac{\pi \alpha_{1}}{2}\right)\\ \; & +2\delta \left(\delta \mu^{2}+\delta \varepsilon \mu +L^{*}\beta \delta \mu -S^{*}\beta \delta \mu +B^{*}\beta \delta \mu +\delta \gamma \mu \right)\varrho^{\alpha_{1}+\alpha_{2}}\cos\left(\frac{\pi (\alpha_{1}+\alpha_{2})}{2}\right)\\ \; & +{\left(\delta \varepsilon +\delta \mu -\beta S^{*}\delta +\delta \gamma \right)}^{2}\varrho^{2\alpha_{1}}+{\left(\delta \mu +\beta B^{*}\delta +\beta L^{*}\delta \right)}^{2}\varrho^{2\alpha_{2}}\\ \; & +2\left(\delta \varepsilon +\delta \mu -\beta S^{*}\delta +\delta \gamma \right)\left(\delta \mu +\beta B^{*}\delta +\beta L^{*}\delta \right)\varrho^{\alpha_{1}+\alpha_{2}}\cos\left(\frac{\pi (\alpha_{2}-\alpha_{1})}{2}\right)\\ \; & +2\left(\delta \mu^{2}+\delta \varepsilon \mu +L^{*}\beta \delta \mu -S^{*}\beta \delta \mu +B^{*}\beta \delta \mu +\delta \gamma \mu \right)\left(\delta \varepsilon +\delta \mu -\beta S^{*}\delta +\delta \gamma \right)\varrho^{\alpha_{1}}\cos\left(\frac{\pi \alpha_{1}}{2}\right)\\ \; & +2\left(\delta \mu^{2}+\delta \varepsilon \mu +L^{*}\beta \delta \mu -S^{*}\beta \delta \mu +B^{*}\beta \delta \mu +\delta \gamma \mu \right)\left(\delta \mu +\beta B^{*}\delta +\beta L^{*}\delta \right)\varrho^{\alpha_{2}}\cos\left(\frac{\pi \alpha_{2}}{2}\right)\\ \; & +{\left(\delta \mu^{2}+\delta \varepsilon \mu +L^{*}\beta \delta \mu -S^{*}\beta \delta \mu +B^{*}\beta \delta \mu +\delta \gamma \mu \right)}^{2}. \end{array}$$

In Equation (85), $H_{11R}\left(\varrho \right)$ and $H_{11I}\left(\varrho \right)$ are given respectively by

(87) $$\begin{array}{cc} H_{11R}\left(\varrho \right)= & \delta \varrho^{2\alpha_{1}+2\alpha_{2}+\alpha_{3}}\cos\left(\frac{\pi \alpha_{3}}{2}\right)+\sum_{j=1}^{4}H_{11Rj}\left(\varrho \right)\\ \; & +(\mu^{3}+\varepsilon \mu^{2}+B^{*}\beta \mu^{2}+L^{*}\beta \mu^{2}-S^{*}\beta \mu^{2}+B^{*}\beta \varepsilon \mu +L^{*}\beta \varepsilon \mu -S^{*}\beta \varepsilon \mu \\ \; & \;+\gamma \mu^{2})\left(\delta \mu^{2}+\delta \varepsilon \mu +L^{*}\beta \delta \mu -S^{*}\beta \delta \mu +B^{*}\beta \delta \mu +\delta \gamma \mu \right) \end{array}$$

and

(88) $$H_{11I}\left(\varrho \right)=-\delta \varrho^{2\alpha_{1}+2\alpha_{2}+\alpha_{3}}\sin\left(\frac{\pi \alpha_{3}}{2}\right)+\sum_{j=1}^{4}H_{11Ij}\left(\varrho \right).$$

In Equations (87) and (88), the terms $H_{11R1}\left(\varrho \right)$, $H_{11I1}\left(\varrho \right)$, $H_{11R2}\left(\varrho \right)$, $H_{11I2}\left(\varrho \right)$, $H_{11R3}\left(\varrho \right)$, $H_{11I3}\left(\varrho \right)$, $H_{11R4}\left(\varrho \right)$, and $H_{11I4}\left(\varrho \right)$ are given respectively by

(89) $$\begin{array}{cc} H_{11R1}\left(\varrho \right)=\; & \left(\delta \mu^{2}+\delta \varepsilon \mu +L^{*}\beta \delta \mu -S^{*}\beta \delta \mu +B^{*}\beta \delta \mu +\delta \gamma \mu \right)\left(\mu^{2}+\varepsilon \mu -\beta S^{*}\varepsilon -\beta S^{*}\mu +\gamma \mu \right)\varrho^{\alpha_{1}}\cos\left(\frac{\pi \alpha_{1}}{2}\right)\\ \; & +\left(\delta \mu^{2}+\delta \varepsilon \mu +L^{*}\beta \delta \mu -S^{*}\beta \delta \mu +B^{*}\beta \delta \mu +\delta \gamma \mu \right)\left(\mu^{2}+\beta B^{*}\mu +\beta L^{*}\mu \right)\varrho^{\alpha_{2}}\cos\left(\frac{\pi \alpha_{2}}{2}\right)\\ \; & +\left(\delta \mu^{2}+\delta \varepsilon \mu +L^{*}\beta \delta \mu -S^{*}\beta \delta \mu +B^{*}\beta \delta \mu +\delta \gamma \mu \right)(\mu^{2}+\varepsilon \mu +\beta B^{*}\varepsilon +\beta B^{*}\mu +\beta L^{*}\varepsilon +\beta L^{*}\mu -\beta S^{*}\mu \\ \; & +\gamma \mu )\varrho^{\alpha_{3}}\cos\left(\frac{\pi \alpha_{3}}{2}\right)+(\mu^{3}+\varepsilon \mu^{2}+B^{*}\beta \mu^{2}+L^{*}\beta \mu^{2}-S^{*}\beta \mu^{2}+B^{*}\beta \varepsilon \mu +L^{*}\beta \varepsilon \mu -S^{*}\beta \varepsilon \mu \\ \; & +\gamma \mu^{2})\left(\delta \varepsilon +\delta \mu -\beta S^{*}\delta +\delta \gamma \right)\varrho^{\alpha_{1}}\cos\left(\frac{\pi \alpha_{1}}{2}\right)+(\mu^{3}+\varepsilon \mu^{2}+B^{*}\beta \mu^{2}+L^{*}\beta \mu^{2}-S^{*}\beta \mu^{2}+B^{*}\beta \varepsilon \mu \\ \; & +L^{*}\beta \varepsilon \mu -S^{*}\beta \varepsilon \mu +\gamma \mu^{2})\left(\delta \mu +\beta B^{*}\delta +\beta L^{*}\delta \right)\varrho^{\alpha_{2}}\cos\left(\frac{\pi \alpha_{2}}{2}\right), \end{array}$$

(90) $$\begin{array}{cc} H_{11I1}\left(\varrho \right)=\; & -\left(\delta \mu^{2}+\delta \varepsilon \mu +L^{*}\beta \delta \mu -S^{*}\beta \delta \mu +B^{*}\beta \delta \mu +\delta \gamma \mu \right)\left(\mu^{2}+\varepsilon \mu -\beta S^{*}\varepsilon -\beta S^{*}\mu +\gamma \mu \right)\varrho^{\alpha_{1}}\sin\left(\frac{\pi \alpha_{1}}{2}\right)\\ \; & -\left(\delta \mu^{2}+\delta \varepsilon \mu +L^{*}\beta \delta \mu -S^{*}\beta \delta \mu +B^{*}\beta \delta \mu +\delta \gamma \mu \right)\left(\mu^{2}+\beta B^{*}\mu +\beta L^{*}\mu \right)\varrho^{\alpha_{2}}\sin\left(\frac{\pi \alpha_{2}}{2}\right)\\ \; & -\left(\delta \mu^{2}+\delta \varepsilon \mu +L^{*}\beta \delta \mu -S^{*}\beta \delta \mu +B^{*}\beta \delta \mu +\delta \gamma \mu \right)(\mu^{2}+\varepsilon \mu +\beta B^{*}\varepsilon +\beta B^{*}\mu +\beta L^{*}\varepsilon +\beta L^{*}\mu -\beta S^{*}\mu \\ \; & +\gamma \mu )\varrho^{\alpha_{3}}\sin\left(\frac{\pi \alpha_{3}}{2}\right)+\left(\mu^{3}+\varepsilon \mu^{2}+B^{*}\beta \mu^{2}+L^{*}\beta \mu^{2}-S^{*}\beta \mu^{2}+B^{*}\beta \varepsilon \mu +L^{*}\beta \varepsilon \mu -S^{*}\beta \varepsilon \mu +\gamma \mu^{2}\right)(\delta \varepsilon \\ \; & +\delta \mu -\beta S^{*}\delta +\delta \gamma )\varrho^{\alpha_{1}}\sin\left(\frac{\pi \alpha_{1}}{2}\right)+(\mu^{3}+\varepsilon \mu^{2}+B^{*}\beta \mu^{2}+L^{*}\beta \mu^{2}-S^{*}\beta \mu^{2}+B^{*}\beta \varepsilon \mu \\ \; & +L^{*}\beta \varepsilon \mu -S^{*}\beta \varepsilon \mu +\gamma \mu^{2})\left(\delta \mu +\beta B^{*}\delta +\beta L^{*}\delta \right)\varrho^{\alpha_{2}}\sin\left(\frac{\pi \alpha_{2}}{2}\right), \end{array}$$

(91) $$\begin{array}{cc} H_{11R2}\left(\varrho \right)= & \mu \left(\delta \mu^{2}+\delta \varepsilon \mu +L^{*}\beta \delta \mu -S^{*}\beta \delta \mu +B^{*}\beta \delta \mu +\delta \gamma \mu \right)\varrho^{\alpha_{1}+\alpha_{2}}\cos\left(\frac{\pi (\alpha_{1}+\alpha_{2})}{2}\right)\\ \; & +\left(\delta \mu^{2}+\delta \varepsilon \mu +L^{*}\beta \delta \mu -S^{*}\beta \delta \mu +B^{*}\beta \delta \mu +\delta \gamma \mu \right)\left(\mu +\beta B^{*}+\beta L^{*}\right)\varrho^{\alpha_{2}+\alpha_{3}}\cos\left(\frac{\pi (\alpha_{2}+\alpha_{3})}{2}\right)\\ \; & +\left(\delta \mu^{2}+\delta \varepsilon \mu +L^{*}\beta \delta \mu -S^{*}\beta \delta \mu +B^{*}\beta \delta \mu +\delta \gamma \mu \right)\left(\varepsilon +\mu -\beta S^{*}+\gamma \right)\varrho^{\alpha_{3}+\alpha_{1}}\cos\left(\frac{\pi (\alpha_{3}+\alpha_{1})}{2}\right)\\ \; & +\left(\delta \varepsilon +\delta \mu -\beta S^{*}\delta +\delta \gamma \right)\left(\mu^{2}+\varepsilon \mu -\beta S^{*}\varepsilon -\beta S^{*}\mu +\gamma \mu \right)\varrho^{2\alpha_{1}}\\ \; & +\left(\delta \varepsilon +\delta \mu -\beta S^{*}\delta +\delta \gamma \right)\left(\mu^{2}+\beta B^{*}\mu +\beta L^{*}\mu \right)\varrho^{\alpha_{1}+\alpha_{2}}\cos\left(\frac{\pi (\alpha_{2}-\alpha_{1})}{2}\right)\\ \; & +\left(\delta \varepsilon +\delta \mu -\beta S^{*}\delta +\delta \gamma \right)(\mu^{2}+\varepsilon \mu +\beta B^{*}\varepsilon +\beta B^{*}\mu +\beta L^{*}\varepsilon \\ \; & +\beta L^{*}\mu -\beta S^{*}\mu +\gamma \mu )\varrho^{\alpha_{3}+\alpha_{1}}\cos\left(\frac{\pi (\alpha_{1}-\alpha_{3})}{2}\right)\\ \; & +\left(\delta \mu +\beta B^{*}\delta +\beta L^{*}\delta \right)\left(\mu^{2}+\varepsilon \mu -\beta S^{*}\varepsilon -\beta S^{*}\mu +\gamma \mu \right)\varrho^{\alpha_{1}+\alpha_{2}}\cos\left(\frac{\pi (\alpha_{2}-\alpha_{1})}{2}\right)\\ \; & +\left(\delta \mu +\beta B^{*}\delta +\beta L^{*}\delta \right)\left(\mu^{2}+\beta B^{*}\mu +\beta L^{*}\mu \right)\varrho^{2\alpha_{2}}\\ \; & +\left(\delta \mu +\beta B^{*}\delta +\beta L^{*}\delta \right)(\mu^{2}+\varepsilon \mu +\beta B^{*}\varepsilon +\beta B^{*}\mu +\beta L^{*}\varepsilon \\ \; & +\beta L^{*}\mu -\beta S^{*}\mu +\gamma \mu )\varrho^{\alpha_{2}+\alpha_{3}}\cos\left(\frac{\pi (\alpha_{3}-\alpha_{2})}{2}\right)\\ \; & +\delta (\mu^{3}+\varepsilon \mu^{2}+B^{*}\beta \mu^{2}+L^{*}\beta \mu^{2}-S^{*}\beta \mu^{2}+B^{*}\beta \varepsilon \mu \\ \; & +L^{*}\beta \varepsilon \mu -S^{*}\beta \varepsilon \mu +\gamma \mu^{2})\varrho^{\alpha_{1}+\alpha_{2}}\cos\left(\frac{\pi (\alpha_{1}+\alpha_{2})}{2}\right), \end{array}$$

(92) $$\begin{array}{cc} H_{11I2}\left(\varrho \right)= & -\mu \left(\delta \mu^{2}+\delta \varepsilon \mu +L^{*}\beta \delta \mu -S^{*}\beta \delta \mu +B^{*}\beta \delta \mu +\delta \gamma \mu \right)\varrho^{\alpha_{1}+\alpha_{2}}\sin\left(\frac{\pi (\alpha_{1}+\alpha_{2})}{2}\right)\\ \; & -\left(\delta \mu^{2}+\delta \varepsilon \mu +L^{*}\beta \delta \mu -S^{*}\beta \delta \mu +B^{*}\beta \delta \mu +\delta \gamma \mu \right)\left(\mu +\beta B^{*}+\beta L^{*}\right)\varrho^{\alpha_{2}+\alpha_{3}}\sin\left(\frac{\pi (\alpha_{2}+\alpha_{3})}{2}\right)\\ \; & -\left(\delta \mu^{2}+\delta \varepsilon \mu +L^{*}\beta \delta \mu -S^{*}\beta \delta \mu +B^{*}\beta \delta \mu +\delta \gamma \mu \right)\left(\varepsilon +\mu -\beta S^{*}+\gamma \right)\varrho^{\alpha_{3}+\alpha_{1}}\sin\left(\frac{\pi (\alpha_{3}+\alpha_{1})}{2}\right)\\ \; & +\left(\delta \varepsilon +\delta \mu -\beta S^{*}\delta +\delta \gamma \right)\left(\mu^{2}+\beta B^{*}\mu +\beta L^{*}\mu \right)\varrho^{\alpha_{1}+\alpha_{2}}\sin\left(\frac{\pi (\alpha_{2}-\alpha_{1})}{2}\right)\\ \; & -\left(\delta \varepsilon +\delta \mu -\beta S^{*}\delta +\delta \gamma \right)(\mu^{2}+\varepsilon \mu +\beta B^{*}\varepsilon +\beta B^{*}\mu +\beta L^{*}\varepsilon \\ \; & +\beta L^{*}\mu -\beta S^{*}\mu +\gamma \mu )\varrho^{\alpha_{3}+\alpha_{1}}\sin\left(\frac{\pi (\alpha_{1}-\alpha_{3})}{2}\right)\\ \; & -\left(\delta \mu +\beta B^{*}\delta +\beta L^{*}\delta \right)\left(\mu^{2}+\varepsilon \mu -\beta S^{*}\varepsilon -\beta S^{*}\mu +\gamma \mu \right)\varrho^{\alpha_{1}+\alpha_{2}}\sin\left(\frac{\pi (\alpha_{2}-\alpha_{1})}{2}\right)\\ \; & +\left(\delta \mu +\beta B^{*}\delta +\beta L^{*}\delta \right)\left(\mu^{2}+\varepsilon \mu +\beta B^{*}\varepsilon +\beta B^{*}\mu +\beta L^{*}\varepsilon +\beta L^{*}\mu -\beta S^{*}\mu +\gamma \mu \right)\varrho^{\alpha_{2}+\alpha_{3}}\sin\left(\frac{\pi (\alpha_{3}-\alpha_{2})}{2}\right)\\ \; & +\delta (\mu^{3}+\varepsilon \mu^{2}+B^{*}\beta \mu^{2}+L^{*}\beta \mu^{2}-S^{*}\beta \mu^{2}+B^{*}\beta \varepsilon \mu \\ \; & +L^{*}\beta \varepsilon \mu -S^{*}\beta \varepsilon \mu +\gamma \mu^{2})\varrho^{\alpha_{1}+\alpha_{2}}\sin\left(\frac{\pi (\alpha_{1}+\alpha_{2})}{2}\right), \end{array}$$

(93) $$\begin{array}{cc} H_{11R3}\left(\varrho \right)= & \varrho^{\alpha_{1}+\alpha_{2}+\alpha_{3}}\left(\delta \mu^{2}+\delta \varepsilon \mu +L^{*}\beta \delta \mu -S^{*}\beta \delta \mu +B^{*}\beta \delta \mu +\delta \gamma \mu \right)\cos\left(\frac{\pi (\alpha_{1}+\alpha_{2}+\alpha_{3})}{2}\right)\\ \; & +\mu \left(\delta \varepsilon +\delta \mu -\beta S^{*}\delta +\delta \gamma \right)\varrho^{2\alpha_{1}+\alpha_{2}}\cos\left(\frac{\pi \alpha_{2}}{2}\right)\\ \; & +\left(\delta \varepsilon +\delta \mu -\beta S^{*}\delta +\delta \gamma \right)\left(\mu +\beta B^{*}+\beta L^{*}\right)\varrho^{\alpha_{1}+\alpha_{2}+\alpha_{3}}\cos\left(\frac{\pi (\alpha_{2}+\alpha_{3}-\alpha_{1})}{2}\right)\\ \; & +\left(\delta \varepsilon +\delta \mu -\beta S^{*}\delta +\delta \gamma \right)\left(\varepsilon +\mu -\beta S^{*}+\gamma \right)\varrho^{\alpha_{3}+2\alpha_{1}}\cos\left(\frac{\pi \alpha_{3}}{2}\right)\\ \; & +\mu \left(\delta \mu +\beta B^{*}\delta +\beta L^{*}\delta \right)\varrho^{\alpha_{1}+2\alpha_{2}}\cos\left(\frac{\pi \alpha_{1}}{2}\right)\\ \; & +\left(\delta \mu +\beta B^{*}\delta +\beta L^{*}\delta \right)\left(\mu +\beta B^{*}+\beta L^{*}\right)\varrho^{2\alpha_{2}+\alpha_{3}}\cos\left(\frac{\pi \alpha_{3}}{2}\right)\\ \; & +\left(\delta \mu +\beta B^{*}\delta +\beta L^{*}\delta \right)\left(\varepsilon +\mu -\beta S^{*}+\gamma \right)\varrho^{\alpha_{1}+\alpha_{2}+\alpha_{3}}\cos\left(\frac{\pi (\alpha_{3}+\alpha_{1}-\alpha_{2})}{2}\right)\\ \; & +\delta \left(\mu^{2}+\varepsilon \mu -\beta S^{*}\varepsilon -\beta S^{*}\mu +\gamma \mu \right)\varrho^{2\alpha_{1}+\alpha_{2}}\cos\left(\frac{\pi \alpha_{2}}{2}\right)\\ \; & +\delta \left(\mu^{2}+\beta B^{*}\mu +\beta L^{*}\mu \right)\varrho^{\alpha_{1}+2\alpha_{2}}\cos\left(\frac{\pi \alpha_{1}}{2}\right)\\ \; & +\delta \left(\mu^{2}+\varepsilon \mu +\beta B^{*}\varepsilon +\beta B^{*}\mu +\beta L^{*}\varepsilon +\beta L^{*}\mu -\beta S^{*}\mu +\gamma \mu \right)\varrho^{\alpha_{1}+\alpha_{2}+\alpha_{3}}\cos\left(\frac{\pi (\alpha_{1}+\alpha_{2}-\alpha_{3})}{2}\right), \end{array}$$

(94) $$\begin{array}{cc} H_{11I3}\left(\varrho \right)= & -\varrho^{\alpha_{1}+\alpha_{2}+\alpha_{3}}\left(\delta \mu^{2}+\delta \varepsilon \mu +L^{*}\beta \delta \mu -S^{*}\beta \delta \mu +B^{*}\beta \delta \mu +\delta \gamma \mu \right)\sin\left(\frac{\pi (\alpha_{1}+\alpha_{2}+\alpha_{3})}{2}\right)\\ \; & -\mu \left(\delta \varepsilon +\delta \mu -\beta S^{*}\delta +\delta \gamma \right)\varrho^{2\alpha_{1}+\alpha_{2}}\sin\left(\frac{\pi \alpha_{2}}{2}\right)\\ \; & -\left(\delta \varepsilon +\delta \mu -\beta S^{*}\delta +\delta \gamma \right)\left(\mu +\beta B^{*}+\beta L^{*}\right)\varrho^{\alpha_{1}+\alpha_{2}+\alpha_{3}}\sin\left(\frac{\pi (\alpha_{2}+\alpha_{3}-\alpha_{1})}{2}\right)\\ \; & -\left(\delta \varepsilon +\delta \mu -\beta S^{*}\delta +\delta \gamma \right)\left(\varepsilon +\mu -\beta S^{*}+\gamma \right)\varrho^{\alpha_{3}+2\alpha_{1}}\sin\left(\frac{\pi \alpha_{3}}{2}\right)\\ \; & -\mu \left(\delta \mu +\beta B^{*}\delta +\beta L^{*}\delta \right)\varrho^{\alpha_{1}+2\alpha_{2}}\sin\left(\frac{\pi \alpha_{1}}{2}\right)\\ \; & -\left(\delta \mu +\beta B^{*}\delta +\beta L^{*}\delta \right)\left(\mu +\beta B^{*}+\beta L^{*}\right)\varrho^{2\alpha_{2}+\alpha_{3}}\sin\left(\frac{\pi \alpha_{3}}{2}\right)\\ \; & +\left(\delta \mu +\beta B^{*}\delta +\beta L^{*}\delta \right)\left(\varepsilon +\mu -\beta S^{*}+\gamma \right)\varrho^{\alpha_{1}+\alpha_{2}+\alpha_{3}}\sin\left(\frac{\pi (\alpha_{3}+\alpha_{1}-\alpha_{2})}{2}\right)\\ \; & +\delta \left(\mu^{2}+\varepsilon \mu -\beta S^{*}\varepsilon -\beta S^{*}\mu +\gamma \mu \right)\varrho^{2\alpha_{1}+\alpha_{2}}\sin\left(\frac{\pi \alpha_{2}}{2}\right)\\ \; & +\delta \left(\mu^{2}+\beta B^{*}\mu +\beta L^{*}\mu \right)\varrho^{\alpha_{1}+2\alpha_{2}}\sin\left(\frac{\pi \alpha_{1}}{2}\right)\\ \; & +\delta \left(\mu^{2}+\varepsilon \mu +\beta B^{*}\varepsilon +\beta B^{*}\mu +\beta L^{*}\varepsilon +\beta L^{*}\mu -\beta S^{*}\mu +\gamma \mu \right)\varrho^{\alpha_{1}+\alpha_{2}+\alpha_{3}}\sin\left(\frac{\pi (\alpha_{1}+\alpha_{2}-\alpha_{3})}{2}\right), \end{array}$$

(95) $$\begin{array}{cc} H_{11R4}\left(\varrho \right)= & \left(\delta \varepsilon +\delta \mu -\beta S^{*}\delta +\delta \gamma \right)\varrho^{2\alpha_{1}+\alpha_{2}+\alpha_{3}}\cos\left(\frac{\pi (\alpha_{2}+\alpha_{3})}{2}\right)\\ \; & +\left(\delta \mu +\beta B^{*}\delta +\beta L^{*}\delta \right)\varrho^{\alpha_{1}+2\alpha_{2}+\alpha_{3}}\cos\left(\frac{\pi (\alpha_{3}+\alpha_{1})}{2}\right)\\ \; & +\delta \mu \varrho^{2\alpha_{1}+2\alpha_{2}}+\delta \left(\mu +\beta B^{*}+\beta L^{*}\right)\varrho^{\alpha_{1}+2\alpha_{2}+\alpha_{3}}\cos\left(\frac{\pi (\alpha_{1}-\alpha_{3})}{2}\right)\\ \; & +\delta \left(\varepsilon +\mu -\beta S^{*}+\gamma \right)\varrho^{2\alpha_{1}+\alpha_{2}+\alpha_{3}}\cos\left(\frac{\pi (\alpha_{3}-\alpha_{2})}{2}\right), \end{array}$$

and

(96) $$\begin{array}{cc} H_{11I4}\left(\varrho \right)= & -\left(\delta \varepsilon +\delta \mu -\beta S^{*}\delta +\delta \gamma \right)\varrho^{2\alpha_{1}+\alpha_{2}+\alpha_{3}}\sin\left(\frac{\pi (\alpha_{2}+\alpha_{3})}{2}\right)-\left(\delta \mu +\beta B^{*}\delta +\beta L^{*}\delta \right)\varrho^{\alpha_{1}+2\alpha_{2}+\alpha_{3}}\sin\left(\frac{\pi (\alpha_{3}+\alpha_{1})}{2}\right)\\ \; & +\delta \left(\mu +\beta B^{*}+\beta L^{*}\right)\varrho^{\alpha_{1}+2\alpha_{2}+\alpha_{3}}\sin\left(\frac{\pi (\alpha_{1}-\alpha_{3})}{2}\right)-\delta \left(\varepsilon +\mu -\beta S^{*}+\gamma \right)\varrho^{2\alpha_{1}+\alpha_{2}+\alpha_{3}}\sin\left(\frac{\pi (\alpha_{3}-\alpha_{2})}{2}\right). \end{array}$$

Let us solve the Equation (85) for $\upsilon_{2}$, to get

(97) $$\upsilon_{2}=\frac{1}{\varrho_{2}}\left(2\kappa \pi +\arccos\left(-\frac{H_{11R}\left(\varrho_{2}\right)}{|H_{09}\left(\varrho_{2}\right){|}^{2}}\right)\right),$$

with the integer κ taken such that the right-hand side of Equation (97) is positive.

Combining Equations (87)–(97) guarantees that the positive constant $\overset{`}{\upsilon }$ (resp., $\overset{`}{\varrho }$), given by Equation (68) (resp., Equation (69)), is well defined.

With the aid of Equations (19) and (21), we obtain

(98) $$\begin{array}{cc} \frac{\partial }{\partial \xi }▽\left(\xi ;0,\upsilon \right)= & \left(\alpha_{1}+\alpha_{2}+\alpha_{3}\right)\xi^{\alpha_{1}+\alpha_{2}+\alpha_{3}-1}-\delta \upsilon e^{-\upsilon \xi }\xi^{\alpha_{1}+\alpha_{2}}\\ \; & +\left(\alpha_{1}+\alpha_{2}\right)\left(\mu +\delta e^{-\upsilon \xi }\right)\xi^{\alpha_{1}+\alpha_{2}-1}\\ \; & +\left(\alpha_{2}+\alpha_{3}\right)\left(\mu +\beta B^{*}+\beta L^{*}\right)\xi^{\alpha_{2}+\alpha_{3}-1}\\ \; & +\left(\alpha_{3}+\alpha_{1}\right)\left(\varepsilon +\mu -\beta S^{*}+\gamma \right)\xi^{\alpha_{3}+\alpha_{1}-1}\\ \; & -\left(\delta \varepsilon \upsilon e^{-\upsilon \xi }+\delta \mu \upsilon e^{-\upsilon \xi }-\beta S^{*}\delta \upsilon e^{-\upsilon \xi }+\delta \gamma \upsilon e^{-\xi \upsilon }\right)\xi^{\alpha_{1}}\\ \; & +\alpha_{1}\left(\mu^{2}+\varepsilon \mu -\beta S^{*}\varepsilon -\beta S^{*}\mu +\gamma \mu +\delta \varepsilon e^{-\upsilon \xi }+\delta \mu e^{-\upsilon \xi }-\beta S^{*}\delta e^{-\upsilon \xi }+\delta \gamma e^{-\xi \upsilon }\right)\xi^{\alpha_{1}-1}\\ \; & -\left(\delta \mu \upsilon e^{-\upsilon \xi }+\beta B^{*}\delta \upsilon e^{-\upsilon \xi }+\beta L^{*}\delta \upsilon e^{-\upsilon \xi }\right)\xi^{\alpha_{2}}\\ \; & +\alpha_{2}\left(\mu^{2}+\beta B^{*}\mu +\beta L^{*}\mu +\delta \mu e^{-\upsilon \xi }+\beta B^{*}\delta e^{-\upsilon \xi }+\beta L^{*}\delta e^{-\upsilon \xi }\right)\xi^{\alpha_{2}-1}\\ \; & +\alpha_{3}\left(\mu^{2}+\varepsilon \mu +\beta B^{*}\varepsilon +\beta B^{*}\mu +\beta L^{*}\varepsilon +\beta L^{*}\mu -\beta S^{*}\mu +\gamma \mu \right)\xi^{\alpha_{3}-1}\\ \; & -(\delta \mu^{2}\upsilon e^{-\upsilon \xi }+\delta \varepsilon \mu \upsilon e^{-\upsilon \xi }+L^{*}\beta \delta \mu \upsilon e^{-\upsilon \xi }-S^{*}\beta \delta \mu \upsilon e^{-\upsilon \xi }+B^{*}\beta \delta \mu \upsilon e^{-\upsilon \xi }+\delta \gamma \mu \upsilon e^{-\xi \upsilon }), \end{array}$$

and

(99) $$\begin{array}{cc} \frac{\partial }{\partial \upsilon }▽\left(\xi ;0,\upsilon \right)= & -\delta e^{-\upsilon \xi }\xi^{\alpha_{1}+\alpha_{2}+1}\\ \; & -\left(\delta \varepsilon e^{-\upsilon \xi }+\delta \mu e^{-\upsilon \xi }-\beta S^{*}\delta e^{-\upsilon \xi }+\delta \gamma e^{-\xi \upsilon }\right)\xi^{\alpha_{1}+1}\\ \; & -\left(\delta \mu e^{-\upsilon \xi }+\beta B^{*}\delta e^{-\upsilon \xi }+\beta L^{*}\delta e^{-\upsilon \xi }\right)\xi^{\alpha_{2}+1}\\ \; & -(\delta \mu^{2}e^{-\upsilon \xi }+\delta \varepsilon \mu e^{-\upsilon \xi }+L^{*}\beta \delta \mu e^{-\upsilon \xi }-S^{*}\beta \delta \mu e^{-\upsilon \xi }\\ \; & \;+B^{*}\beta \delta \mu e^{-\upsilon \xi }+\delta \gamma \mu e^{-\xi \upsilon })\xi . \end{array}$$

This, together with Equation (98), implies

(100) $${\left(\frac{d\xi }{d\upsilon }\right)}^{-1}=\frac{\frac{\partial }{\partial \xi }▽\left(\xi ;0,\upsilon \right)}{\frac{\partial }{\partial \upsilon }▽\left(\xi ;0,\upsilon \right)}=\frac{\upsilon }{\xi }-\frac{H_{12}\left(\varrho i,\upsilon \right)}{\delta \xi^{2}H_{13}\left(\varrho i,\upsilon \right)},$$

where $H_{12}\left(\varrho i,\upsilon \right)$ and $H_{13}\left(\varrho i,\upsilon \right)$ are given respectively by

(101) $$\begin{array}{cc} H_{12}\left(\xi ,\upsilon \right)= & \left(\alpha_{1}+\alpha_{2}+\alpha_{3}\right)\xi^{\alpha_{1}+\alpha_{2}+\alpha_{3}}+\left(\alpha_{1}+\alpha_{2}\right)\left(\mu +\delta e^{-\upsilon \xi }\right)\xi^{\alpha_{1}+\alpha_{2}}\\ \; & +\left(\alpha_{2}+\alpha_{3}\right)\left(\mu +\beta B^{*}+\beta L^{*}\right)\xi^{\alpha_{2}+\alpha_{3}}+\left(\alpha_{3}+\alpha_{1}\right)\left(\varepsilon +\mu -\beta S^{*}+\gamma \right)\xi^{\alpha_{3}+\alpha_{1}}\\ \; & +\alpha_{1}\left(\mu^{2}+\varepsilon \mu -\beta S^{*}\varepsilon -\beta S^{*}\mu +\gamma \mu +\delta \varepsilon e^{-\upsilon \xi }+\delta \mu e^{-\upsilon \xi }-\beta S^{*}\delta e^{-\upsilon \xi }+\delta \gamma e^{-\xi \upsilon }\right)\xi^{\alpha_{1}}\\ \; & +\alpha_{2}\left(\mu^{2}+\beta B^{*}\mu +\beta L^{*}\mu +\delta \mu e^{-\upsilon \xi }+\beta B^{*}\delta e^{-\upsilon \xi }+\beta L^{*}\delta e^{-\upsilon \xi }\right)\xi^{\alpha_{2}}\\ \; & +\alpha_{3}\left(\mu^{2}+\varepsilon \mu +\beta B^{*}\varepsilon +\beta B^{*}\mu +\beta L^{*}\varepsilon +\beta L^{*}\mu -\beta S^{*}\mu +\gamma \mu \right)\xi^{\alpha_{3}} \end{array}$$

and

(102) $$\begin{array}{cc} H_{13}\left(\xi ,\upsilon \right)= & e^{-\upsilon \xi }\xi^{\alpha_{1}+\alpha_{2}}+\left(\varepsilon e^{-\upsilon \xi }+\mu e^{-\upsilon \xi }-\beta S^{*}e^{-\upsilon \xi }+\gamma e^{-\xi \upsilon }\right)\xi^{\alpha_{1}}\\ \; & +\left(\mu e^{-\upsilon \xi }+\beta B^{*}e^{-\upsilon \xi }+\beta L^{*}e^{-\upsilon \xi }\right)\xi^{\alpha_{2}}\\ \; & +\mu^{2}e^{-\upsilon \xi }+\varepsilon \mu e^{-\upsilon \xi }+L^{*}\beta \mu e^{-\upsilon \xi }-S^{*}\beta \mu e^{-\upsilon \xi }\\ \; & +B^{*}\beta \mu e^{-\upsilon \xi }+\gamma \mu e^{-\xi \upsilon }. \end{array}$$

By straightforward calculations, we have further

(103) $$\begin{array}{cc} H_{12}\left(\varrho i,\upsilon \right)= & \left(\alpha_{1}+\alpha_{2}+\alpha_{3}\right){\left(\varrho i\right)}^{\alpha_{1}+\alpha_{2}+\alpha_{3}}\\ \; & +\left(\alpha_{1}+\alpha_{2}\right)\left(\mu +\delta e^{-\varrho \upsilon i}\right){\left(\varrho i\right)}^{\alpha_{1}+\alpha_{2}}\\ \; & +\left(\alpha_{2}+\alpha_{3}\right)\left(\mu +\beta B^{*}+\beta L^{*}\right){\left(\varrho i\right)}^{\alpha_{2}+\alpha_{3}}\\ \; & +\left(\alpha_{3}+\alpha_{1}\right)\left(\varepsilon +\mu -\beta S^{*}+\gamma \right){\left(\varrho i\right)}^{\alpha_{3}+\alpha_{1}}\\ \; & +\alpha_{1}(\mu^{2}+\varepsilon \mu -\beta S^{*}\varepsilon -\beta S^{*}\mu +\gamma \mu +\delta \varepsilon e^{-\varrho \upsilon i}\\ \; & +\delta \mu e^{-\varrho \upsilon i}-\beta S^{*}\delta e^{-\varrho \upsilon i}+\delta \gamma e^{-\varrho \upsilon i}{)\left(\varrho i\right)}^{\alpha_{1}}\\ \; & +\alpha_{2}(\mu^{2}+\beta B^{*}\mu +\beta L^{*}\mu +\delta \mu e^{-\varrho \upsilon i}\\ \; & +\beta B^{*}\delta e^{-\varrho \upsilon i}+\beta L^{*}\delta e^{-\varrho \upsilon i}{)\left(\varrho i\right)}^{\alpha_{2}}\\ \; & +\alpha_{3}(\mu^{2}+\varepsilon \mu +\beta B^{*}\varepsilon +\beta B^{*}\mu \\ \; & +\beta L^{*}\varepsilon +\beta L^{*}\mu -\beta S^{*}{\mu +\gamma \mu )\left(\varrho i\right)}^{\alpha_{3}}\\ = & \left(\alpha_{1}+\alpha_{2}+\alpha_{3}\right)\varrho^{\alpha_{1}+\alpha_{2}+\alpha_{3}}e^{\frac{\pi i(\alpha_{1}+\alpha_{2}+\alpha_{3})}{2}}\\ \; & +\left(\alpha_{1}+\alpha_{2}\right)\left(\mu +\delta e^{-\varrho \upsilon i}\right)\varrho^{\alpha_{1}+\alpha_{2}}e^{\frac{\pi i(\alpha_{1}+\alpha_{2})}{2}}\\ \; & +\left(\alpha_{2}+\alpha_{3}\right)\left(\mu +\beta B^{*}+\beta L^{*}\right)\varrho^{\alpha_{2}+\alpha_{3}}e^{\frac{\pi i(\alpha_{2}+\alpha_{3})}{2}}\\ \; & +\left(\alpha_{3}+\alpha_{1}\right)\left(\varepsilon +\mu -\beta S^{*}+\gamma \right)\varrho^{\alpha_{3}+\alpha_{1}}e^{\frac{\pi i(\alpha_{3}+\alpha_{1})}{2}}\\ \; & +\alpha_{1}(\mu^{2}+\varepsilon \mu -\beta S^{*}\varepsilon -\beta S^{*}\mu +\gamma \mu +\delta \varepsilon e^{-\varrho \upsilon i}\\ \; & +\delta \mu e^{-\varrho \upsilon i}-\beta S^{*}\delta e^{-\varrho \upsilon i}+\delta \gamma e^{-\varrho \upsilon i})\varrho^{\alpha_{1}}e^{\frac{\pi \alpha_{1}i}{2}}\\ \; & +\alpha_{2}(\mu^{2}+\beta B^{*}\mu +\beta L^{*}\mu +\delta \mu e^{-\varrho \upsilon i}\\ \; & +\beta B^{*}\delta e^{-\varrho \upsilon i}+\beta L^{*}\delta e^{-\varrho \upsilon i})\varrho^{\alpha_{2}}e^{\frac{\pi \alpha_{2}i}{2}}\\ \; & +\alpha_{3}(\mu^{2}+\varepsilon \mu +\beta B^{*}\varepsilon +\beta B^{*}\mu +\beta L^{*}\varepsilon \\ \; & +\beta L^{*}\mu -\beta S^{*}\mu +\gamma \mu )\varrho^{\alpha_{3}}e^{\frac{\pi \alpha_{3}i}{2}} \end{array}$$

and

(104) $$\begin{array}{cc} H_{13}\left(\varrho i,\upsilon \right)= & e^{-\upsilon \varrho i}{\left(\varrho i\right)}^{\alpha_{1}+\alpha_{2}}+\left(\varepsilon e^{-\upsilon \varrho i}+\mu e^{-\upsilon \varrho i}-\beta S^{*}e^{-\upsilon \varrho i}+\gamma e^{-\upsilon \varrho i}\right){\left(\varrho i\right)}^{\alpha_{1}}\\ \; & +\left(\mu e^{-\upsilon \varrho i}+\beta B^{*}e^{-\upsilon \varrho i}+\beta L^{*}e^{-\upsilon \varrho i}\right){\left(\varrho i\right)}^{\alpha_{2}}\\ \; & +(\mu^{2}e^{-\upsilon \varrho i}+\varepsilon \mu e^{-\upsilon \varrho i}+L^{*}\beta \mu e^{-\upsilon \varrho i}-S^{*}\beta \mu e^{-\upsilon \varrho i}+B^{*}\beta \mu e^{-\upsilon \varrho i}+\gamma \mu e^{-\upsilon \varrho i})\\ = & \varrho^{\alpha_{1}+\alpha_{2}}e^{-\upsilon \varrho i}e^{\frac{\pi i(\alpha_{1}+\alpha_{2})}{2}}+\left(\varepsilon e^{-\upsilon \varrho i}+\mu e^{-\upsilon \varrho i}-\beta S^{*}e^{-\upsilon \varrho i}+\gamma e^{-\upsilon \varrho i}\right)\varrho^{\alpha_{1}}e^{\frac{\pi \alpha_{1}i}{2}}\\ \; & +\left(\mu e^{-\upsilon \varrho i}+\beta B^{*}e^{-\upsilon \varrho i}+\beta L^{*}e^{-\upsilon \varrho i}\right)\varrho^{\alpha_{2}}e^{\frac{\pi \alpha_{2}i}{2}}\\ \; & +\mu^{2}e^{-\upsilon \varrho i}+\varepsilon \mu e^{-\upsilon \varrho i}+L^{*}\beta \mu e^{-\upsilon \varrho i}-S^{*}\beta \mu e^{-\upsilon \varrho i}+B^{*}\beta \mu e^{-\upsilon \varrho i}+\gamma \mu e^{-\upsilon \varrho i}. \end{array}$$

Employing Equation (100) as a central tool, a series of routine calculations leads to

(105) $$\begin{array}{cc} {\left({\left.\frac{d\xi }{d\upsilon }\right|}_{\xi =\overset{`}{\varrho }i,\upsilon =\overset{`}{\upsilon }}\right)}^{-1}= & -\frac{\overset{`}{\upsilon }i}{\overset{`}{\varrho }}+\frac{\bar{H_{13}\left(\overset{`}{\varrho }i,\overset{`}{\upsilon }\right)}H_{12}\left(\overset{`}{\varrho }i,\overset{`}{\upsilon }\right)}{\delta {\left(\overset{`}{\varrho }\right)}^{2}{\left|H_{13}\left(\overset{`}{\varrho }i,\overset{`}{\upsilon }\right)\right|}^{2}}\\ = & \frac{W_{4}\left(\overset{`}{\varrho },\overset{`}{\upsilon }\right)}{\delta {\left(\overset{`}{\varrho }\right)}^{2}{\left|H_{13}\left(\overset{`}{\varrho }i,\overset{`}{\upsilon }\right)\right|}^{2}}+i\left(\frac{H_{14}\left(\overset{`}{\varrho },\overset{`}{\upsilon }\right)}{\delta {\left(\overset{`}{\varrho }\right)}^{2}{\left|H_{13}\left(\overset{`}{\varrho }i,\overset{`}{\upsilon }\right)\right|}^{2}}-\frac{\overset{`}{\upsilon }}{\overset{`}{\varrho }}\right), \end{array}$$

in which $W_{4}\left(\varrho ,\upsilon \right)$ (see Equation (67) for the definition of $W_{4}\left(\varrho ,\upsilon \right)$), $H_{12}\left(\xi ,\upsilon \right)$, $H_{13}\left(\xi ,\upsilon \right)$, and $H_{14}\left(\varrho ,\upsilon \right)$ satisfy the following relation

(106) $$\bar{H_{13}\left(\varrho i,\upsilon \right)}H_{12}\left(\varrho i,\upsilon \right)=W_{4}\left(\varrho ,\upsilon \right)+iH_{14}\left(\varrho ,\upsilon \right),$$

and furthermore, $H_{14}\left(\varrho ,\upsilon \right)$ is given as follows

(107) $$\begin{array}{cc} H_{14}= & \left(\alpha_{1}+\alpha_{2}+\alpha_{3}\right)\varrho^{2\alpha_{1}+2\alpha_{2}+\alpha_{3}}\sin\left(\frac{\pi \alpha_{3}}{2}+\upsilon \varrho \right)+\mu \left(\alpha_{1}+\alpha_{2}\right)\varrho^{2\alpha_{1}+2\alpha_{2}}\sin\left(\upsilon \varrho \right)\\ \; & +\left(\alpha_{2}+\alpha_{3}\right)\left(\mu +\beta B^{*}+\beta L^{*}\right)\varrho^{\alpha_{1}+2\alpha_{2}+\alpha_{3}}\sin\left(\frac{\pi (\alpha_{3}-\alpha_{1})}{2}+\upsilon \varrho \right)\\ \; & +\left(\alpha_{3}+\alpha_{1}\right)\left(\varepsilon +\mu -\beta S^{*}+\gamma \right)\varrho^{2\alpha_{1}+\alpha_{2}+\alpha_{3}}\sin\left(\frac{\pi (\alpha_{3}-\alpha_{2})}{2}+\upsilon \varrho \right)\\ \; & +\alpha_{1}\varrho^{2\alpha_{1}+\alpha_{2}}\left(\left(\mu^{2}+\varepsilon \mu -\beta S^{*}\varepsilon -\beta S^{*}\mu +\gamma \mu \right)\sin\left(\upsilon \varrho -\frac{\pi \alpha_{2}}{2}\right)-\left(\delta \varepsilon +\delta \mu -\beta S^{*}\delta +\delta \gamma \right)\sin\left(\frac{\pi \alpha_{2}}{2}\right)\right)\\ \; & +\alpha_{2}\left(\mu^{2}+\beta B^{*}\mu +\beta L^{*}\mu \right)\varrho^{\alpha_{1}+2\alpha_{2}}\sin\left(\upsilon \varrho -\frac{\pi \alpha_{1}}{2}\right)-\alpha_{2}\left(\delta \mu +\beta B^{*}\delta +\beta L^{*}\delta \right)\varrho^{\alpha_{1}+2\alpha_{2}}\sin\left(\frac{\pi \alpha_{1}}{2}\right)\\ \; & +\alpha_{3}\left(\mu^{2}+\varepsilon \mu +\beta B^{*}\varepsilon +\beta B^{*}\mu +\beta L^{*}\varepsilon +\beta L^{*}\mu -\beta S^{*}\mu +\gamma \mu \right)\varrho^{\alpha_{1}+\alpha_{2}+\alpha_{3}}\sin\left(\frac{\pi (\alpha_{3}-\alpha_{1}-\alpha_{2})}{2}+\upsilon \varrho \right)\\ \; & +\left(\varepsilon +\mu -\beta S^{*}+\gamma \right)\left(\alpha_{1}+\alpha_{2}+\alpha_{3}\right)\varrho^{2\alpha_{1}+\alpha_{2}+\alpha_{3}}\sin\left(\frac{\pi (\alpha_{2}+\alpha_{3})}{2}+\upsilon \varrho \right)\\ \; & +\left(\varepsilon +\mu -\beta S^{*}+\gamma \right)\left(\alpha_{1}+\alpha_{2}\right)\left(\mu \varrho^{2\alpha_{1}+\alpha_{2}}\sin\left(\frac{\pi \alpha_{2}}{2}+\upsilon \varrho \right)+\delta \varrho^{2\alpha_{1}+\alpha_{2}}\sin\left(\frac{\pi \alpha_{2}}{2}\right)\right)\\ \; & +\left(\varepsilon +\mu -\beta S^{*}+\gamma \right)\left(\alpha_{2}+\alpha_{3}\right)\left(\mu +\beta B^{*}+\beta L^{*}\right)\varrho^{\alpha_{1}+\alpha_{2}+\alpha_{3}}\sin\left(\frac{\pi (\alpha_{2}+\alpha_{3}-\alpha_{1})}{2}+\upsilon \varrho \right)\\ \; & +\left(\alpha_{3}+\alpha_{1}\right){\left(\varepsilon +\mu -\beta S^{*}+\gamma \right)}^{2}\varrho^{\alpha_{3}+2\alpha_{1}}\sin\left(\frac{\pi \alpha_{3}}{2}+\upsilon \varrho \right)+\alpha_{1}\left(\varepsilon +\mu -\beta S^{*}+\gamma \right)(\mu^{2}+\varepsilon \mu -\beta S^{*}\varepsilon -\beta S^{*}\mu \\ \; & +\gamma \mu )\varrho^{2\alpha_{1}}\sin\left(\upsilon \varrho \right)+\alpha_{2}\left(\varepsilon +\mu -\beta S^{*}+\gamma \right)\left(\mu^{2}+\beta B^{*}\mu +\beta L^{*}\mu \right)\varrho^{\alpha_{1}+\alpha_{2}}\sin\left(\frac{\pi (\alpha_{2}-\alpha_{1})}{2}+\upsilon \varrho \right)\\ \; & +\alpha_{2}\left(\varepsilon +\mu -\beta S^{*}+\gamma \right)\left(\delta \mu +\beta B^{*}\delta +\beta L^{*}\delta \right)\varrho^{\alpha_{1}+\alpha_{2}}\sin\left(\frac{\pi (\alpha_{2}-\alpha_{1})}{2}\right)\\ \; & +\alpha_{3}\left(\varepsilon +\mu -\beta S^{*}+\gamma \right)\left(\mu^{2}+\varepsilon \mu +\beta B^{*}\varepsilon +\beta B^{*}\mu +\beta L^{*}\varepsilon +\beta L^{*}\mu -\beta S^{*}\mu +\gamma \mu \right)\varrho^{\alpha_{3}+\alpha_{1}}\sin\left(\frac{\pi (\alpha_{3}-\alpha_{1})}{2}+\upsilon \varrho \right)\\ \; & +\left(\mu +\beta B^{*}+\beta L^{*}\right)\left(\alpha_{1}+\alpha_{2}+\alpha_{3}\right)\varrho^{\alpha_{1}+2\alpha_{2}+\alpha_{3}}\sin\left(\frac{\pi (\alpha_{3}+\alpha_{1})}{2}+\upsilon \varrho \right)\\ \; & +\left(\mu +\beta B^{*}+\beta L^{*}\right)\left(\alpha_{1}+\alpha_{2}\right)\varrho^{\alpha_{1}+2\alpha_{2}}\left(\mu \sin\left(\frac{\pi \alpha_{1}}{2}+\upsilon \varrho \right)+\delta \sin\left(\frac{\pi \alpha_{1}}{2}\right)\right)\\ \; & +\left(\alpha_{2}+\alpha_{3}\right){\left(\mu +\beta B^{*}+\beta L^{*}\right)}^{2}\varrho^{2\alpha_{2}+\alpha_{3}}\sin\left(\frac{\pi \alpha_{3}}{2}+\upsilon \varrho \right)\\ \; & +\left(\mu +\beta B^{*}+\beta L^{*}\right)\left(\alpha_{3}+\alpha_{1}\right)\left(\varepsilon +\mu -\beta S^{*}+\gamma \right)\varrho^{\alpha_{1}+\alpha_{2}+\alpha_{3}}\sin\left(\frac{\pi (\alpha_{3}+\alpha_{1}-\alpha_{2})}{2}+\upsilon \varrho \right)\\ \; & +\alpha_{1}\left(\mu +\beta B^{*}+\beta L^{*}\right)\left(\mu^{2}+\varepsilon \mu -\beta S^{*}\varepsilon -\beta S^{*}\mu +\gamma \mu \right)\varrho^{\alpha_{1}+\alpha_{2}}\sin\left(\frac{\pi (\alpha_{1}-\alpha_{2})}{2}+\upsilon \varrho \right)\\ \; & +\alpha_{1}\left(\mu +\beta B^{*}+\beta L^{*}\right)\left(\delta \varepsilon +\delta \mu -\beta S^{*}\delta +\delta \gamma \right)\varrho^{\alpha_{1}+\alpha_{2}}\sin\left(\frac{\pi (\alpha_{1}-\alpha_{2})}{2}\right)+\alpha_{2}\mu {\left(\mu +\beta B^{*}+\beta L^{*}\right)}^{2}\varrho^{2\alpha_{2}}\sin\left(\upsilon \varrho \right)\\ \; & +\alpha_{3}\left(\mu +\beta B^{*}+\beta L^{*}\right)\left(\mu^{2}+\varepsilon \mu +\beta B^{*}\varepsilon +\beta B^{*}\mu +\beta L^{*}\varepsilon +\beta L^{*}\mu -\beta S^{*}\mu +\gamma \mu \right)\varrho^{\alpha_{2}+\alpha_{3}}\sin\left(\frac{\pi (\alpha_{3}-\alpha_{2})}{2}+\upsilon \varrho \right)\\ \; & +\left(\mu^{2}+\varepsilon \mu +L^{*}\beta \mu -S^{*}\beta \mu +B^{*}\beta \mu +\gamma \mu \right)\left(\alpha_{1}+\alpha_{2}+\alpha_{3}\right)\varrho^{\alpha_{1}+\alpha_{2}+2\alpha_{3}}\sin\left(\frac{\pi (\alpha_{1}+\alpha_{2}+\alpha_{3})}{2}+\upsilon \varrho \right)\\ \; & +\left(\mu^{2}+\varepsilon \mu +L^{*}\beta \mu -S^{*}\beta \mu +B^{*}\beta \mu +\gamma \mu \right)\left(\alpha_{1}+\alpha_{2}\right)\varrho^{\alpha_{1}+\alpha_{2}+\alpha_{3}}\left(\mu \sin\left(\frac{\pi (\alpha_{1}+\alpha_{2})}{2}+\upsilon \varrho \right)+\delta \sin\left(\frac{\pi (\alpha_{1}+\alpha_{2})}{2}\right)\right)\\ \; & +\left(\mu^{2}+\varepsilon \mu +L^{*}\beta \mu -S^{*}\beta \mu +B^{*}\beta \mu +\gamma \mu \right)\left(\alpha_{2}+\alpha_{3}\right)\left(\mu +\beta B^{*}+\beta L^{*}\right)\varrho^{\alpha_{2}+2\alpha_{3}}\sin\left(\frac{\pi (\alpha_{2}+\alpha_{3})}{2}+\upsilon \varrho \right)\\ \; & +\left(\mu^{2}+\varepsilon \mu +L^{*}\beta \mu -S^{*}\beta \mu +B^{*}\beta \mu +\gamma \mu \right)\left(\alpha_{3}+\alpha_{1}\right)\left(\varepsilon +\mu -\beta S^{*}+\gamma \right)\varrho^{2\alpha_{3}+\alpha_{1}}\sin\left(\frac{\pi (\alpha_{3}+\alpha_{1})}{2}+\upsilon \varrho \right)\\ \; & +\alpha_{1}\left(\mu^{2}+\varepsilon \mu +L^{*}\beta \mu -S^{*}\beta \mu +B^{*}\beta \mu +\gamma \mu \right)\left(\mu^{2}+\varepsilon \mu -\beta S^{*}\varepsilon -\beta S^{*}\mu +\gamma \mu \right)\varrho^{\alpha_{3}+\alpha_{1}}\sin\left(\frac{\pi \alpha_{1}}{2}+\upsilon \varrho \right)\\ \; & +\alpha_{1}\left(\mu^{2}+\varepsilon \mu +L^{*}\beta \mu -S^{*}\beta \mu +B^{*}\beta \mu +\gamma \mu \right)\left(\delta \varepsilon +\delta \mu -\beta S^{*}\delta +\delta \gamma \right)\varrho^{\alpha_{3}+\alpha_{1}}\sin\left(\frac{\pi \alpha_{1}}{2}\right)\\ \; & +\left(\mu^{2}+\varepsilon \mu +L^{*}\beta \mu -S^{*}\beta \mu +B^{*}\beta \mu +\gamma \mu \right)(\alpha_{2}\left(\mu +\beta B^{*}+\beta L^{*}\right)\varrho^{\alpha_{2}+\alpha_{3}}\left(\mu \sin\left(\frac{\pi \alpha_{2}}{2}+\upsilon \varrho \right)+\delta \sin\left(\frac{\pi \alpha_{2}}{2}\right)\right)\\ \; & +\alpha_{3}\left(\mu^{2}+\varepsilon \mu +\beta B^{*}\varepsilon +\beta B^{*}\mu +\beta L^{*}\varepsilon +\beta L^{*}\mu -\beta S^{*}\mu +\gamma \mu \right)\varrho^{2\alpha_{3}}\sin\left(\frac{\pi \alpha_{3}}{2}+\upsilon \varrho \right)). \end{array}$$

The positivity of $W_{4}\left(\overset{`}{\varrho },\overset{`}{\upsilon }\right)$, together with Equation (105), yields

(108) Re(dξdυξ=ϱ`i,υ=υ`)−1=W4(ϱ`,υ`)γ(ϱ`)2|H13(ϱ`i,υ`)|2>0,

which, in analogy with Equation (65), further implies

(109) Re(dξdυξ=ϱ`i,υ=υ`)=dξdυξ=ϱ`i,υ=υ`2Re(dξdυξ=ϱ`i,υ=υ`)−1>0.

This, together with the definition (68) of $\overset{`}{\upsilon }$, brings the proof of Theorem 2 to a close. □

## 4. Numerical Simulations

In Section 3, we established two bifurcation results for DFSLB (3), identifying conditions under which the system undergoes qualitative changes in its dynamical behavior. In this section, we aim to visualize these bifurcation results through a series of numerical simulations. Specifically, by solving DFSLB (110) and DFSLB (116) under various initial conditions and different values of the time delay parameters $\tau_{1}$ and $\tau_{2}$, we illustrate both the asymptotic stability of the endemic equilibrium when $\tau_{1}$ and $\tau_{2}$ fall below the critical thresholds and the emergence of periodic oscillations when $\tau_{1}$ and $\tau_{2}$ exceed these thresholds. The simulations not only confirm the theoretical predictions but also provide a clear depiction of the system’s dynamical evolution in both the time domain and phase space.

**Example** **1.** *Let $\tau_{1}$ be an arbitrary nonnegative real number. We perform some bifurcation analyses of the following DFSLB.*

(110) Dt0.95CS(t)=14−23S(t)(L(t)+B(t))+14L(t−τ1)+34B(t)−14S(t)fort∈R+,Dt0.97CL(t)=23S(t)(L(t)+B(t))−14L(t−τ1)−14L(t)−14L(t)fort∈R+,Dt0.99CB(t)=14L(t)−34B(t)−14B(t)fort∈R+.

It can be readily observed that DFSLB (110) corresponds precisely to DFSLB (3) with the parameter values $\alpha_{1}=0.95$, $\alpha_{2}=0.97$, $\alpha_{3}=0.99$, $\gamma =\frac{1}{4}$, $\delta =\frac{3}{4}$, $\mu =\frac{1}{4}$, $\varepsilon =\frac{1}{4}$, $\beta =\frac{2}{3}$, and $\tau_{2}=0$. From these observations and straightforward calculations, we find that, apart from the trivial virus-free equilibrium (1,0,0), DFSLB (110) has a unique endemic equilibrium $\left(\frac{9}{10},\frac{2}{25},\frac{1}{50}\right)$.

With the aid of DFSLB (15), we linearize DFSLB (110) at the endemic equilibrium $\left(\frac{9}{10},\frac{2}{25},\frac{1}{50}\right)$ to obtain

(111) Dt0.95CS(t)=−1960S(t)−35L(t)+14L(t−τ1)+320B(t)fort∈R+,Dt0.97CL(t)=115S(t)+110L(t)−14L(t−τ1)+35B(t)fort∈R+,Dt0.99CB(t)=14L(t)−B(t)fort∈R+.

Guided by the properties of the linearized computer virus model (111), we analyze DFSLB (110) and find that there exists a critical threshold $\overset{´}{\vartheta }$ (estimated to be approximately 4.9713 through numerical computations performed in MATLAB) such that the endemic equilibrium $\left(\frac{9}{10},\frac{2}{25},\frac{1}{50}\right)$ of DFSLB (110) is at least locally asymptotically stable for $\tau_{2}=0$ and $\tau_{1}\in \left[0,\overset{´}{\vartheta }\right)$. Moreover, for DFSLB (110), a family of periodic orbits bifurcate from the endemic equilibrium $\left(\frac{9}{10},\frac{2}{25},\frac{1}{50}\right)$ when $\tau_{2}=0$ and $\tau_{1}$ reaches the critical value $\overset{´}{\vartheta }$.

**Figure 2:**
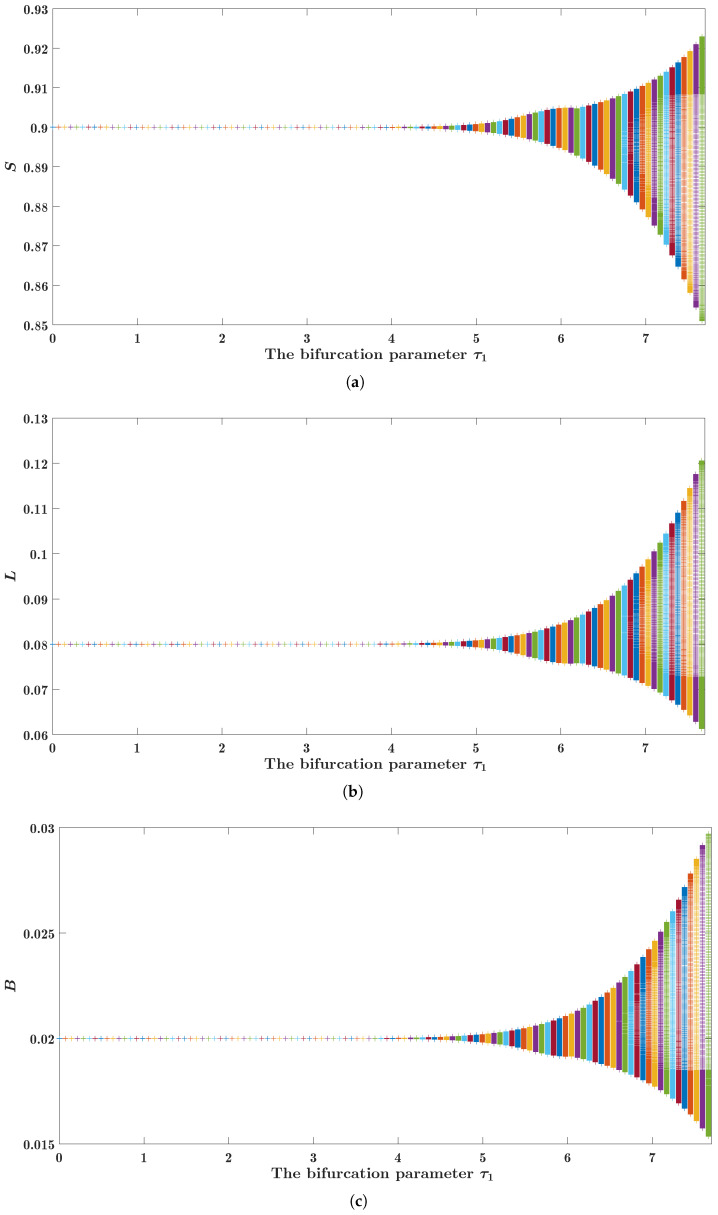
Numerical simulations and graphical illustrations demonstrating the existence of a Hopf bifurcation in DFSLB (3) with $\tau_{2}=0$. The bifurcation diagrams of DFSLB (110) in terms of *S*, *L*, and *B* components are presented in (**a**), (**b**), and (**c**), respectively.

As claimed in Theorem 1, let us visualize the Hopf bifurcation phenomenon in DFSLB (3) with $\tau_{2}=0$. To this end, we first present the bifurcation diagrams of DFSLB (110) with respect to the *S*, *L*, and *B*; see Figure 2. To provide a clearer visualization of the bifurcation phenomenon, we investigate the long-time behavior of solutions of DFSLB (110) for two representative cases: $\tau_{1}=4.921$, strictly below $\overset{´}{\vartheta }$, and $\tau_{1}=5.4131$, exceeding $\overset{´}{\vartheta }$. To better visualize the stability of the endemic equilibrium $\left(\frac{9}{10},\frac{2}{25},\frac{1}{50}\right)$, we introduce for DFSLB (110), with $\tau_{2}=0$ and $\tau_{1}=4.921$, the following four distinct initial conditions.

(112) $$\left\{\begin{array}{ccc} S\left(t\right)=\frac{8937}{10,000} & \; & \mathrm{for}\;t\in [-\tau_{1},0],\\ L\left(t\right)=\frac{817}{10,000} & \; & \mathrm{for}\;t\in [-\tau_{1},0],\\ B\left(t\right)=\frac{246}{10,000} & \; & \mathrm{when}\;t=0, \end{array}\right.$$

(113) $$\left\{\begin{array}{ccc} S\left(t\right)=\frac{19}{20} & \; & \mathrm{for}\;t\in [-\tau_{1},0],\\ L\left(t\right)=\frac{9}{1000} & \; & \mathrm{for}\;t\in [-\tau_{1},0],\\ B\left(t\right)=\frac{41}{1000} & \; & \mathrm{when}\;t=0, \end{array}\right.$$

(114) $$\left\{\begin{array}{ccc} S\left(t\right)=\frac{24}{25} & \; & \mathrm{for}\;t\in [-\tau_{1},0],\\ L\left(t\right)=\frac{3}{100} & \; & \mathrm{for}\;t\in [-\tau_{1},0],\\ B\left(t\right)=\frac{1}{100} & \; & \mathrm{when}\;t=0, \end{array}\right.$$

and

(115) $$\left\{\begin{array}{ccc} S\left(t\right)=\frac{4}{5} & \; & \mathrm{for}\;t\in [-\tau_{1},0],\\ L\left(t\right)=\frac{13}{100} & \; & \mathrm{for}\;t\in [-\tau_{1},0],\\ B\left(t\right)=\frac{7}{100} & \; & \mathrm{when}\;t=0. \end{array}\right.$$

**Figure 3:**
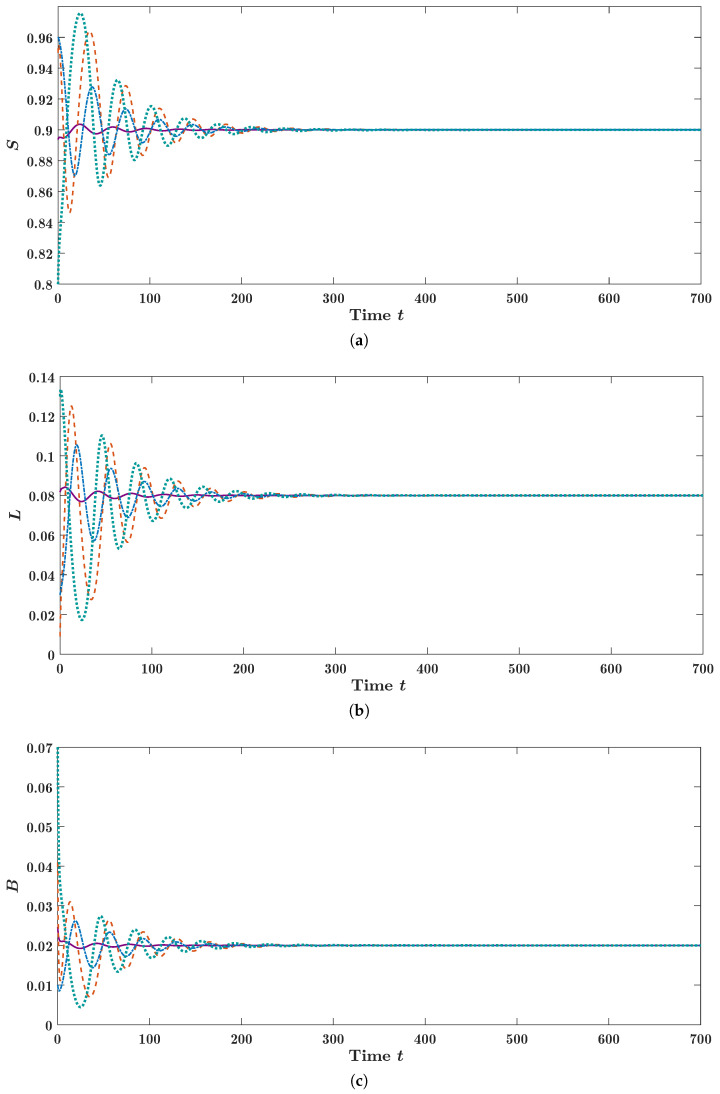
Numerical and graphical illustrations of the existence of the endemic equilibrium of DFSLB (3) with $\tau_{2}=0$, along with its asymptotic stability. The solid curves correspond to the solutions of DFSLB (110) associated with the initial condition (112). The long-dashed curves represent the solutions of DFSLB (110) subject to the initial condition (113), while the dash-dotted curves correspond to those associated with (114). The dotted curves represent the solutions of DFSLB (110) subject to the initial condition (115). The *S*-, *L*-, and *B*-components of the solutions of DFSLB (110), with $\tau_{1}=4.921$ (less than $\overset{´}{\vartheta }$) and subject to the initial conditions (112)–(115), are depicted in panels (**a**–**c**), respectively.

**Figure 4:**
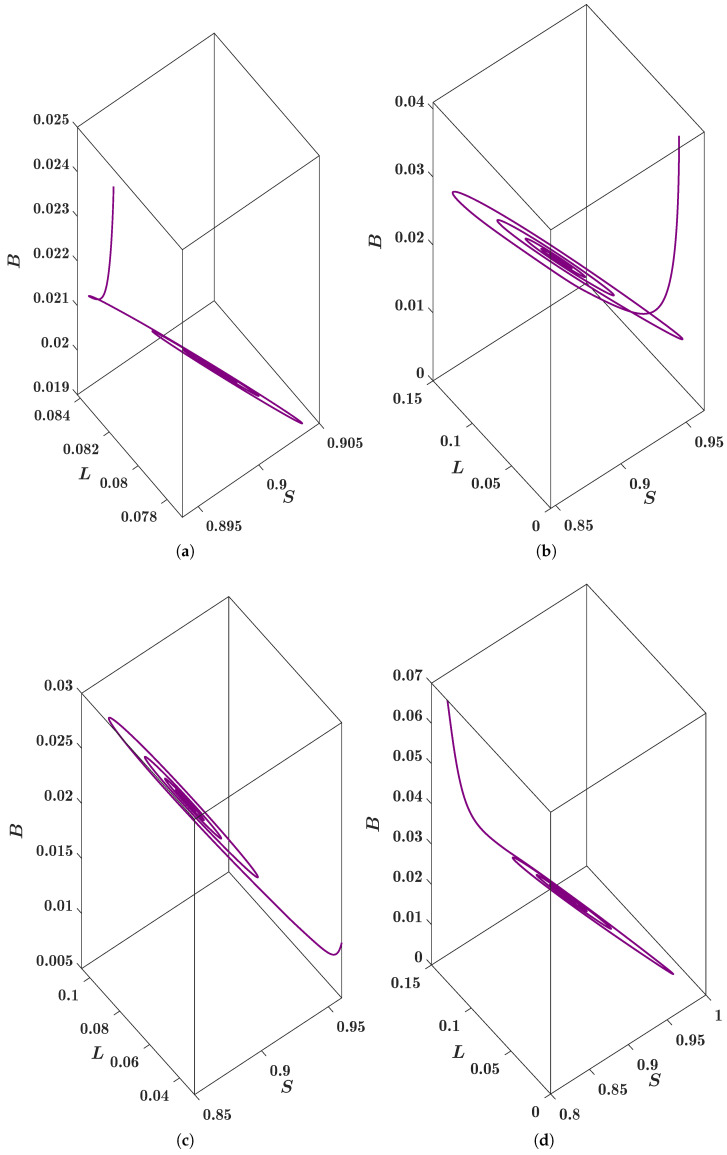
Numerical and graphical illustrations of the existence of the endemic equilibrium of DFSLB (3) with $\tau_{2}=0$, along with its asymptotic stability. The trajectories (S(t),L(t),B(t)) of DFSLB (110), with $\tau_{1}=4.921$ (which lies below the threshold $\overset{´}{\vartheta }$), and under the initial conditions (112)–(115), are depicted in panels (**a**–**d**), respectively.

**Figure 5:**
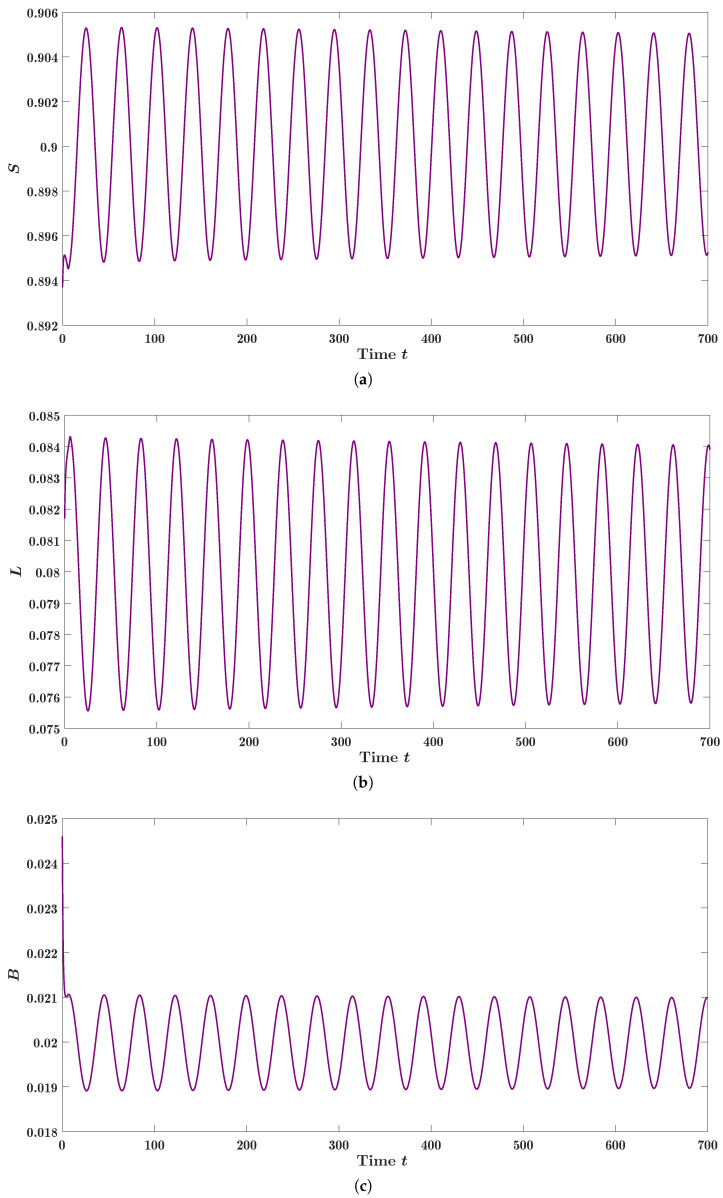
Numerical and graphical illustrations of the existence of periodic orbits in DFSLB (3) with $\tau_{2}=0$, along with their asymptotic stability. The *S*-, *L*-, and *B*-components of the solution (S(t),L(t),B(t)) to DFSLB (110), with $\tau_{1}=5.4131$ (exceeding the threshold $\overset{´}{\vartheta }$) and subject to the initial conditions (112), are depicted in panels (**a**–**c**), respectively.

**Figure 6:**
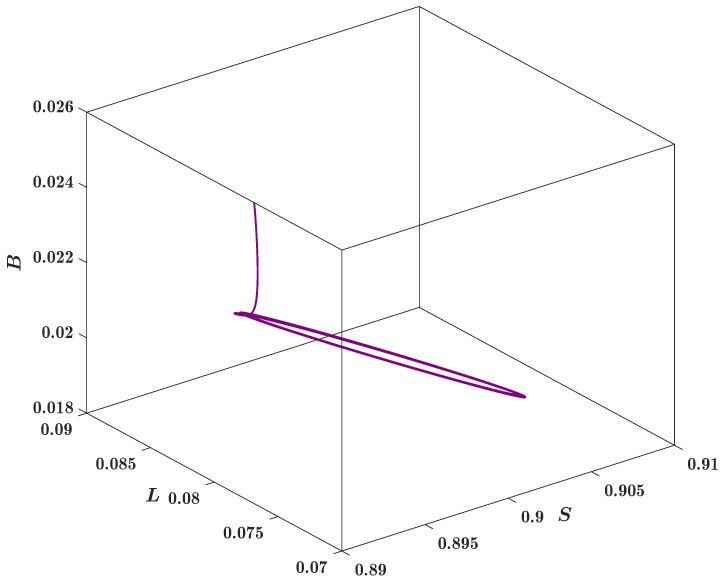
Numerical and graphical illustrations of the existence of periodic orbits in DFSLB (3) with $\tau_{2}=0$, along with their asymptotic stability. The depicted space curve represents the trajectory of the solution (S(t),L(t),B(t)) to DFSLB (110), with $\tau_{1}=5.4131$ (exceeding the threshold $\overset{´}{\vartheta }$), under the initial condition (112).

To investigate the dynamics of DFSLB (110), numerical simulations were carried out in MATLAB for the four distinct initial conditions (112)–(115). Figure 3 and Figure 4 depict solutions of DFSLB (110), with $\tau_{2}=0$ and $\tau_{1}=4.921$, corresponding to the four distinct initial conditions (112)–(115). By reviewing these figures, one can find that the solutions of DFSLB (110) approach the endemic equilibrium $\left(\frac{9}{10},\frac{2}{25},\frac{1}{50}\right)$ as time *t* escapes to infinity, confirming the Lyapunov (more precisely, asymptotic) stability of the endemic equilibrium $\left(\frac{9}{10},\frac{2}{25},\frac{1}{50}\right)$ when $\tau_{2}=0$ and $\tau_{1}=4.921$, lying below the threshold $\overset{´}{\vartheta }$. In contrast, when $\tau_{1}$ exceeds $\overset{´}{\vartheta }$, the dynamics of DFSLB (110) change significantly. Figure 5 and Figure 6 present the solution of DFSLB (110), with $\tau_{2}=0$ and $\tau_{1}=5.4131$, subject to the initial condition (112). It is evident from these simulations that the solutions no longer converge to the endemic equilibrium; instead, they evolve toward a nontrivial closed orbit in the phase space over long time intervals, indicating the emergence of sustained oscillations. Based on a series of numerical simulations of the solutions to DFSLB (110) for $\tau_{2}=0$ and for various values of $\tau_{1}$ under different initial conditions, together with a careful examination of Figure 2, Figure 3, Figure 4, Figure 5 and Figure 6, we conclude that a Hopf bifurcation occurs at $\tau_{1}=\overset{´}{\vartheta }$ in DFSLB (110) with $\tau_{2}=0$.

In summary, these numerical experiments illustrate our theoretical results, namely Theorem 1, which states that DFSLB (3) with $\tau_{2}=0$ undergoes a Hopf bifurcation as the time delay $\tau_{1}$ passes through the critical threshold $\overset{´}{\vartheta }$.

**Example** **2.** *Let $\tau_{2}$ be an arbitrary nonnegative real number. We perform some bifurcation analyses of the following DFSLB.*

(116) Dt0.95CS(t)=14−23S(t)(L(t)+B(t))+14L(t)+34B(t−τ2)−14S(t)fort∈R+,Dt0.97CL(t)=23S(t)(L(t)+B(t))−14L(t)−14L(t)−14L(t)fort∈R+,Dt0.99CB(t)=14L(t)−34B(t−τ2)−14B(t)fort∈R+.

It can be concluded that DFSLB (116) corresponds precisely to DFSLB (3) with the parameter values $\alpha_{1}=0.95$, $\alpha_{2}=0.97$, $\alpha_{3}=0.99$, $\gamma =\frac{1}{4}$, $\delta =\frac{3}{4}$, $\mu =\frac{1}{4}$, $\varepsilon =\frac{1}{4}$, $\beta =\frac{2}{3}$, and $\tau_{1}=0$. By virtue of straightforward calculations, we find that, apart from the trivial virus-free equilibrium (1,0,0), DFSLB (116) has a unique endemic equilibrium $\left(\frac{9}{10},\frac{2}{25},\frac{1}{50}\right)$.

We linearize DFSLB (116) at the equilibrium $\left(\frac{9}{10},\frac{2}{25},\frac{1}{50}\right)$ to obtain

(117) Dt0.95CS(t)=−1960S(t)−720L(t)−35B(t)+34B(t−τ2)fort∈R+,Dt0.97CL(t)=115S(t)−320L(t)+35B(t)fort∈R+,Dt0.99CB(t)=14L(t)−34B(t−τ2)−14B(t)fort∈R+,

Guided by the properties of the linearized computer virus model (117), we analyze DFSLB (116) and find that there exists a critical threshold $\overset{`}{\upsilon }$ (estimated to be approximately 3.2017 through numerical computations performed in MATLAB (R2016a)) such that the endemic equilibrium $\left(\frac{9}{10},\frac{2}{25},\frac{1}{50}\right)$ of DFSLB (116) is at least locally asymptotically stable for $\tau_{1}=0$ and $\tau_{2}\in \left[0,\overset{`}{\upsilon }\right)$. Moreover, for DFSLB (116), a family of periodic orbits bifurcate from the endemic equilibrium $\left(\frac{9}{10},\frac{2}{25},\frac{1}{50}\right)$ when $\tau_{1}=0$ and $\tau_{2}$ reaches the critical value $\overset{`}{\upsilon }$.

**Figure 7:**
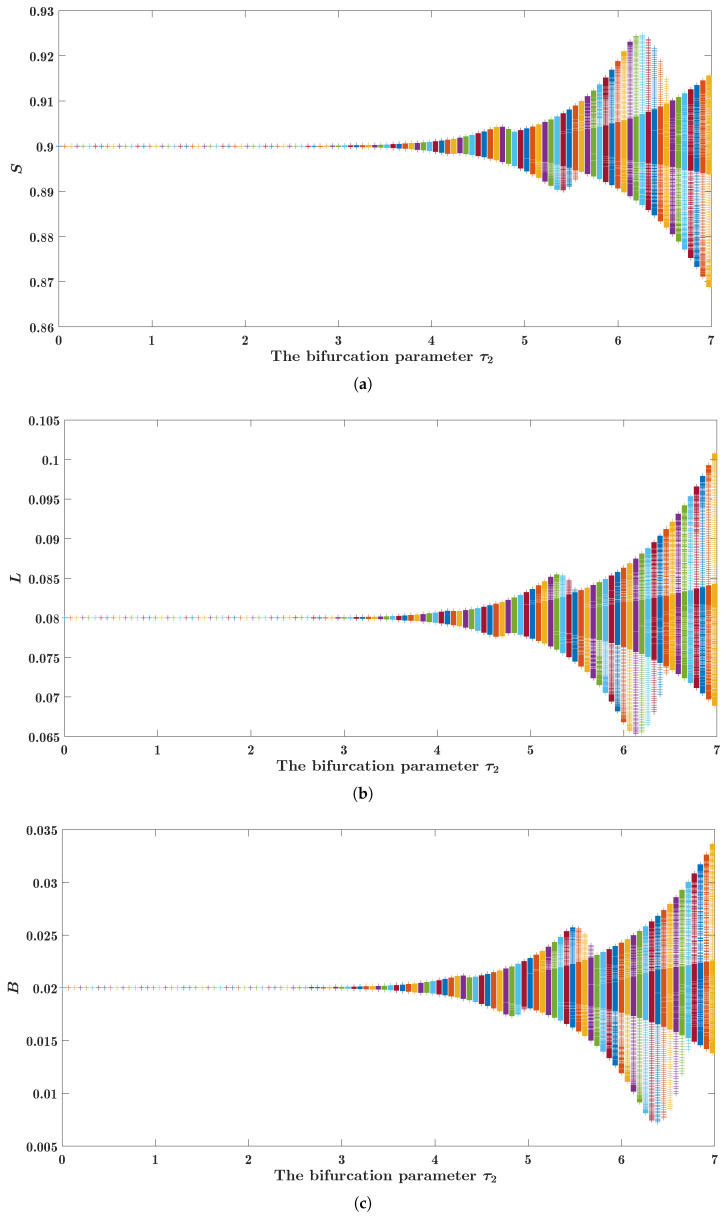
Numerical simulations and graphical illustrations demonstrating the existence of a Hopf bifurcation in DFSLB (3) with $\tau_{1}=0$. The bifurcation diagrams of DFSLB (116) in terms of *S*, *L*, and *B* components are presented in (**a**), (**b**), and (**c**), respectively.

As claimed in Theorem 2, let us visualize the Hopf bifurcation phenomenon in DFSLB (3) with $\tau_{1}=0$. To this end, we first present the bifurcation diagrams of DFSLB (116) with respect to the *S*, *L*, and *B*; see Figure 7. To provide a clearer visualization of the bifurcation phenomenon, we investigate the long-time behavior of solutions of DFSLB (116) for two representative cases: $\tau_{2}=3.1948$, strictly below $\overset{`}{\upsilon }$, and $\tau_{2}=3.4556$, exceeding $\overset{`}{\upsilon }$. To better visualize the stability of the endemic equilibrium $\left(\frac{9}{10},\frac{2}{25},\frac{1}{50}\right)$, we introduce for DFSLB (116), with $\tau_{1}=0$ and $\tau_{2}=3.1948$, the following four distinct initial conditions

(118) $$\left\{\begin{array}{ccc} S\left(t\right)=\frac{8937}{10,000} & \; & \mathrm{for}\;t\in [-\tau_{2},0],\\ L\left(t\right)=\frac{817}{10,000} & \; & \mathrm{when}\;t=0,\\ B\left(t\right)=\frac{246}{10,000} & \; & \mathrm{for}\;t\in [-\tau_{2},0], \end{array}\right.$$

(119) $$\left\{\begin{array}{ccc} S\left(t\right)=\frac{97}{100} & \; & \mathrm{for}\;t\in [-\tau_{2},0],\\ L\left(t\right)=\frac{29}{1000} & \; & \mathrm{when}\;t=0,\\ B\left(t\right)=\frac{1}{1000} & \; & \mathrm{for}\;t\in [-\tau_{2},0], \end{array}\right.$$

(120) $$\left\{\begin{array}{ccc} S\left(t\right)=\frac{947}{1000} & \; & \mathrm{for}\;t\in [-\tau_{2},0],\\ L\left(t\right)=\frac{43}{1000} & \; & \mathrm{when}\;t=0,\\ B\left(t\right)=\frac{1}{100} & \; & \mathrm{for}\;t\in [-\tau_{2},0], \end{array}\right.$$

and

(121) $$\left\{\begin{array}{ccc} S\left(t\right)=\frac{83}{100} & \; & \mathrm{for}\;t\in [-\tau_{2},0],\\ L\left(t\right)=\frac{13}{100} & \; & \mathrm{when}\;t=0,\\ B\left(t\right)=\frac{1}{25} & \; & \mathrm{for}\;t\in [-\tau_{2},0]. \end{array}\right.$$

**Figure 8:**
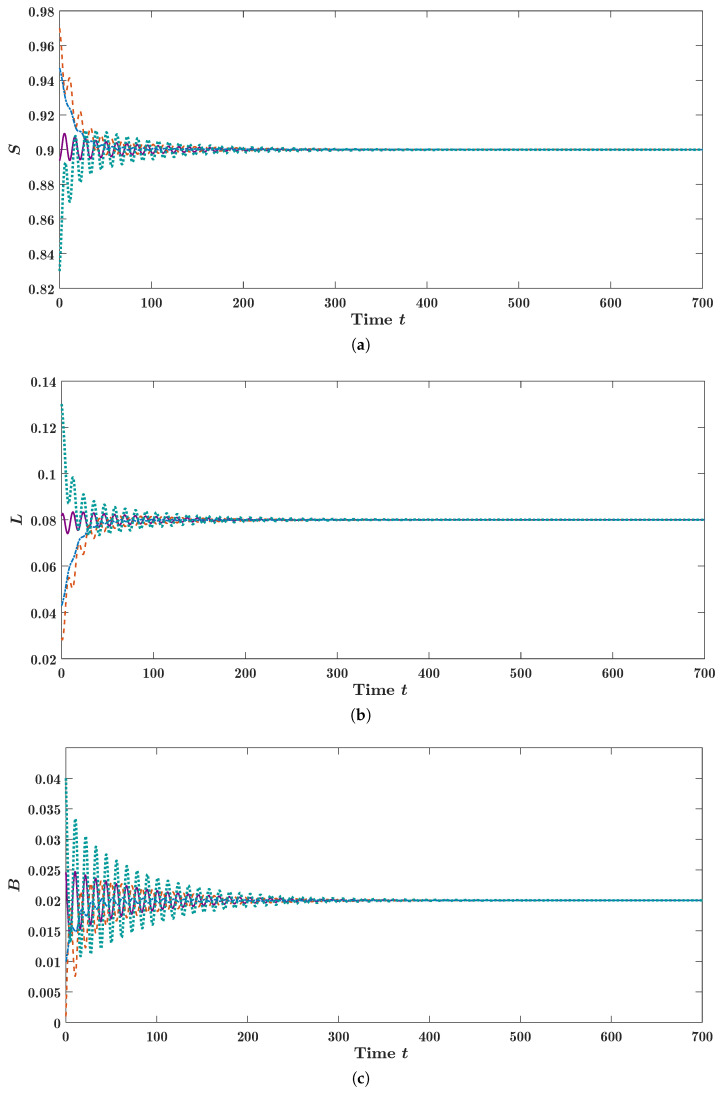
Numerical and graphical illustrations of the existence of the endemic equilibrium of DFSLB (3) with $\tau_{1}=0$, along with its asymptotic stability. The solid curves correspond to the solutions of DFSLB (116) associated with the initial condition (118). The long-dashed curves represent the solutions of DFSLB (116) subject to the initial condition (119), while the dash-dotted curves correspond to those associated with (120). The dotted curves represent the solutions of DFSLB (116) subject to the initial condition (121). The *S*-, *L*-, and *B*-components of the solutions of DFSLB (116), with $\tau_{2}=3.1948$ (less than $\overset{`}{\upsilon }$) and subject to the initial conditions (118)–(121), are depicted in panels (**a**–**c**), respectively.

**Figure 9:**
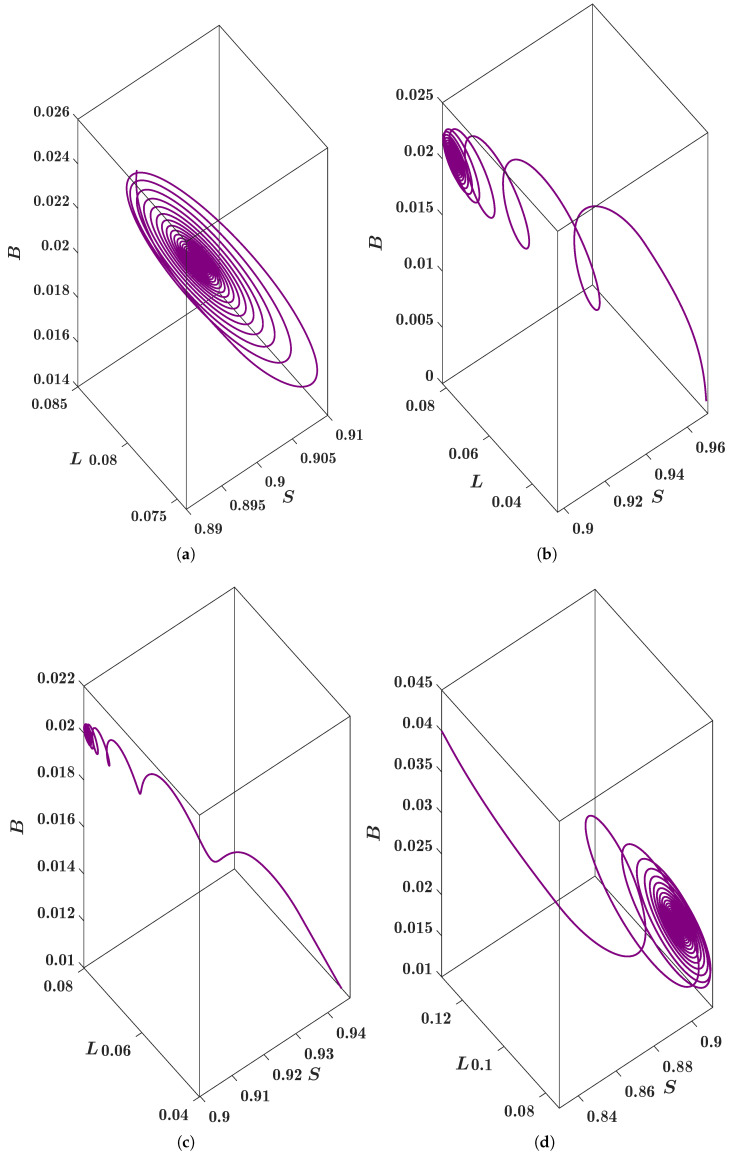
Numerical and graphical illustrations of the existence of the endemic equilibrium of DFSLB (3) with $\tau_{1}=0$, along with its asymptotic stability. The trajectories (S(t),L(t),B(t)) of DFSLB (116), with $\tau_{2}=3.1948$ (less than $\overset{`}{\upsilon }$) and subject to the initial conditions (118)–(121), are depicted in panels (**a**–**d**), respectively.

**Figure 10:**
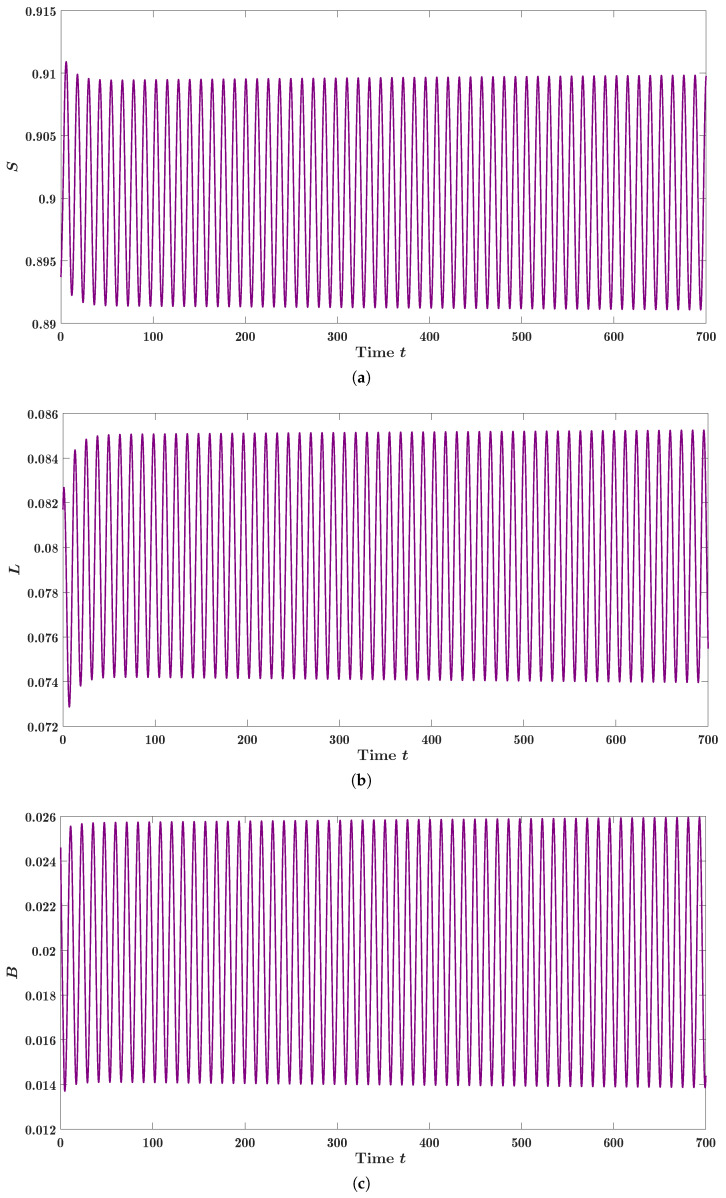
Numerical and graphical illustrations of the existence of periodic orbits in DFSLB (3) with $\tau_{1}=0$, along with their asymptotic stability. The *S*-, *L*-, and *B*-components of the solution (S(t),L(t),B(t)) to DFSLB (116), with $\tau_{2}=3.4556$ (exceeding the threshold $\overset{`}{\upsilon }$) and subject to the initial conditions (118), are depicted in panels (**a**–**c**), respectively.

**Figure 11:**
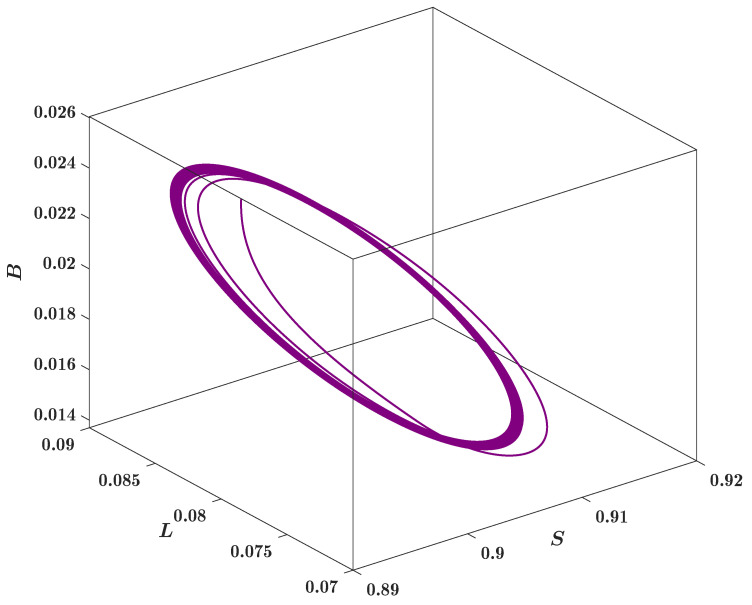
Numerical and graphical illustrations of the existence of periodic orbits in DFSLB (3) with $\tau_{1}=0$, along with their asymptotic stability. The depicted space curve represents the trajectory of the solution (S(t),L(t),B(t)) to DFSLB (116), with $\tau_{2}=3.4556$ (exceeding the threshold $\overset{`}{\upsilon }$), under the initial condition (118).

To investigate the dynamics of DFSLB (116), numerical simulations were carried out in MATLAB for the four distinct initial conditions (118)–(121). Figure 8 and Figure 9 depict the solutions of DFSLB (116), with $\tau_{1}=0$ and $\tau_{2}=3.1948$, corresponding to the four distinct initial conditions (118)–(121). By reviewing these figures, one can find that the solutions of DFSLB (116) approach the endemic equilibrium $\left(\frac{9}{10},\frac{2}{25},\frac{1}{50}\right)$ as time *t* escapes to infinity, confirming the Lyapunov (more precisely, asymptotic) stability of the endemic equilibrium $\left(\frac{9}{10},\frac{2}{25},\frac{1}{50}\right)$ when $\tau_{1}=0$ and $\tau_{2}=3.1948$, lying below the threshold $\overset{`}{\upsilon }$. In contrast, when $\tau_{2}$ exceeds $\overset{`}{\upsilon }$, the dynamics of DFSLB (116) change significantly. Figure 10 and Figure 11 present the solution of DFSLB (116), with $\tau_{1}=0$ and $\tau_{2}=3.4556$, subject to the initial condition (118). It is evident from these simulations that the solutions no longer converge to the endemic equilibrium; instead, they evolve toward a nontrivial closed orbit in the phase space over long time intervals, indicating the emergence of sustained oscillations. Based on a series of numerical simulations of the solutions to DFSLB (116) for $\tau_{1}=0$ and for various values of $\tau_{2}$ under different initial conditions, together with a careful examination of Figure 7, Figure 8, Figure 9, Figure 10 and Figure 11, we conclude that a Hopf bifurcation occurs at $\tau_{2}=\overset{`}{\upsilon }$ in DFSLB (116) with $\tau_{1}=0$.

In summary, these numerical experiments illustrate our theoretical results, namely Theorem 2, which states that DFSLB (3) with $\tau_{1}=0$ undergoes a Hopf bifurcation as the time delay $\tau_{2}$ passes through the critical threshold $\overset{`}{\upsilon }$.

**Remark** **4.** *Fractional-order differential equations have received increasing attention due to their remarkable capability of characterizing memory and hereditary properties inherent in many complex models, which has stimulated the development of various numerical schemes for the accurate and efficient approximation of fractional-order models [60,62,63,64]. In this study, considering the specific structure of the proposed system (3), the fractional Euler method is employed for numerical simulations. The temporal discretization step size is chosen as Δt=0.001 to achieve a reliable balance between numerical accuracy and computational efficiency. The method is constructed based on the Volterra integral formulation of the Caputo fractional derivative, where the fractional integral is approximated by a discrete convolution involving power-law memory kernels [60]. Compared with more sophisticated algorithms (see [62,63,64], for example), the fractional Euler method possesses a relatively simple implementation and requires moderate computational cost, making it particularly suitable for exploring the dynamical evolution and bifurcation behaviors of the considered system (3). The convergence of the afore-mentioned numerical simulations was validated by considering several temporal discretization step sizes, including Δt=0.001, 0.0009, 0.0007, and 0.0005.*

## 5. Concluding Remarks

By reviewing vast references, we concluded that classical epidemic models for computer virus propagation typically adopt integer-order dynamics and a single time delay-simplifying assumptions that overlook the inherent memory effects and asynchronous transmission processes characteristic of real-world network environments; see [1,50], among other references. To address this limitation, the present study extends the Susceptible–Latent–Breaking-Out framework through the incorporation of Caputo fractional derivatives of incommensurate orders, paired with two distinct time delays: One linked to the virus infection rate and the other to the latent period of infected nodes; see Equation (3) for detail. The primary objective of this research is to examine how the combined effects of fractional exponents and multiple delays modulate the stability of the endemic equilibrium, and to identify the specific conditions under which sustained oscillatory behavior emerges in virus prevalence. The two time delays were chosen as bifurcation parameters, and the characteristic equation derived from linearization of the model around its endemic equilibrium was subjected to rigorous analytical investigation. This analysis reveals that each delay possesses a well-defined critical threshold: each delay is maintained below its respective threshold, the endemic equilibrium retains asymptotic stability; conversely, exceeding any single delay threshold triggers a Hopf bifurcation, which generates periodic oscillations in virus prevalence. The precise conditions governing the occurrence of this bifurcation are detailed in Section 3 (Theorems 1 and 2). Numerical simulations, presented in Section 4, validate the analytical findings, with computed bifurcation points exhibiting close consistency with the theoretically derived values.

To prove our main results, Theorems 1 and 2, we follow an idea widely used in the literature [23,25,26,33,46], and the procedure parallels that of [28]. Nevertheless, compared with [28], the nonlinearity in our model is more complicated and thus brings greater difficulty to the bifurcation analysis.

These results indicate that fractional-order delay models for computer virus propagation can exhibit Hopf bifurcations under conditions analogous to those observed in integer-order systems, though the critical thresholds are distinctly modulated by the fractional exponents. From a practical standpoint, the findings offer specific, actionable guidance for the design of containment strategies in large-scale interconnected networks, specifically emphasizing the necessity of accounting for both the magnitudes of transmission delays and the order of fractional derivatives employed to model memory effects in virus propagation processes. Future work will build on the present model by incorporating targeted control strategies, stochastic perturbations, and realistic network topologies, which are expected to yield richer dynamical behaviors and more practical insights for virus containment.

## Data Availability

All data supporting the findings of this study are included in the article.
